# Toxicity Evaluation of Nano-Sized Particles by Analysis of mtDNA Content and Expression Levels of Genes Required for mtDNA Maintenance: A Meta-Analysis of Pre-Clinical Studies

**DOI:** 10.3390/antiox15070848

**Published:** 2026-07-04

**Authors:** Qiwen Liu, Yunxia Liang, Dongli Xie, Yiming Xu, Dianliang Wang, Xiaogang Luo

**Affiliations:** 1 College of Textile and Clothing Engineering, Soochow University, 199 Ren-Ai Road, Suzhou 215123, China; 2 PPM Institute of Functional Materials, Poly Plastic Masterbatch (Suzhou) Co., Ltd., 29 Xujiaguan Road, Suzhou 215144, China

**Keywords:** environmental pollutants, nano-sized particles, mtDNA, mitochondria

## Abstract

Mitochondrial alterations, including mitochondrial DNA (mtDNA) loss and defects in maintenance pathways, have been recognized as an important driver for toxic effects of environmental pollutants. Therefore, exposure to nano-sized particles (1–100 nm in diameter; a new source of environmental pollution) may also result in these mitochondrial impairments; however, controversial results have been reported. Available studies collected from three electronic databases through July 2025 were pooled for a comprehensive assessment. Meta-analysis of 19 in vitro studies (69 datasets) showed exposure to nano-sized particles significantly reduced mtDNA content [standardized mean difference = −1.08; *p*-value = 0.001). The expression levels of mtDNA-encoded (ND1, COX1,2, CYTB, ATP6), mitochondrial biogenesis (SIRT1, PGC-1α, TFAM) and fusion genes (MFN1, MFN2, OPA1) were found to be significantly down-regulated, while fission genes DRP1 and FIS1 were up-regulated following nano-sized particle exposure after meta-analysis of corresponding in vitro and in vivo studies. Accordingly, mtDNA depletion and expression disruption in mtDNA-encoded and maintenance genes may represent important contributors to nano-sized particle exposure-induced diseases.

## 1. Introduction

Mitochondria are semi-autonomous organelles present in almost all eukaryotes that perform multiple biological functions for maintenance of cell survival, with a primary role of producing energy (adenosine triphosphate, ATP) via oxidative phosphorylation (OXPHOS) [1]. OXPHOS is carried out by protein complexes of the electron transport chain. Most of the OXPHOS system proteins are encoded by nuclear DNA (nDNA; 79) and a minor proportion are encoded by mitochondrial DNA [mtDNA; 13, consisting of seven from complex I (NADH dehydrogenase subunits: ND1/2/3/4/4/L5/6), one from complex III (cytochrome B: CYTB), three from complex IV (cytochrome oxidase subunits: COX1-3) and two from complex V (ATP synthase: ATP6/8)] [2,3]. In addition to the structural OXPHOS subunit genes, mtDNA also encodes two ribosome RNAs (rRNAs) and 22 transfer RNAs (tRNAs) for protein synthesis within mitochondria [3]. Compared to the nuclear genome, mtDNA is more vulnerable to damages from excessive reactive oxygen species (ROS) because it is not packaged into nucleosomes with protective histones and is located in close proximity to the OXPHOS system of the inner mitochondrial membrane (the major source of ROS) [4,5]. Therefore, mtDNA may represent a preferential target of environmental toxicants to induce the onset of diseases in various organs with high energy requirements [6,7].

There are several nDNA-encoded proteins involved in mtDNA maintenance. The polymerase gamma (POLG) gene encodes the DNA polymerase that is responsible for the replication of mtDNA [8,9]. Mitochondrial transcription factor A (TFAM) is a DNA-binding protein that activates mtDNA transcription to generate the RNA primer required for the initiation of mtDNA replication. The transcriptional coactivator peroxisome proliferator-activated receptor-γ coactivator 1alpha (PGC-1α) promotes the synthesis of mtDNA and proteins and generation of new mitochondria by activating its downstream transcription factor nuclear respiratory factor-1 (NRF1) or 2 (NRF2), and subsequently stimulating the transcription of TFAM [10,11]. Adenosine monophosphate-activated protein kinase (AMPK) can either directly phosphorylate PGC-1α or activate sirtuin 1 (SIRT1) through increasing NAD+ levels followed by de-acetylation of PGC-1α [10,12]. Furthermore, mitochondria are highly dynamic organelles that undergo cyclic fusion and fission to preserve mitochondrial structure and facilitate content exchange (e.g., mtDNA). Mitochondrial fission is controlled by the GTPase dynamin-related protein 1 (DNM1L/DRP1) and tail-anchored adaptors mitochondrial fission 1 (FIS1) and fission factor (MFF) [13], while mitochondrial fusion is modulated by both outer- (mitofusin 1 and 2: MFN1 and MFN2) and inner-mitochondrial-membrane machinery (GTPase optic atrophy protein 1, OPA1) [14]. Therefore, dysregulation of these genes involved in mitochondrial biogenesis (AMPK/SIRT1-PGC-1α-NRF1/2-TFAM), fusion (MFN1, MFN2, OPA1) and fission (DRP1, FIS1, MFF) may also serve as important toxic effects of environmental health hazards.

With the ever-increasing application of nanomaterials [including metal nanoparticles (NPs), metal oxide NPs, quantum dots (QDs), carbon nanotubes (CNTs), grapheme] and plastics in industrial fields and daily life [15,16], nano-sized particles (≤100 nm) are inevitably released into the environment and adversely affect the health of living organisms. In recent years, extensive studies have shown that nano-sized particles can enter the bodies of animals and humans through inhalation, ingestion, and dermal contact routes and then accumulate in all tissues (blood, lung, liver, kidney, spleen, heart, brain and testis) to trigger related injuries [17,18,19,20,21]. Although the toxic mechanisms of nano-sized particles remain incompletely understood, oxidative stress-induced mitochondrial dysfunction has aroused increasing concern [22,23,24]. Also, there have been meta-analyses that confirm exposure to nano-sized particles can significantly increase levels of ROS [25,26], indicating the mtDNA content and maintenance genes may be subsequently altered. This hypothesis had been supported in some studies; however, the conclusions regarding different nano-sized particle types seem to be controversial: Qi et al. [27] reported that exposure to silica NPs (SiNPs) significantly reduced mtDNA content, down-regulated PGC-1α, NRF1, and MFN1 and up-regulated DRP1, FIS1 and MFN2 in the human liver cell line L-02, but had no significant influence on OPA1 and TFAM. Guo et al. found that SiNPs-treated human umbilical vein endothelial cells (HUVECs) exhibited significantly decreased levels of mtDNA, PGC-1a, NRF1, TFAM, and OPA1 and elevated levels of DRP1, FIS1 and MFN2, but no significant change was observed for MFN1 [28]. MtDNA content in supernatants of primary lung cells treated with silver NPs (AgNPs) was not significantly different from that of control cells [29]. Exposure of zinc oxide NPs (ZnONPs) to human neuroblastoma SH-SY5Y cells did not significantly affect the expression levels of PGC-1α, MFN1, MFN2, MFF or DRP1 proteins [30]. These findings highlight the necessity of comprehensively investigating the associations between mtDNA-related indicators and nano-sized particle exposure to achieve more solid evidence.

The existing meta-analysis literature has verified that exposure to NPs could induce nDNA damages [31,32]; however, mitochondrial genotoxicity was rarely studied and no definite conclusions have been drawn. To fill this gap in our knowledge, we conducted a meta-analysis to evaluate whether exposure to nano-sized particles significantly changed the levels of mtDNA content and mtDNA maintenance-related genes via integrating all evidence from pre-clinical research. The results may provide a better understanding of the significance of mtDNA damage in nano-sized particle-induced toxicity.

## 2. Materials and Methods

### 2.1. Search Strategy

This meta-analysis was conducted by following the guidelines in the Preferred Reporting Items for Systematic Reviews and Meta-Analysis (PRISMA) 2020 statement [33]. The literature search was done in the three electronic databases of PubMed, EMBASE and Cochrane Library to retrieve potentially eligible articles published before July 15, 2025, without language restrictions. Keyword search items included (“nanomaterials” OR “nanoparticle” OR “carbon nanotube” OR “graphene” OR “quantum dot” OR “nanoplastic” OR “microplastic”) AND (“mitochondrial DNA copy number” OR “mtDNA copy number” OR “mitochondrial DNA content” OR “mtDNA content” OR “mitochondrial dysfunction” OR “mitochondrial biogenesis” OR “mitochondrial dynamic” OR “mitochondrial fusion” OR “mitochondrial fission” OR “mitochondrial transcription factor A” OR “mtTFA” OR “TFAM” OR “mitochondrial transcription factor B” OR “mtTFB” OR “TFBM” OR “peroxisome proliferator-activated receptor gamma coactivator-1α” OR “PGC-1α” OR “nuclear respiratory factor” OR “NRF1” OR “NRF2” OR “mitofusins” OR “MFN1” OR “MFN2” OR “optic atrophy 1” OR “OPA1” OR “dynamin-related protein 1” OR “DRP1” OR “mitochondrial fission 1” OR “FIS1” OR “mitochondrial fission factor” OR “MFF” OR “polymerase gamma” OR “POLG” OR “sirtuin 1” OR “SIRT1” OR “AMP-activated protein kinase” OR “AMPK”). Additionally, the reference lists of review articles and relevant original articles were scrutinized to uncover possibly omitted studies.

### 2.2. Selection Criteria

The eligibility criteria, guided by the population, intervention, comparison, outcomes and study design strategy [34], included: (1) population: humans, cells or animals; (2) intervention: an experimental group exposed to nano-sized particles; (3) comparison: a control group that did not receive treatment with nano-sized particles; (4) outcomes: mean and standard deviation of mtDNA-related indicators (including mtDNA content and expression levels of mtDNA-encoded and mtDNA maintenance genes) provided or could be estimated; and (5) study design: controlled trials.

The exclusion criteria were: (1) duplications; (2) non-original research (e.g., case reports, reviews, meta-analyses, meeting abstracts, letters or protocols); (3) non-cell or animal studies; (4) studies with data unavailability for related indicators; (5) indicators that were measured in less than three studies; (6) studies without a control group; (7) studies with particle size > 100 nm or unknown; (8) studies with nano-sized materials as drug carriers for treatment of diseases, but not as toxic reagents; (9) studies retracted or not peer-reviewed (e.g., preprints); and (10) studies on other irrelevant topics. Two authors independently screened the literature search results to identify eligible articles and potential discrepancies were settled in consultation with a third reviewer.

### 2.3. Data Extraction

Relevant data regarding the characteristics of each eligible article were independently extracted by two authors and summarized in an Excel sheet, including the first author, publication year, study location, cell or animal type, nano-sized particle type, size, exposure dose and duration, sample size of experimental and control groups, outcome data, data assay method and the tissue source of samples. The mean and standard deviation for each variable were directly collected from tables/texts or estimated from figures using the GetData Graph Digitizer (v2.26; https://getdata-graph-digitizer.software.informer.com/)(accessed on 10 December 2024). Data measured at different doses or durations in each study were all extracted in order to evaluate the dose- or duration-dependent effects. Any disagreements during the data extraction process were discussed with the third reviewer.

### 2.4. Quality Assessment

The methodological quality of included in vitro and in vivo studies was judged by the toxicological data reliability assessment tool (Toxrtool) [35,36]. The Toxrtool scale comprises 18 evaluation items for in vitro studies and 21 evaluation criteria for in vivo studies, with each graded as “1” (criterion met) or “0” (criterion not met or not reported) during analysis. Studies with more than 11 (in vitro) or 13 (in vivo) points were deemed reliable [35,36]. Quality assessment was performed independently by two assessors; in case of discrepancies, the third assessor was involved.

### 2.5. Statistical Analysis

Extracted data in the Excel sheet were exported to Stata software (v15.0; Stata Corp., College Station, TX, USA) for data analyses. The effect size (ES) was expressed as the standardized mean difference (SMD) accompanied by a 95% confidence interval (CI). The Z-statistic was applied to analyze the ES and a *P*_ES_-value < 0.05 indicated statistical significance. The heterogeneity among studies was examined using Cochran’s Q chi-squared test and the *I*^2^ statistic. The existence of heterogeneity (H) was defined as *P*_H_ < 0.1 and *I*^2^ > 50%. A random-effects model was adopted for pooled analyses of heterogeneous studies, whereas a fixed-effects model was used for homogeneous studies. Subgroup analysis was performed to investigate potential sources of heterogeneity for variables with at least ten articles included, which was stratified by country (Asian or non-Asian), cell type (different species, tissue sources), animal type (different species), nano-sized particle type, dose (in vitro: ≤50 µg/mL/µg/cm^2^ or >50 µg/mL/µg/cm^2^; in vivo: ≤50 mg/kg or >50 mg/kg), exposure duration (in vitro: ≤24 h or >24 h; in vivo: ≤14 d or >14 d), mtDNA genes used for analysis of mtDNA content, expression levels according to the assay method [mRNA (reverse transcription polymerase chain reaction, RT-PCR) or protein (Western blotting, WB; immunohistochemistry, IHC; immunocytochemistry, ICC; immunofluorescence, IF; enzyme-linked immunosorbent assay, ELISA)] and tissue sources of animals. Moreover, a multivariable meta-regression analysis was also conducted for indicators in the subgroup analysis to explore possible heterogenous factors. The probability of publication bias (PB) was estimated by Egger’s linear regression test. A trim-and-fill method was employed to adjust for significant PB (*P*_PB_ < 0.05). Sensitivity analyses were implemented using the leave-one-out approach to gauge the robustness of the pooled results.

## 3. Results

### 3.1. Study Selection

**Figure 1:**
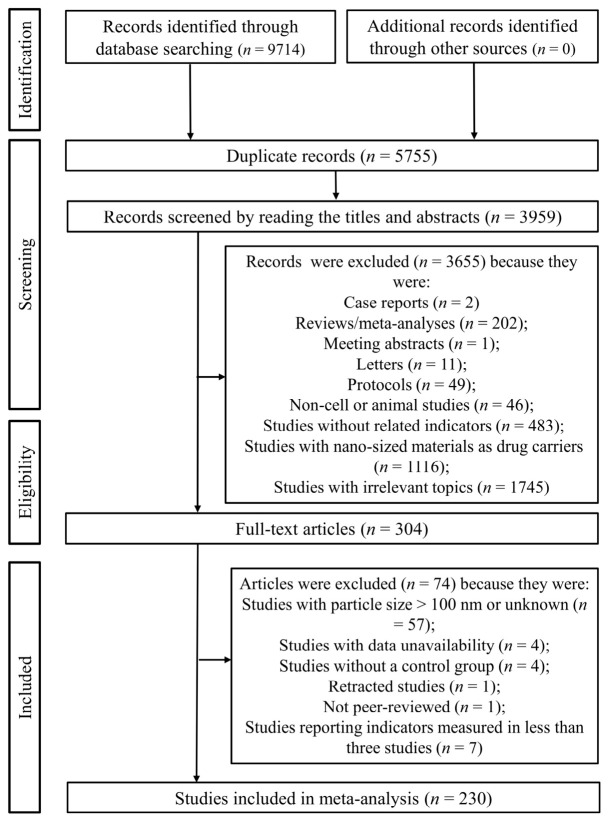
Flow diagram of literature identification according to the PRISMA statement.

The search of the three online databases retrieved 9714 records. Of them, 5755 were duplicates and therefore removed. After reviewing the titles and abstracts of the remaining articles, 3655 studies were excluded because they were case reports (*n* = 2), reviews/meta-analyses (*n* = 202), meeting abstracts (*n* = 1), letters (*n* = 11), protocols (*n* = 49), non-cell or animal studies (*n* = 46), studies without related indicators (*n* = 483), studies with nano-sized materials as drug carriers (*n* = 1116) or studies with irrelevant topics (*n* = 1745). The examination of the full texts further eliminated 74 studies since they were studies with particle size > 100 nm or unknown (*n* = 57), studies with data unavailability (*n* = 4), studies without a control group (*n* = 4), retracted studies (*n* = 1), not peer-reviewed (*n* = 1) or contained indicators that were measured in less than three studies (*n* = 7). Eventually, 230 articles were included for quantitative synthesis (Figure 1; Supplementary File S1).

### 3.2. Study Characteristics and Quality Assessment

The main characteristics of all included studies are summarized in Tables S1 and S2. These studies were published from 2009 to 2025 and conducted in 31 countries, including Argentina (*n* = 1), Austria (*n* = 1), Belgium (*n* = 1), Brazil (*n* = 3), Canada (*n* = 3), China (*n* = 134), Egypt (*n* = 28), France (*n* = 1), Greece (*n* = 1), India (*n* = 7), Indonesia (*n* = 1), Iran (*n* = 3), Italy (*n* = 2), Korea (*n* = 14), Mexico (*n* = 1), Pakistan (*n* = 1), Poland (*n* = 2), Portugal (*n* = 1), Romania (*n* = 2), Saudi Arabia (*n* = 4), Singapore (*n* = 1), Slovakia (*n* = 2), Slovenia (*n* = 1), Spain (*n* = 3), Switzerland (*n* = 1), Thailand (*n* = 1), Tunisia (*n* = 1), Turkey (*n* = 2), UAE (*n* = 1), UK (*n* = 2) and the USA (*n* = 4).

Various nano-sized particles were used for exposure to cells or animals in these studies, such as SiNPs, ZnONPs, AgNPs, TiO_2_NPs (titanium dioxide NPs), FeNPs (iron NPs), PtNPs (platinum NPs), Y_2_O_3_NPs (yttrium oxide NPs), NiNPs (nickel NPs), CuONPs (copper oxide NPs), CeO_2_NPs (ceria NPs), CoNPs (cobalt NPs), Al_2_O_3_NPs (aluminum oxide NPs), PSNPs (polystyrene NPs), CBNPs (carbon black NPs), NDs (nanodiamonds), Ag_2_SeQDs (silver selenide QDs), CdTeQDs (cadmium sulphide QDs), GOs (graphene oxides) and CNTs. Their exposure doses or durations varied between studies.

One hundred and forty-five articles investigated the toxic effects of nano-sized particles on normal or cancer cells isolated from human [e.g., HUVECs, L-02, placental trophoblasts (HTR-8/SVneo), cardiomyocytes (AC16), intestinal epithelial cells (NCM460), proximal tubule epithelial cells (HK-2), embryonic kidney cells (HEK293T), primary villous cytotrophoblasts, gastric epithelial cells (GES-1), bronchial epithelial cells (16HBE, BEAS-2B), umbilical vein endothelial cells (EA.hy926), retinal pigment epithelial cells (ARPE-19), lung fibroblasts (MRC-5), keratinocytes (HaCaT), dermal keratinocytes (HaCaTs), breast carcinoma (MCF-7), hepatocellular carcinoma (HepG2, QGY), colorectal cancer (HT-29, Caco-2), lung adenocarcinoma (A549), neuroblastoma (SH-SY5Y), monocytic leukemia (THP-1), epidermoid skin cancer (A-431), melanoma (A-375), lymphoma (U937), tongue cancer (HNO-97), ovarian carcinoma (SK-OV3), ovarian granulosa (COV434) and mesothelium-derived epithelial (MeT-5A) cells], murine [e.g., mouse testicular Leydig cells (TM3), BDF1 sperm cells, leukemic monocytes/macrophages (RAW264.7), cardiomyocytes (HL-1), spermatocytes (GC-2spd), spermatogonia (GC-1 spg), hippocampal cells (HT22), pre-osteoblasts (MC3T3-E1), mammary epithelial cells (HC11), primary corneal endothelial cells, antral follicles, auditory cells (HEI-OC1), a motor-neuron-like cell line (NSC-34), podocytes, Alpha liver-12 cells (AML-12); rat cardiomyoblasts (H9C2), primary hepatocytes, cortical astrocytes, brain capillary endothelial cells (rBCEC4), vascular smooth muscle cells], porcine (oocytes, coronary artery endothelial cells), bovine (primary intestinal epithelial cells) or fish models [rainbow trout: gill epithelial cells (RTgill-W1); gilthead: seabream neuronal-like stem cells (SaB-1)]. In vivo studies were performed in 107 articles (some had cell types in common with in vitro studies), which used murine (rat, mouse), chicken, fish/shellfish (yellow catfish, zebrafish, turbots, rainbow trout, Nile tilapia, common carp, pearl spot, mussel) or insect (planarians) pre-clinical model organisms and detected related indicators in different tissues (e.g., blood, liver, lung, kidney, spleen, heart, brain, jejunum, testis, placenta, ovarian, cochlea, pancreas, aorta, parotids, gill, bursa of Fabricius, thymus) or the whole body.

mtDNA content was determined by PCR amplification using the DNA template and the primers for mtDNA-encoded genes (e.g., ND1-6, COX1, CYTB, ATP6, 12S rRNA or a non-coding control region D-loop) and nDNA genes (e.g., GAPDH, β-actin, 36B4, Pecam, β2M, 16s rRNA, SLCO2B1/SERPINA1 or Tert) followed by calculation of the mtDNA/nDNA ratio. mRNA expression levels of mtDNA-encoded genes (including ND1,3, COX1-2, CYTB or ATP6) were detected by RT-PCR using the cDNA template and the primers for corresponding genes. Protein expression levels of COX1 and COX2 were analyzed by WB. Expression of mtDNA maintenance genes [including AMPK, phosphorylated (p)-AMPK, p-AMPK/AMPK, SIRT1, PGC-1α, NRF1, NRF2, p-NRF2, TFAM, MFN1, MFN2, OPA1, DRP1, p-DRP1, p-DRP1/DRP-1, FIS1 and MFF] were examined at mRNA and/or protein levels through RT-PCR, WB, IHC, ICC, IF or ELISA methods.

The Toxrtool score of all studies was equal or more than 15, indicating all of them were high-methodological-quality studies (Tables S3 and S4).

### 3.3. Meta-Analysis for In Vitro Studies

**Figure 2:**
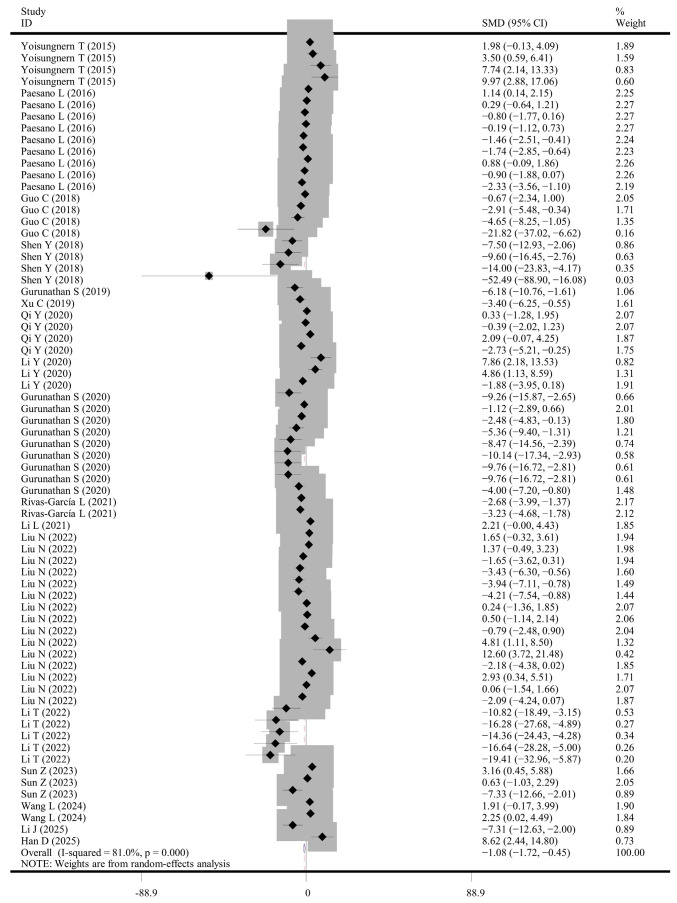
Forest plots assessing the effect of exposure to nano-sized particles on mtDNA content in cells. mtDNA, mitochondrial DNA; SMD, standardized mean difference; CI, confidence interval. Squares (grey): effect sizes for individual study; diamonds: pooled effect sizes; the center denotes the point estimate, while the horizontal line represents the 95% CI; vertical dashed line: the overall mean effect size across all included studies.

**Figure 3:**
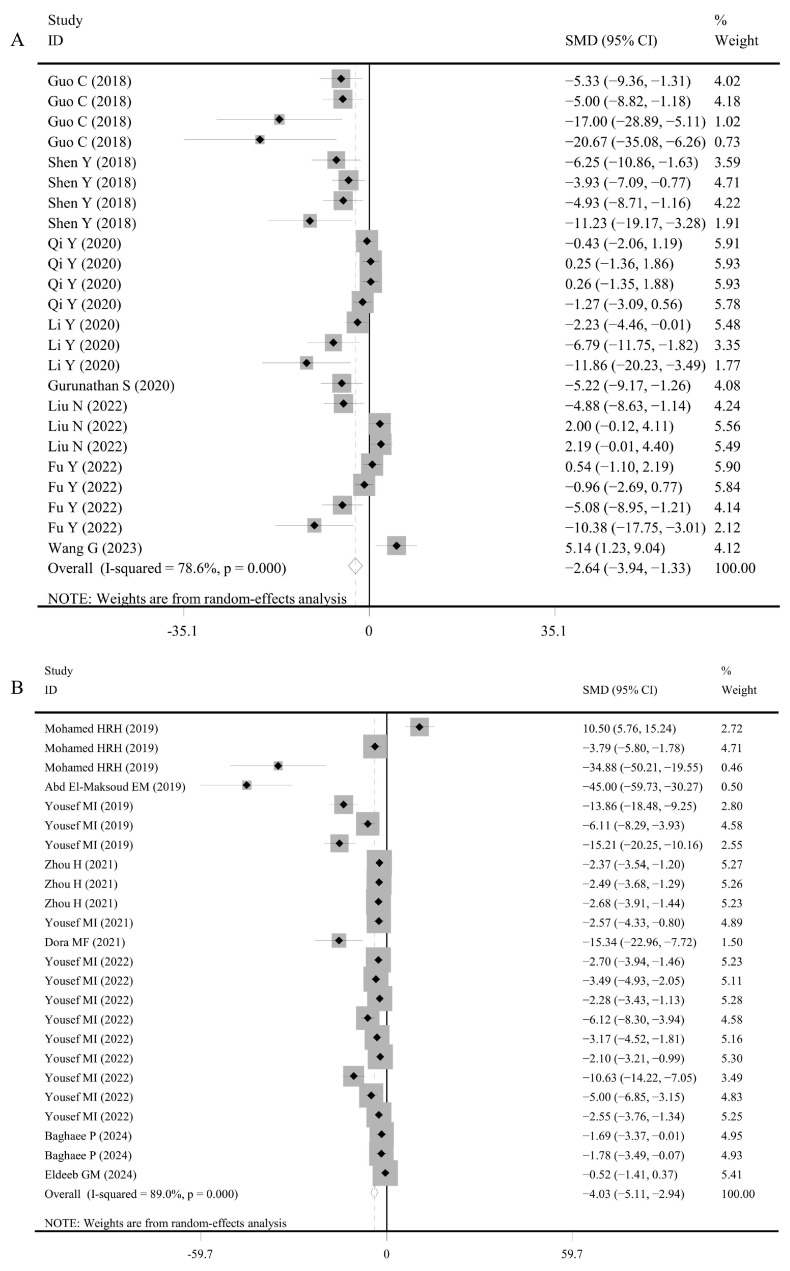
Forest plots assessing the effect of exposure to nano-sized particles on the expression levels of TFAM: (**A**) analysis of in vitro studies; (**B**) analysis of in vivo studies. TFAM, mitochondrial transcription factor A; SMD, standardized mean difference; CI, confidence interval. Squares (grey): effect sizes for individual study; diamonds: pooled effect sizes; the center denotes the point estimate, while the horizontal line represents the 95% CI; vertical dashed line: the overall mean effect size across all included studies.

**Figure 4:**
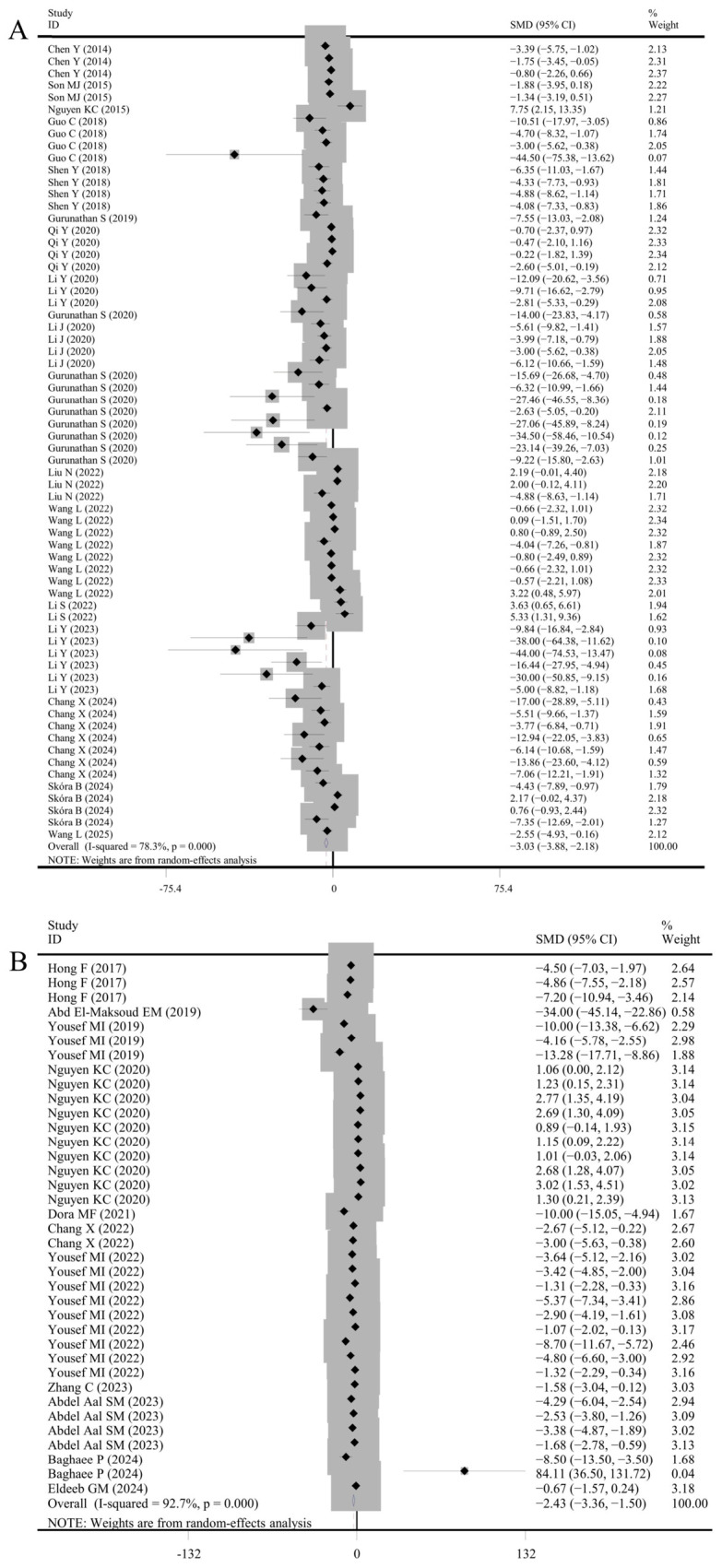
Forest plots assessing the effect of exposure to nano-sized particles on the expression levels of PGC-1α: (**A**) analysis of in vitro studies; (**B**) analysis of in vivo studies. PGC-1α, peroxisome proliferator-activated receptor-γ coactivator 1alpha; SMD, standardized mean difference; CI, confidence interval. Squares (grey): effect sizes for individual study; diamonds: pooled effect sizes; the center denotes the point estimate, while the horizontal line represents the 95% CI; vertical dashed line: the overall mean effect size across all included studies.

The data for 25 indicators (Table 1) were extracted from 145 in vitro studies to assess the toxic effects of nano-sized particle exposure on mtDNA. Meta-analysis was performed on these indicators’ values under a random-effects model. The results showed that the mtDNA content (SMD = −1.08; *P*_ES_ = 0.001) (Figure 2), the expression levels of mtDNA-encoded genes ND1 (SMD = −1.30; *P*_ES_ = 0.033), COX1 (SMD = −4.01; *P*_ES_ < 0.001), COX2 (SMD = −2.57; *P*_ES_ = 0.019), CYTB (SMD = −3.36; *P*_ES_ = 0.002), and ATP6 (SMD = −5.17; *P*_ES_ < 0.001), mitochondrial biogenesis-related genes TFAM (SMD = −2.64; *P*_ES_ < 0.001) (Figure 3A), PGC-1α (SMD = −3.03; *P*_ES_ < 0.001) (Figure 4A), and SIRT1 (SMD = −2.54; *P*_ES_ < 0.001) and mitochondrial fusion-related genes MFN1 (SMD = −1.26; *P*_ES_ < 0.001), MFN2 (SMD = −0.96; *P*_ES_ = 0.002) and OPA1 (SMD = −1.91; *P*_ES_ < 0.001) were significantly reduced, while the expression levels of NRF2 (total: SMD = 1.69; nuclear: SMD = 1.62; both *P*_ES_ < 0.001), p-AMPK (SMD = 5.54; *P*_ES_ < 0.001), p-AMPK/AMPK (SMD = 5.52; *P*_ES_ < 0.001) and mitochondrial fission-related genes DRP1 (SMD = 1.73; *P*_ES_ < 0.001) and p-DRP1 (SMD = 2.67; *P*_ES_ < 0.001) were significantly increased in cells exposed to nano-sized particles when compared to the non-exposed control groups (Table 1). No significant differences in the expression levels of ND3, NRF1, cytosolic NRF2, p-NRF2, p-DRP1/DRP1, FIS1 and MFF were detected between nano-sized particle-exposed and non-exposed cells *(P*_ES_ > 0.05; Table 1).

### 3.4. Subgroup and Meta-Regression Analyses for In Vitro Studies

Due to the presence of statistical heterogeneity among analyzed studies (Table 1), subgroup and meta-regression analyses were conducted for indicators included in at least ten articles to explore potential sources of heterogeneity. As shown in Table S5, the heterogeneity seemed to be eliminated and significant changes with similar trends to the overall meta-analysis were still present when subgroup analysis was performed for indicators of mtDNA content [stratified by particle type (e.g., CBNPs: SMD = −9.65), cell type (e.g., placenta cell HTR-8/SVneo: SMD = −11.65)], MFN2 [stratified by particle type (e.g., CBNPs: SMD = −1.79), cell type (e.g., cardiomyocyte HL-1, AC16: SMD = −2.81)], OPA1 [stratified by cell type (e.g., cardiomyocyte HL-1, AC16: SMD = −1.52)], DRP1 [stratified by cell type (e.g., testis cell GC-1, TM4: SMD = 8.39; blood immune cell RAW264.7, THP-1: SMD = 4.98)], NRF2 (total) [stratified by cell type (e.g., neuron SH-SY5Y, BV2, SaB-1: SMD = 3.91)] and nuclear NRF2 [stratified by particle type (e.g., SiNPs: SMD = 6.08)] (all *P*_ES_ < 0.001), indicating these subgroup factors may be potential sources of heterogeneity for these variables. Meta-regression analysis revealed country was the cause of heterogeneity for DRP1 (Table S7), which may be indirectly reflected by the fact that the expression level of DRP1 was only significantly elevated by nano-sized particles in studies performed in Asian countries (SMD = 1.99; *P*_ES_ < 0.001), but not in non-Asian countries (*P*_ES_ = 0.347). Furthermore, country and cell type were confirmed as contributors to the heterogeneity of NRF2 during meta-regression analysis. The heterogeneity of other variables is unclear since the heterogeneity could not be eliminated according to our subgroup or meta-regression analyses. Although a fixed-effects model was applied in some subgroups (Table S7), related data were collected from the same study; thus, we did not address them and more studies should be included to ascertain their influences.

### 3.5. Meta-Analysis for In Vivo Studies

A total of 13 indicators (Table 2) were available from the 107 in vivo studies that evaluated the toxic effects of nano-sized particle exposure on mtDNA. Under the random-effects model, the pooled analysis demonstrated that, compared with the non-exposed controls, exposure of animal models to nano-sized particles induced a significant decrease in the expression levels of TFAM (SMD = −4.03; *P*_ES_ < 0.001) (Figure 3B), PGC-1α (SMD = −2.43; *P*_ES_ < 0.001) (Figure 4B), NRF2 (SMD = −0.81; *P*_ES_ = 0.006), SIRT1 (SMD = −1.88; *P*_ES_ = 0.034), and MFN2 (SMD = −1.80; *P*_ES_ < 0.001) and a significant increase in the expression levels of DRP1 (SMD = 2.89; *P*_ES_ < 0.001), p-DRP1 (SMD = 1.71; *P*_ES_ = 0.030) and FIS1 (SMD = 1.71; *P*_ES_ < 0.001) (Table 2). The mtDNA content and the expression levels of nuclear NRF2, MFN1 and OPA1 were found not to be significantly changed after the animals were exposed to nano-sized particles *(P*_ES_ > 0.05; Table 2).

### 3.6. Subgroup and Meta-Regression Analyses for In Vivo Studies

Subgroup and meta-regression analyses were conducted to explore potential sources of heterogeneity since there was evidence of significant heterogeneity in the analysis of in vivo studies. Unfortunately, the heterogeneity still existed in the subgroup analysis for most variables (Table S6). Even if the heterogeneity seemed to be removed in some subgroups, this conclusion needs further confirmation because of the very small sample size (≤two datasets) or small number of included articles (one study). Meta-regression analysis also did not identify the source of heterogeneity for those four indicators analyzed in more than ten articles (Table S8). However, in line with the overall meta-analysis results, the expression levels of PGC-1α, NRF2, MFN2 and FIS1 were shown to be significantly up-regulated or down-regulated in the lung, brain, heart and testis tissues of animals exposed to different nano-sized particles (e.g., AgNPs, PSNPs). Interestingly, the expression of MFN2 was significantly reduced by nano-sized particle exposure at both mRNA and protein levels, while the expression of PGC-1α and DRP1/NRF2/FIS1 was only changed at mRNA or protein level, respectively. These findings suggest MFN2 may be a particularly important target for explaining nano-sized particle-induced toxicity in animals.

### 3.7. PB and Sensitivity Analysis

**Figure 5:**
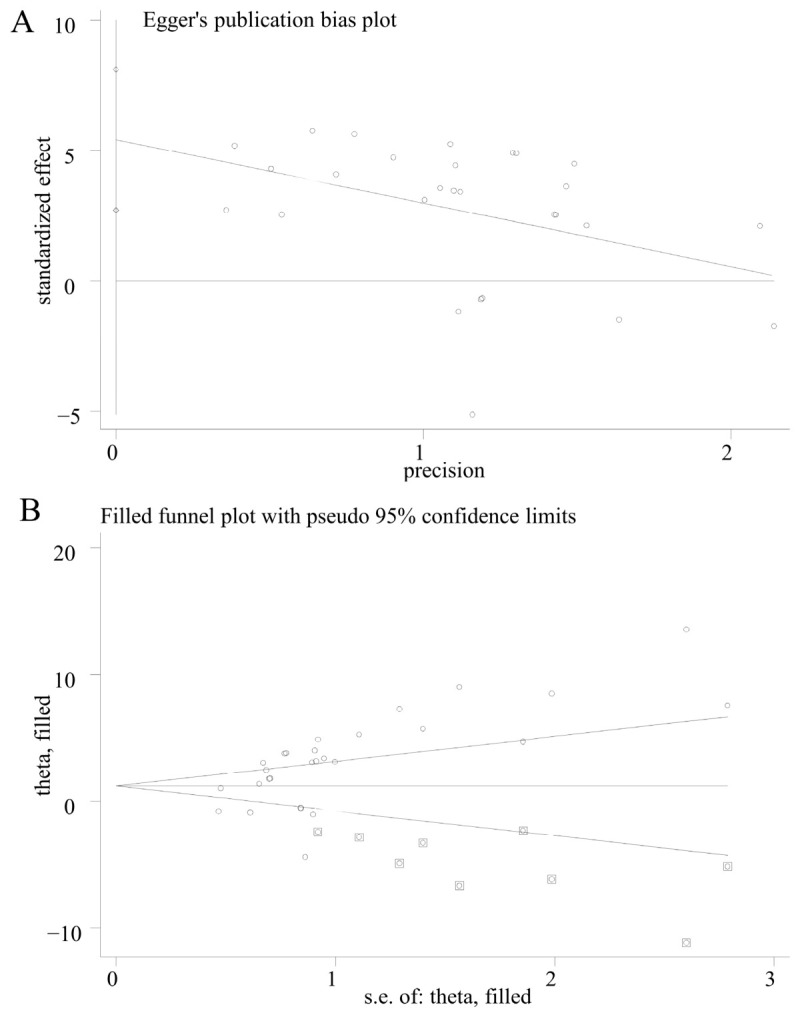
Egger’s linear regression test (**A**) and the filled funnel plot (**B**) for analysis of DRP1 in animals exposed to nano-sized particles. DRP1, dynamin-related protein 1. Squares: possible missing studies; circles: each included study; the asymmetry on both sides of funnel plot (formed by line) suggested the existence of publication bias.

Egger’s linear regression test results are summarized in Table 1 and Table 2. Significant bias was identified in the analysis of most outcomes (Figure 5A). A trim-and-fill correction analysis was then performed for variables with a significant PB (Figure 5B). Consequently, no changes in the ES were found for analysis of mtDNA content and the expression of ND1, COX1, COX2, CYTB, ATP6, OPA1, NRF1, SIRT1, cytosolic NRF2 (in vitro), TFAM, PGC-1α, MFN2 (both in vitro and in vivo) or total NRF2 (in vivo). Similarly to pre-correction, significant results were still observed for DRP1 (in vitro: SMD = 3.31, 95%CI = 2.87–3.82, *P*_ES_ < 0.001; in vivo: SMD = 3.27, 95%CI = 2.44–4.36, *P*_ES_ < 0.001), p-DRP1 (in vitro: SMD = 4.74, 95%CI = 3.12– 7.19, *P*_ES_ < 0.001), FIS1 (in vitro: SMD = 1.20, 95%CI = 0.94–1.52, *P*_ES_ < 0.001), total NRF2 (in vitro: SMD = 2.23, 95%CI = 1.94–2.55, *P*_ES_ < 0.001), nuclear NRF2 (in vitro: SMD = 1.92, 95%CI = 1.45–2.54, *P*_ES_ < 0.001), p-AMPK (in vitro: SMD = 2.67, 95%CI = 1.67–3.67, *P*_ES_ < 0.001) and p-AMPK/AMPK (in vitro: SMD = 2.88, 95%CI = 2.06–3.69, *P*_ES_ < 0.001) under a fixed-effects model, whereas a non-significant result was detected for p-DRP1/DRP1 (in vitro: SMD = −0.24, 95%CI = −1.08–0.61, *P*_ES_ = 0.582), although the ES was slightly altered after correction.

**Figure 6:**
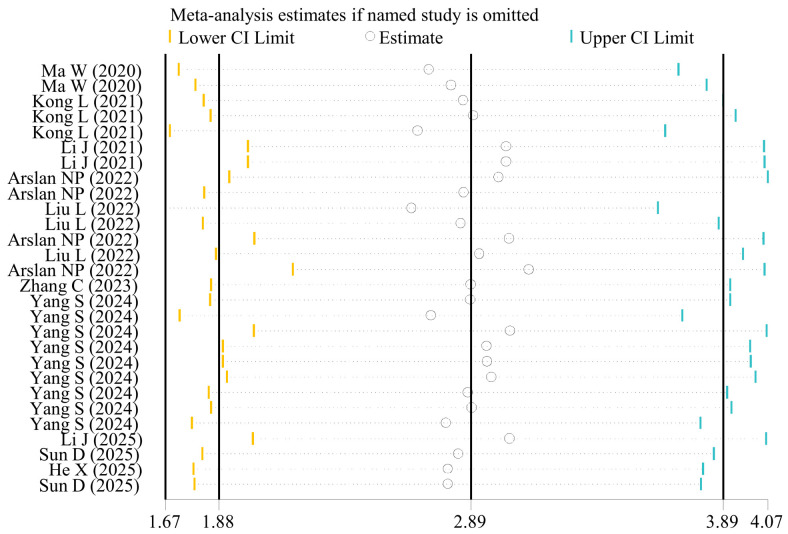
Sensitivity analysis for DRP1 in animals exposed to nano-sized particles. DRP1, dynamin-related protein 1; CI, confidence interval. Every circle indicates the pooled result. The two ends of every broken line represent the lower (yellow) and upper (green) 95% CI.

Sensitivity analyses showed no significant impact on the overall findings by individually removing each study one by one. This result provided support for the stability and reliability of our results (Figure 6).

## 4. Discussion

Recently, there have been two studies that use a meta-analysis approach to explore the influence of exposure to environmental pollutants on the mtDNA content in humans [6,7]. These studies mainly focused on heavy metals in the bulk state and particulate matter with an aerodynamic diameter < 2.5 or 10 μm [6,7]. Their pooled results indicated no significant changes in mtDNA content in response to the above environmental pollutants with relatively large particle sizes. Considering the higher toxicological effects of nano-sized particles than their bulk counterparts (as evidenced by a significant reduction in cell viability and an increase in ROS generation, nDNA damage, cell apoptosis and organ dysfunction) [37,38,39], mtDNA was suggested to be also more vulnerable to damages induced by nano-sized particles. This highlighted the necessity of independently evaluating the changes in the mtDNA content after exposure to nano-sized particles. To the best of our knowledge, this was the first meta-analysis to comprehensively examine the effects of nano-sized particle exposure on the mtDNA content. A total of 19 (69 datasets) in vitro and seven (nine datasets) in vivo studies reported mtDNA results and were subsequently pooled. Different from the studies on large airborne particles [6,7], our meta-analysis showed the mtDNA content was significantly reduced in cells exposed to nano-sized particles. These findings demonstrated a size-dependent toxic effect of environmental particles on mtDNA.

Additionally, some studies used RT-PCR or WB assay methods to determine the expression levels of mtDNA-encoded genes. Although mtDNA expression was not directly related to the mtDNA content, it may indirectly reflect mtDNA integrity and function [40,41]. It was reported that exposure to ultraviolet C radiation induced persistent mtDNA damage, which was accompanied by significantly reduced mRNA levels of mtDNA-encoded genes and ATP production at early time points (whereas alteration in mtDNA content was only observed at later time points) [42]. mtDNA point mutations and deletions were identified to lead to down-regulated transcription and translation of mtDNA-encoded genes [43]. Therefore, a meta-analysis was also performed for the expression levels of mtDNA-encoded genes. Similarly to mtDNA content, the expression levels of mtDNA-encoded genes ND1, COX1, COX2, CYTB, and ATP6 were found to be significantly inhibited in cells upon exposure to nano-sized particles. The non-significant results for ND3 may be, on one hand, associated with the small sample size for the meta-analysis or, on the other hand, a consequence of rare mtDNA deletion mutations in the coding regions for this gene due to oxidant exposure [44]. Significant reduction in the expression of these five mtDNA-encoded genes may also explain the impairments of the mitochondrial electron transport chain and the resultant mitochondrial dysfunction (as indicated by lower levels of ATP production, proton leakage, basal and maximal respiration, spare respiratory capacity and non-mitochondrial oxygen consumption) caused by nano-sized particles [45].

Accumulating evidence suggested the replication, transcription and translation of mtDNA may be regulated by the AMPK/SIRT1-PGC-1α-NRF1/2-TFAM signaling pathway [10,11]. Small interfering RNA (siRNA)- or short hairpin RNA (shRNA)-mediated knockdown (KD) of TFAM was reported to reduce mtDNA content [46], mRNA levels of mtDNA-encoded ND1 gene [47], COX activity [48] and oxidative respiration efficiency [49], while transfection of overexpression plasmids significantly enhanced the mtDNA content [50] and reversed mitochondrial OXPHOS defects [51]. PGC-1α-KD embryos were revealed to exhibit significantly lower levels of mtDNA content and down-regulated expression of mitochondrial genes (ND1, ND3, ND5, ATP8, COX1, COX2 and CYTB), TFAM and NRF1 [52]. PGC-1α activator (ZLN005) administration could alleviate mitochondrial damage in cardiomyocytes by increasing mtDNA and mitochondrial ATP content [53]. The addition of an AMPK inhibitor, adenine 9-β-d-arabinofuranoside, was demonstrated to attenuate the increase in SIRT1, PGC-1α, NRF-1, TFAM and mitochondrial complexes III/V [54]. Treatment with a SIRT1 inhibitor, EX-527, was shown to cause a marked reduction in mRNA expression of PGC-1α, NRF1, TFAM, mtDNA content and ATP production [55]. Dong et al. identified that impairment of mitochondrial biogenesis induced by Aβ25-35 was associated with suppressed phosphorylation of AMPK and SIRT1 and increased acetylation of PGC-1α, as well as decreased levels of mtDNA content and expression of mitochondrial genes (PGC-1α, NRF1, NRF2 and TFAM) [56]. Vitamin C exerted protective roles against oxidative stress by up-regulation of PGC-1α protein, which subsequently promoted the expression of downstream proteins NRF2 and TFAM, and restored mitochondrial energy production [57]. Therefore, theoretically, the expression levels of AMPK, SIRT1, PGC-1α, NRF1/2 and TFAM may be significantly reduced by nano-sized particles. This hypothesis was confirmed for SIRT1, PGC-1α and TFAM after our meta-analysis of in vitro and in vivo studies. Also, nano-sized particle exposure had been confirmed to trigger damage to mtDNA and indicators associated with mitochondrial function (e.g., collapsed mitochondrial membrane potential, impaired ATP synthesis, respiratory capacity and ATPase activities) simultaneous with the inactivation of the SIRT1-PGC-1α-TFAM signaling pathway [28,58]. However, inconsistent results were identified for NRF2 following meta-analysis of in vitro (an increase) and in vivo studies (a decrease). These different conclusions may be associated with the following reasons: (1) more nano-sized particles (e.g., NiNPs, PSNPs, QDs) that significantly decreased the level of NRF2 were included in in vivo studies, which led to the final down-regulated trend (Tables S5 and S6); (2) NRF2 played multi-functional roles, including having a protective response against oxidant stress for cells exposed to nano-sized particles [59,60]—accordingly, NRF2 was up-regulated. The levels of p-AMPK and p-AMPK/AMPK all proved to be significantly elevated by nano-sized particles. These results, that seemed not to be in line with our expected findings, may also be attributed to the fact that AMPK is involved in multiple signaling pathways (AMPK/mTOR; protective autophagy against cell death induced by nano-sized particles) [61,62]. NRF1 levels were not found to be significantly altered by nano-sized particles in our meta-analysis. This result may be due to the small number of studies included and further validation is necessary in the future.

The mitochondrial dynamics (including mitochondrial fusion and fission which are driven by biomarkers of MFN1/MFN2/OPA1 and DRP1/FIS1/MFF, respectively) are increasingly recognized as critical players in controlling mtDNA levels and mitochondrial function [63]. Combined deletion of MFN1/MFN2 in β-cells was reported to reduce mtDNA content, impair mitochondrial structure and decrease respiratory function, while simultaneous TFAM overexpression reversed MFN1/2-deficiency-induced reduction in mtDNA content [64]. In mice with heterozygous mutation of OPA1, mtDNA content was found to be significantly reduced, which was accompanied by lowered COX activity, disrupted mitochondrial organization and depressed oxygen consumption [65]. Overexpression of OPA1 can be effective in alleviating mtDNA loss, enhancing OXPHOS activities and protecting the kidney from damage in mtDNA-depleted mice [66]. A transgenic mouse line that overexpressed Drp1 specifically in skeletal muscle was observed to have a drastic impairment in mtDNA quantity and the mitochondrial network [67]. Down-regulation of DRP1 using its specific inhibitor P110 activated the PGC-1α/TFAM pathway and promoted mitochondrial biogenesis and function [68]. In line with these studies, our meta-analysis found the expression levels of MFN1/MFN2/OPA1 were significantly suppressed, while DRP1 was significantly increased after exposure to nano-sized particles. FIS1 was only revealed to be significantly changed in meta-analysis of in vivo studies, but the number of included articles was less and, thus, the conclusion remains undetermined.

The present study has certain limitations. Firstly, subgroup meta-analyses identified some nano-sized particles (e.g., SiNPs, NiNPs, CBNPs) exert more strong toxic effects, and specific cells [e.g., reproductive cells (HTR-8/SVneo, GC-1 or TM4), heart cells (HL-1, AC16)] or animal organs (lung, heart, testis) that may possess more energy requirements and are highly vulnerable to oxidative stress [69,70] were particularly influenced. However, a relatively small number of studies was included for these variables and the conclusions obtained for them may be inconclusive. Comparisons between different nano-sized particles or cells should be confirmed by designing more comparative studies. Secondly, most of the publications were studies performed in Asian countries. Whether the related indicators were significantly changed by nano-sized particles needs further investigation by non-Asian scholars. Thirdly, this meta-analysis of in vitro and in vivo studies indicated mtDNA content and the expression of mtDNA maintenance genes may represent potential biomarkers for monitoring oxidative injury in occupational workers alongside other oxidative DNA damage biomarkers (e.g., 8-OHdG) [71,72]; however, more clinical research is required to confirm this transformation [73,74]. Fourthly, few or no studies recorded the expression of other mtDNA maintenance genes (e.g., POLG [75], Twinkle, RNaseH1, mitochondrial genome maintenance exonuclease 1). Fifthly, potential deviations may be present between our data extracted from figures by Graph Digitizer and the raw data. Sixthly, the findings should be interpreted with caution because of the strong or moderate evidence of heterogeneity. Also, the source of some heterogeneity was unable to be determined by our subgroup or meta-regression analysis. Seventhly, more specific upstream mechanisms (beyond AMPK and NRF2 being involved in multiple signaling pathways) for mtDNA maintenance should be explored in future research.

## 5. Conclusions

This comprehensive meta-analysis demonstrates exposure of nano-sized particles to cells or animal tissues leads to significant reductions in mtDNA content and alterations in the expression pattern of mtDNA-encoded genes (ND1, COX1, COX2, CYTB and ATP6; down-regulated) and major regulatory genes that mediate the maintenance of mtDNA (biogenesis: SIRT1/PGC-1α/TFAM, down-regulated; fusion: MFN1/MFN2/OPA1, down-regulated; fission: DRP1/FIS1, up-regulated). Our findings may provide potential biomarkers for monitoring oxidative injury in occupational workers exposed to nano-sized particles and molecular targets for developing strategies to mitigate harmful effects, although additional pre-clinical and clinical research should be further performed to confirm this hypothesis.

## Figures and Tables

**Table 1:** Meta-analysis of in vitro studies.

Variable	No.	SMD	95%CI	*P*_ES_-Value	*I* ^2^	*P*_H_-Value	Model	*P*_PB_-Value
mtDNA content	19(69)	−1.08	−1.72, −0.45	**0.001**	81.0	<0.001	R	0.008
Expression of ND1	5(14)	−1.30	−2.50, −0.10	**0.033**	92.9	<0.001	R	0.010
Expression of ND3	6(8)	0.13	−7.92, 8.19	0.974	88.4	<0.001	R	0.775
Expression of COX1	5(15)	−4.01	−6.17, −1.85	**<0.001**	80.7	<0.001	R	<0.001
Expression of COX2	4(12)	−2.57	−4.72, −0.43	**0.019**	79.4	<0.001	R	0.002
Expression of CYTB	4(10)	−3.36	−5.53, −1.20	**0.002**	76.5	<0.001	R	<0.001
Expression of ATP6	4(13)	−5.17	−7.78, −2.57	**<0.001**	81.9	<0.001	R	<0.001
Expression of TFAM	8(24)	−2.64	−3.94, −1.33	**<0.001**	78.6	<0.001	R	<0.001
Expression of PGC-1α	19(66)	−3.03	−3.88, −2.18	**<0.001**	78.3	<0.001	R	<0.001
Expression of NRF1	6(22)	−0.93	−2.28, 0.42	0.177	79.2	<0.001	R	0.005
Expression of NRF2 (total)	63(248)	1.69	1.32, 2.05	**<0.001**	80.4	<0.001	R	<0.001
Expression of NRF2 (nuclear)	18(60)	1.62	0.99, 2.25	**<0.001**	75.3	<0.001	R	<0.001
Expression of NRF2 (cytosolic)	9(17)	−0.31	−1.19, 0.57	0.494	66.8	<0.001	R	0.001
Expression of p-NRF2	4(8)	0.47	−3.46, 4.40	0.814	86.1	<0.001	R	0.754
Expression of p-AMPK	4(11)	5.54	3.47, 7.61	**<0.001**	62.2	0.003	R	<0.001
p-AMPK/AMPK	8(20)	5.52	3.63, 7.40	**<0.001**	71.1	<0.001	R	<0.001
SIRT1	9(50)	−2.54	−3.34, −1.74	**<0.001**	75.9	<0.001	R	<0.001
Expression of MFN1	24(87)	−1.26	−1.87, −0.64	**<0.001**	88.4	<0.001	R	0.139
Expression of MFN2	25(102)	−0.96	−1.58, −0.35	**0.002**	90.0	<0.001	R	<0.001
Expression of OPA1	25(101)	−1.91	−2.44, −1.39	**<0.001**	87.9	<0.001	R	<0.001
Expression of DRP1	32(125)	1.73	1.29, 2.18	**<0.001**	82.4	<0.001	R	0.014
Expression of p-DRP1	12(49)	2.67	1.67, 3.67	**<0.001**	77.0	<0.001	R	0.022
p-DRP1/DRP1	3(10)	1.90	−0.54, 4.34	0.126	82.6	<0.001	R	0.023
Expression of FIS1	23(80)	0.51	−0.07, 1.09	0.084	77.8	<0.001	R	0.013
Expression of MFF	4(16)	−0.41	−2.12, 1.29	0.637	79.7	<0.001	R	0.452

mtDNA, mitochondrial DNA; ND1, NADH dehydrogenase subunit 1; ND3, NADH dehydrogenase subunit 3; COX1, cytochrome c oxidase subunit 1; COX2, cytochrome c oxidase subunit 2; CYTB, cytochrome b; ATPase 6, ATP synthase F0 subunit 6; TFAM, mitochondrial transcription factor A; PGC-1α, peroxisome proliferator-activated receptor-γ coactivator 1alpha; NRF1, nuclear respiratory factor-1; NRF2, nuclear respiratory factor-2; p, phosphorylated; AMPK, AMP-activated protein kinase; SIRT1, sirtuin 1; MFN1, mitochondrial fusion protein 1; MFN2, mitochondrial fusion protein 2; OPA1, optic atrophy protein 1; DRP1, dynamin-related protein 1; FIS1, mitochondrial fission protein 1; MFF, mitochondrial fission factor; SMD, standardized mean difference; CI, confidence interval; F, fixed-effects; R, random-effects; *P*_H_-value, significance for heterogeneity; *P*_ES_-value, significance for effect size; *P*_PB_-value, significance for publication bias; No., number of studies (datasets). Bold indicates the indicators with significant results.

**Table 2:** Meta-analysis of in vivo studies.

Variable	No.	SMD	95%CI	*P*_ES_-Value	*I* ^2^	*P*_H_-Value	Model	*P*_PB_-Value
mtDNA content	7(9)	0.17	−2.20, 2.54	0.886	89.6	<0.001	R	0.983
Expression of TFAM	9(24)	−4.03	−5.12, −2.94	**<0.001**	89.0	<0.001	R	<0.001
Expression of PGC-1α	11(37)	−2.43	−3.36, −1.50	**<0.001**	92.7	<0.001	R	0.001
Expression of NRF2 (total)	69(185)	−0.81	−1.39, −0.24	**0.006**	96.8	<0.001	R	0.002
Expression of NRF2 (nuclear)	3(9)	1.09	−1.40, 3.57	0.392	91.2	<0.001	R	0.410
Expression of AMPK	3(6)	3.32	0.67, 5.96	**0.014**	88.9	<0.001	R	0.302
Expression of SIRT1	8(11)	−1.88	−3.61, −0.14	**0.034**	87.1	<0.001	R	0.157
Expression of MFN1	7(18)	−0.50	−1.19, 0.18	0.151	77.8	<0.001	R	0.588
Expression of MFN2	10(23)	−1.80	−2.70, −0.90	**<0.001**	87.5	<0.001	R	0.003
Expression of OPA1	8(11)	−1.47	−3.25, 0.32	0.107	89.7	<0.001	R	0.520
Expression of DRP1	11(28)	2.89	1.88, 3.89	**<0.001**	90.0	<0.001	R	<0.001
Expression of p-DRP1	3(5)	1.71	0.16, 3.26	**0.030**	75.4	0.003	R	0.579
Expression of FIS1	11(15)	1.71	0.76, 2.67	**<0.001**	75.2	<0.001	R	0.083

mtDNA, mitochondrial DNA; TFAM, mitochondrial transcription factor A; PGC-1α, peroxisome proliferator-activated receptor-γ coactivator 1alpha; NRF2, nuclear respiratory factor-2; AMPK, AMP-activated protein kinase; SIRT1, sirtuin 1; MFN1, mitochondrial fusion protein 1; MFN2, mitochondrial fusion protein 2; OPA1, optic atrophy protein 1; DRP1, dynamin-related protein 1; p, phosphorylated; FIS1, mitochondrial fission protein 1; SMD, standardized mean difference; CI, confidence interval; F, fixed-effects; R, random-effects; *P*_H_-value, significance for heterogeneity; *P*_ES_-value, significance for effect size; *P*_PB_-value, significance for publication bias; No., number of studies (datasets). Bold indicates the indicators with significant results.

## Data Availability

The data used for meta-analysis in the study can be seen in the included articles and Table S2.

## Supplementary Materials

The following supporting information can be downloaded at: https://www.mdpi.com/article/10.3390/antiox15070848/s1, Table S1: Characteristics of included articles; Table S2: Extracted data for each indicator; Table S3: Quality assessments for in vitro studies; Table S4: Quality assessments for in vivo studies; Table S5: Subgroup meta-analysis for in vitro studies; Table S6: Subgroup meta-analysis for in vivo studies; Table S7: Meta-regression analysis for in vitro studies; Table S8: Meta-regression analysis for in vivo studies.
