# Supplementary material for: Toxicity Evaluation of Nano-Sized Particles by Analysis of mtDNA Content and Expression Levels of Genes Required for mtDNA Maintenance: A Meta-Analysis of Pre-Clinical Studies
Source: Antioxidants (Basel). 2026 Jul 4;15(7):848. doi: 10.3390/antiox15070848 (PMC13405982; doi:10.3390/antiox15070848)
Supplement: Supplementary file 1 [file antioxidants-15-00848-s001.zip › Supplementary file S1.pdf]

Included articles for meta-analysis:

1. Sun Z, Wen Y, Zhang F, Fu Z, Yuan Y, Kuang H, Kuang X, Huang J, Zheng L, Zhang D. Exposure to nanoplastics induces mitochondrial impairment and cytomembrane destruction in Leydig cells. Ecotoxicol Environ Saf. 2023;255:114796.
2. Qi Y, Ma R, Li X, Lv S, Liu X, Abulikemu A, Zhao X, Li Y, Guo C, Sun Z. Disturbed mitochondrial quality control involved in hepatocytotoxicity induced by silica nanoparticles. Nanoscale. 2020;12(24):13034-13045.
3. Li Y, Li F, Zhang L, Zhang C, Peng H, Lan F, Peng S, Liu C, Guo J. Zinc Oxide Nanoparticles Induce Mitochondrial Biogenesis Impairment and Cardiac Dysfunction in Human iPSC-Derived Cardiomyocytes. Int J Nanomedicine. 2020;15:2669-2683.
4. Guo C, Wang J, Jing L, Ma R, Liu X, Gao L, Cao L, Duan J, Zhou X, Li Y, Sun Z. Mitochondrial dysfunction, perturbations of mitochondrial dynamics and biogenesis involved in endothelial injury induced by silica nanoparticles. Environ Pollut. 2018;236:926-936.
5. Yoisungnern T, Choi YJ, Han JW, Kang MH, Das J, Gurunathan S, Kwon DN, Cho SG, Park C, Chang WK, Chang BS, Parnpai R, Kim JH. Internalization of silver nanoparticles into mouse spermatozoa results in poor fertilization and compromised embryo development. Sci Rep. 2015;5:11170.
6. Paesano L, Perotti A, Buschini A, Carubbi C, Marmiroli M, Maestri E, Iannotta S, Marmiroli N. Markers for toxicity to HepG2 exposed to cadmium sulphide quantum dots; damage to mitochondria. Toxicology. 2016;374:18-28.
7. Mohamed HRH, Behira LST, Diab A. Estimation of genomic and mitochondrial DNA integrity in the renal tissue of mice administered with acrylamide and titanium dioxide nanoparticles. Sci Rep. 2023;13(1):13523.
8. Rivas-García L, Quiles JL, Varela-López A, Giampieri F, Battino M, Bettmer J, Montes-Bayón M, Llopis J, Sánchez-González C. Ultra-Small Iron Nanoparticles Target Mitochondria Inducing Autophagy, Acting on Mitochondrial DNA and Reducing Respiration. Pharmaceutics. 2021;13(1):90.
9. Mohamed HRH. Estimation of genomic instability and mitochondrial DNA damage induction by acute oral administration of calcium hydroxide normal- and nano- particles in mice. Toxicol Lett. 2019;304:1-12.
10. Gurunathan S, Jeyaraj M, Kang MH, Kim JH. Melatonin Enhances Palladium-Nanoparticle-Induced Cytotoxicity and Apoptosis in Human Lung Epithelial Adenocarcinoma Cells A549 and H1229. Antioxidants (Basel). 2020;9(4):357.
11. Li L, Bi Z, Hu Y, Sun L, Song Y, Chen S, Mo F, Yang J, Wei Y, Wei X. Silver nanoparticles and silver ions cause inflammatory response through induction of cell necrosis and the release of mitochondria in vivo and in vitro. Cell Biol Toxicol. 2021;37(2):177-191.
12. Xu C, Liu Q, Liu H, Zhang C, Shao W, Gu A. Toxicological assessment of multi-walled carbon nanotubes in vitro: potential mitochondria effects on male reproductive cells. Oncotarget. 2016;7(26):39270-39278.
13. Anand AS, Jain K, Chauhan A, Prasad DN, Kohli E. Zinc oxide nanoparticles trigger dysfunction of mitochondrial respiratory complexes and repair dynamics in human alveolar cells. Toxicol Ind Health. 2023;39(3):127-137.
14. Son MJ, Kim WK, Kwak M, Oh KJ, Chang WS, Min JK, Lee SC, Song NW, Bae KH. Silica nanoparticles inhibit brown adipocyte differentiation via regulation of p38 phosphorylation.

Nanotechnology. 2015;26(43):435101.
15. Gurunathan S, Jeyaraj M, Kang MH, Kim JH. Mitochondrial Peptide Humanin Protects Silver Nanoparticles-Induced Neurotoxicity in Human Neuroblastoma Cancer Cells (SH-SY5Y). Int J Mol Sci. 2019;20(18):4439.
16. Abd El-Maksoud EM, Lebda MA, Hashem AE, Taha NM, Kamel MA. Ginkgo biloba mitigates silver nanoparticles-induced hepatotoxicity in Wistar rats via improvement of mitochondrial biogenesis and antioxidant status. Environ Sci Pollut Res Int. 2019;26(25):25844-25854.
17. Xu C, Qiao L, Ma L, Guo Y, Dou X, Yan S, Zhang B, Roman A. Biogenic selenium nanoparticles synthesized by Lactobacillus casei ATCC 393 alleviate intestinal epithelial barrier dysfunction caused by oxidative stress via Nrf2 signaling-mediated mitochondrial pathway. Int J Nanomedicine. 2019;14:4491-4502.
18. Zhao T, Zheng H, Xu JJ, Pantopoulos K, Xu YC, Liu LL, Lei XJ, Kotzamanis YP, Luo Z. MnO_2 nanoparticles trigger hepatic lipotoxicity and mitophagy via mtROS-dependent Hsf1Ser326 phosphorylation. Free Radic Biol Med. 2024;210:390-405.
19. Liu N, Liang Y, Wei T, Zou L, Bai C, Huang X, Wu T, Xue Y, Tang M, Zhang T. Protein corona mitigated the cytotoxicity of CdTe QDs to macrophages by targeting mitochondria. NanoImpact. 2022;25:100367.
20. Baghaee P, Yoonesi M, Esfahani DE, Beirami E, Dargahi L, Rashidi FS, Valian N. Yttrium oxide nanoparticles alleviate cognitive deficits, neuroinflammation, and mitochondrial biogenesis impairment induced by streptozotocin. Neurosci Lett. 2024;837:137895.
21. Shen Y, Wu L, Qin D, Xia Y, Zhou Z, Zhang X, Wu X. Carbon black suppresses the osteogenesis of mesenchymal stem cells: the role of mitochondria. Part Fibre Toxicol. 2018;15(1):16.
22. Zhou H, Yao L, Jiang X, Sumayyah G, Tu B, Cheng S, Qin X, Zhang J, Zou Z, Chen C. Pulmonary Exposure to Copper Oxide Nanoparticles Leads to Neurotoxicity via Oxidative Damage and Mitochondrial Dysfunction. Neurotox Res. 2021;39(4):1160-1170.
23. Wang G, Shen X, Song X, Wang N, Wo X, Gao Y. Protective mechanism of gold nanoparticles on human neural stem cells injured by β-amyloid protein through miR-21-5p/SOCS6 pathway. Neurotoxicology. 2023;95:12-22.
24. Li J, Zhang B, Chang X, Gan J, Li W, Niu S, Kong L, Wu T, Zhang T, Tang M, Xue Y. Silver nanoparticles modulate mitochondrial dynamics and biogenesis in HepG2 cells. Environ Pollut. 2020;256:113430.
25. Chang X, Niu S, Shang M, Li J, Zhang W, Sun Z, Li Y, Wu T, Zhang T, Tang M, Xue Y. Silver nanoparticles induced hippocampal neuronal damage involved in mitophagy, mitochondrial biogenesis and synaptic degeneration. Food Chem Toxicol. 2022;166:113227.
26. Yousef MI, Roychoudhury S, Jafaar KS, Slama P, Kesari KK, Kamel MA. Aluminum oxide and zinc oxide induced nanotoxicity in rat brain, heart, and lung. Physiol Res. 2022 ;71(5):677-694.
27. Xu Z, Li J, Su B, Gao H, Ren M, Lin Y, Shen H. A role of ROS-dependent defects in mitochondrial dynamic and autophagy in carbon black nanoparticle-mediated myocardial cell damage. Free Radic Biol Med. 2024;220:249-261.
28. Yousef MI, Mutar TF, Kamel MAE. Hepato-renal toxicity of oral sub-chronic exposure to aluminum oxide and/or zinc oxide nanoparticles in rats. Toxicol Rep. 2019;6:336-346.

29. Chang X, Niu S, Guo M, Shang M, Guo S, Mou X, Wu T, Tang M, Xue Y. Silver nanoparticles induced synaptic degeneration via Ca^{2+}/CaMKII signal and Drp1-dependent mitochondrial disorder in HT22 cells. Food Chem Toxicol. 2024;186:114577.
30. Nguyen KC, Zhang Y, Todd J, Kittle K, Lalande M, Smith S, Parks D, Navarro M, Tayabali AF, Willmore WG. Hepatotoxicity of Cadmium Telluride Quantum Dots Induced by Mitochondrial Dysfunction. Chem Res Toxicol. 2020;33(9):2286-2297.
31. Wang L, Duan Z, Liang M, Wang C, Liang T, Sun L, Yan C, Li Q, Liang T. A pivotal role of selective autophagy in mitochondrial quality control: Implications for zinc oxide nanoparticles induced neurotoxicity. Chem Biol Interact. 2022;363:110003.
32. Chen Y, Wang Z, Xu M, Wang X, Liu R, Liu Q, Zhang Z, Xia T, Zhao J, Jiang G, Xu Y, Liu S. Nanosilver incurs an adaptive shunt of energy metabolism mode to glycolysis in tumor and nontumor cells. ACS Nano. 2014;8(6):5813-5825.
33. Chang X, Niu S, Shang M, Li J, Guo M, Zhang W, Sun Z, Li Y, Zhang R, Shen X, Tang M, Xue Y. ROS-Drp1-mediated mitochondria fission contributes to hippocampal HT22 cell apoptosis induced by silver nanoparticles. Redox Biol. 2023;63:102739.
34. Wang L, Wang B, Zhang X, Yang Z, Zhang X, Gong H, Song Y, Zhang K, Sun M. TDCPP and TiO_2 NPs aggregates synergistically induce SH-SY5Y cell neurotoxicity by excessive mitochondrial fission and mitophagy inhibition. Environ Pollut. 2024;347:123740.
35. Arslan NP, Keles ON, Gonul-Baltaci N. Effect of Titanium Dioxide and Silver Nanoparticles on Mitochondrial Dynamics in Mouse Testis Tissue. Biol Trace Elem Res. 2022;200(4):1650-1658.
36. Natarajan V, Wilson CL, Hayward SL, Kidambi S. Titanium Dioxide Nanoparticles Trigger Loss of Function and Perturbation of Mitochondrial Dynamics in Primary Hepatocytes. PLoS One. 2015;10(8):e0134541.
37. Ma W, He S, Ma H, Jiang H, Yan N, Zhu L, Bang JJ, Li PA, Jia S. Silver Nanoparticle Exposure Causes Pulmonary Structural Damage and Mitochondrial Dynamic Imbalance in the Rat: Protective Effects of Sodium Selenite. Int J Nanomedicine. 2020;15:633-645.
38. He Y, Yu T, Li H, Sun Q, Chen M, Lin Y, Dai J, Wang W, Li Q, Ju S. Polystyrene nanoplastic exposure actives ferroptosis by oxidative stress-induced lipid peroxidation in porcine oocytes during maturation. J Anim Sci Biotechnol. 2024;15(1):117.
39. Yousef MI, Abd HH, Helmy YM, Kamel MA. Synergistic effect of curcumin and chitosan nanoparticles on nano-hydroxyapatite-induced reproductive toxicity in rats. Environ Sci Pollut Res Int. 2021;28(8):9362-9376.
40. Gutiérrez-Carcedo P, Navalón S, Simó R, Setoain X, Aparicio-Gómez C, Abasolo I, Victor VM, García H, Herance JR. Alteration of the Mitochondrial Effects of Ceria Nanoparticles by Gold: An Approach for the Mitochondrial Modulation of Cells Based on Nanomedicine. Nanomaterials (Basel). 2020;10(4):744.
41. Hong F, Zhou Y, Zhou Y, Wang L. Immunotoxic effects of thymus in mice following exposure to nanoparticulate TiO_2. Environ Toxicol. 2017;32(10):2234-2243.
42. Eldeeb GM, Yousef MI, Helmy YM, Aboudeya HM, Mahmoud SA, Kamel MA. The protective effects of chitosan and curcumin nanoparticles against the hydroxyapatite nanoparticles-induced neurotoxicity in rats. Sci Rep. 2024;14(1):21009.
43. Gurunathan S, Jeyaraj M, La H, Yoo H, Choi Y, Do JT, Park C, Kim JH, Hong K. Anisotropic Platinum Nanoparticle-Induced Cytotoxicity, Apoptosis, Inflammatory Response, and

Transcriptomic and Molecular Pathways in Human Acute Monocytic Leukemia Cells. Int J Mol Sci. 2020;21(2):440.
44. Skóra B, Piechowiak T, Szychowski KA. Engagement of specific intracellular pathways in the inflammation-based reprotoxicity effect of small-size silver nanoparticles on spermatogonia and spermatocytes invitro cell models. Chemosphere. 2024;363:142897.
45. Zhao X, Abulikemu A, Lv S, Qi Y, Duan J, Zhang J, Chen R, Guo C, Li Y, Sun Z. Oxidative stress- and mitochondrial dysfunction-mediated cytotoxicity by silica nanoparticle in lung epithelial cells from metabolomic perspective. Chemosphere. 2021;275:129969.
46. Wei S, Li T, Xie R, Ye B, Xiang J, Liu K, Chen Z, Gao X. The role of ATF3 in ZnO nanoparticle-induced genotoxicity and cytotoxicity in bronchial epithelial cells. Int J Biochem Cell Biol. 2019;113:95-102.
47. Lin C, Zhao X, Sun D, Zhang L, Fang W, Zhu T, Wang Q, Liu B, Wei S, Chen G, Xu Z, Gao X. Transcriptional activation of follistatin by Nrf2 protects pulmonary epithelial cells against silica nanoparticle-induced oxidative stress. Sci Rep. 2016;6:21133.
48. Nguyen KC, Willmore WG, Tayabali AF. Cadmium telluride quantum dots cause oxidative stress leading to extrinsic and intrinsic apoptosis in hepatocellular carcinoma HepG2 cells. Toxicology. 2013;306:114-23.
49. Nguyen KC, Rippstein P, Tayabali AF, Willmore WG. Mitochondrial Toxicity of Cadmium Telluride Quantum Dot Nanoparticles in Mammalian Hepatocytes. Toxicol Sci. 2015;146(1):31-42.
50. Dey S, Fageria L, Sharma A, Mukherjee S, Pande S, Chowdhury R, Chowdhury S. Silver nanoparticle-induced alteration of mitochondrial and ER homeostasis affects human breast cancer cell fate. Toxicol Rep. 2022;9:1977-1984.
51. Zhao X, Xu H, Li Y, Liu Y, Li X, Zhou W, Wang J, Guo C, Sun Z, Li Y. Silica nanoparticles perturbed mitochondrial dynamics and induced myocardial apoptosis via PKA-DRP1-mitochondrial fission signaling. Sci Total Environ. 2022;842:156854.
52. Li J , Chang X , Shang M , Niu S , Zhang W , Li Y , Sun Z , Wu T , Kong L , Zhang T , Tang M , Xue Y . The crosstalk between DRP1-dependent mitochondrial fission and oxidative stress triggers hepatocyte apoptosis induced by silver nanoparticles. Nanoscale. 2021;13(28):12356-12369.
53. Zheng H, Liang G, Guan C, Liu L, Dong J, Zhao J, Tang M, Kong L. Mitochondrial Fission in Nickel Nanoparticle-Induced Reproductive Toxicity: An In Vitro GC-1 Cell Study. Nanomaterials (Basel). 2024;14(8):689.
54. Bittner A, Ducray AD, Stoffel MH, Felser A, Mevissen M. Polymer-coated nanoparticles and their effects on mitochondrial function in brain endothelial cells. Toxicol Appl Pharmacol. 2019;385:114800.
55. Wilson CL, Natarajan V, Hayward SL, Khalimonchuk O, Kidambi S. Mitochondrial dysfunction and loss of glutamate uptake in primary astrocytes exposed to titanium dioxide nanoparticles. Nanoscale. 2015;7(44):18477-18488.
56. Li Y, Zhu Y, Zhao B, Yao Q, Xu H, Lv S, Wang J, Sun Z, Li Y, Guo C. Amorphous silica nanoparticles caused lung injury through the induction of epithelial apoptosis via ROS/Ca^{2+}/DRP1-mediated mitochondrial fission signaling. Nanotoxicology. 2022;16(6-8):713-732.
57. Fan Y, Cheng Z, Mao L, Xu G, Li N, Zhang M, Weng P, Zheng L, Dong X, Hu S, Wang B,

Qin X, Jiang X, Chen C, Zhang J, Zou Z. PINK1/TAX1BP1-directed mitophagy attenuates vascular endothelial injury induced by copper oxide nanoparticles. J Nanobiotechnology. 2022;20(1):149.
58. Ma W, He S, Xu Y, Qi G, Ma H, Bang JJ, Li PA. Ameliorative Effect of Sodium Selenite on Silver Nanoparticles-Induced Myocardiocyte Structural Alterations in Rats. Int J Nanomedicine. 2020;15:8281-8292.
59. Kong L, Dong J, Lu W, Wu Y, Liu L, Tang M. Exposure effects of inhaled nickel nanoparticles on the male reproductive system via mitochondria damage. NanoImpact. 2021;23:100350.
60. Fu Y, Fan M, Xu L, Wang H, Hu Q, Jin Y. Amino-Functionalized Polystyrene Nano-Plastics Induce Mitochondria Damage in Human Umbilical Vein Endothelial Cells. Toxics. 2022 ;10(5):215.
61. Liu Z, Xia X, Lv X, Song E, Song Y. Iron-bearing nanoparticles trigger human umbilical vein endothelial cells ferroptotic responses by promoting intracellular iron level. Environ Pollut. 2021;287:117345.
62. Tian T, Pang H, Li X, Ma K, Liu T, Li J, Luo Z, Li M, Hou Q, Hao H, Dong J, Du H, Liu X, Sun Z, Zhao C, Song X, Jin M. The role of DRP1 mediated mitophagy in HT22 cells apoptosis induced by silica nanoparticles. Ecotoxicol Environ Saf. 2024;272:116050.
63. Ko WC, Shieh JM, Wu WB. P38 MAPK and Nrf2 Activation Mediated Naked Gold Nanoparticle Induced Heme Oxygenase-1 Expression in Rat Aortic Vascular Smooth Muscle Cells. Arch Med Res. 2020;51(5):388-396.
64. Zhang X, Li M, Wu H, Fan W, Zhang J, Su W, Wang Y, Li P. Naringenin attenuates inflammation, apoptosis, and ferroptosis in silver nanoparticle-induced lung injury through a mechanism associated with Nrf2/HO-1 axis: In vitro and in vivo studies. Life Sci. 2022;311(Pt A):121127.
65. Liu N, Liang Y, Wei T, Zou L, Huang X, Kong L, Tang M, Zhang T. The role of ferroptosis mediated by NRF2/ERK-regulated ferritinophagy in CdTe QDs-induced inflammation in macrophage. J Hazard Mater. 2022;436:129043.
66. Vineetha VP, Devika P, Prasitha K, Anilkumar TV. Tinospora cordifolia ameliorated titanium dioxide nanoparticle-induced toxicity via regulating oxidative stress-activated MAPK and NRF2/Keap1 signaling pathways in Nile tilapia (Oreochromis niloticus). Comp Biochem Physiol C Toxicol Pharmacol. 2021;240:108908.
67. Zhai S, Zhang X, Jiang M, Liu Y, Qu G, Cui X, Hirschbiegel CM, Liu Y, Alves C, Lee YW, Jiang G, Yan B, Rotello VM. Nanoparticles with intermediate hydrophobicity polarize macrophages to plaque-specific Mox phenotype via Nrf2 and HO-1 activation. J Hazard Mater. 2024;466:133590.
68. Yin Y, Peng H, Shao J, Zhang J, Li Y, Pi J, Guo J. NRF2 deficiency sensitizes human keratinocytes to zinc oxide nanoparticles-induced autophagy and cytotoxicity. Environ Toxicol Pharmacol. 2021;87:103721.
69. Li N, Du H, Mao L, Xu G, Zhang M, Fan Y, Dong X, Zheng L, Wang B, Qin X, Jiang X, Chen C, Zou Z, Zhang J. Reciprocal regulation of NRF2 by autophagy and ubiquitin-proteasome modulates vascular endothelial injury induced by copper oxide nanoparticles. J Nanobiotechnology. 2022;20(1):270.
70. Mahmoud AM, Desouky EM, Hozayen WG, Bin-Jumah M, El-Nahass ES, Soliman HA,

Farghali AA. Mesoporous Silica Nanoparticles Trigger Liver and Kidney Injury and Fibrosis Via Altering TLR4/NF-κB, JAK2/STAT3 and Nrf2/HO-1 Signaling in Rats. Biomolecules. 2019;9(10):528.

71. Sun X, Yang Y, Shi J, Wang C, Yu Z, Zhang H. NOX4- and Nrf2-mediated oxidative stress induced by silver nanoparticles in vascular endothelial cells. J Appl Toxicol. 2017;37(12):1428-1437.
72. Hong F, Zhou Y. Spermatogenic Apoptosis and the Involvement of the Nrf2 Pathway in Male Mice Following Exposure to Nano Titanium Dioxide. J Biomed Nanotechnol. 2020;16(3):373-381.
73. Wang M, Zheng H, Chen J, Tang Y, Feng M, Li L. ZnO nanoparticles impair autophagic flux and cell viability through the TRIM16-NRF2-p62 pathway in inflammatory keratinocytes. Food Chem Toxicol. 2023;182:114177.
74. Wen Y, Deng S, Wang B, Zhang F, Luo T, Kuang H, Kuang X, Yuan Y, Huang J, Zhang D. Exposure to polystyrene nanoplastics induces hepatotoxicity involving NRF2-NLRP3 signaling pathway in mice. Ecotoxicol Environ Saf. 2024;278:116439.
75. Maher AM, Elsanosy GA, Ghareeb DA, Elblehi SS, Saleh SR. 10-Hydroxy Decanoic Acid and Zinc Oxide Nanoparticles Retrieve Nrf2/HO-1 and Caspase-3/Bax/Bcl-2 Signaling in Lead-Induced Testicular Toxicity. Biol Trace Elem Res. 2025;203(5):2728-2751.
76. Chen T, Zhang L, Yao L, Luan J, Zhou X, Cong R, Guo X, Qin C, Song N. Zinc oxide nanoparticles-induced testis damage at single-cell resolution: Depletion of spermatogonia reservoir and disorder of Sertoli cell homeostasis. Environ Int. 2023;181:108292.
77. Liu W, Hu T, Zhou L, Wu D, Huang X, Ren X, Lv Y, Hong W, Huang G, Lin Z, Liu J. Nrf2 protects against oxidative stress induced by SiO2 nanoparticles. Nanomedicine (Lond). 2017;12(19):2303-2318.
78. Shi Z, Niu Y, Wang Q, Shi L, Guo H, Liu Y, Zhu Y, Liu S, Liu C, Chen X, Zhang R. Reduction of DNA damage induced by titanium dioxide nanoparticles through Nrf2 in vitro and in vivo. J Hazard Mater. 2015;298:310-319.
79. Zhang L, Zou L, Jiang X, Cheng S, Zhang J, Qin X, Qin Z, Chen C, Zou Z. Stabilization of Nrf2 leading to HO-1 activation protects against zinc oxide nanoparticles-induced endothelial cell death. Nanotoxicology. 2021;15(6):779-797.
80. Guo C, Xia Y, Niu P, Jiang L, Duan J, Yu Y, Zhou X, Li Y, Sun Z. Silica nanoparticles induce oxidative stress, inflammation, and endothelial dysfunction in vitro via activation of the MAPK/Nrf2 pathway and nuclear factor-κB signaling. Int J Nanomedicine. 2015;10:1463-77.
81. Elblehi SS, Abd El-Maksoud EM, Aldhahrani A, Alotaibi SS, Ghamry HI, Elgendy SA, Soliman MM, Shukry M. Quercetin Abrogates Oxidative Neurotoxicity Induced by Silver Nanoparticles in Wistar Rats. Life (Basel). 2022;12(4):578.
82. Abdou KH, Moselhy WA, Mohamed HM, El-Nahass ES, Khalifa AG. Moringa oleifera Leaves Extract Protects Titanium Dioxide Nanoparticles-Induced Nephrotoxicity via Nrf2/HO-1 Signaling and Amelioration of Oxidative Stress. Biol Trace Elem Res. 2019;187(1):181-191.
83. Kang SJ, Ryoo IG, Lee YJ, Kwak MK. Role of the Nrf2-heme oxygenase-1 pathway in silver nanoparticle-mediated cytotoxicity. Toxicol Appl Pharmacol. 2012;258(1):89-98.
84. Zhang H, Zhou L, Yuen J, Birkner N, Leppert V, O'Day PA, Forman HJ. Delayed Nrf2-regulated antioxidant gene induction in response to silica nanoparticles. Free Radic Biol

Med. 2017;108:311-319.

85. u X, Han H, Yang H, Xu B, Dai W, Liu L, He T, DU X, Pei X. Nrf2-mediated ferroptosis of spermatogenic cells involved in male reproductive toxicity induced by polystyrene nanoplastics in mice. J Zhejiang Univ Sci B. 2024;25(4):307-323.
86. Guo M, Zhang W, Niu S, Shang M, Chang X, Wu T, Zhang T, Tang M, Xue Y. Adaptive regulations of Nrf2 alleviates silver nanoparticles-induced oxidative stress-related liver cells injury. Chem Biol Interact. 2023;369:110287.
87. Abdelrahman SA, Mahmoud AA, Abdelrahman AA, Samy W, Zaid Hassen Saleh E. Histomorphological changes and molecular mechanisms underlying the ameliorative effect of resveratrol on the liver of silver nanoparticles-exposed rats. Ultrastruct Pathol. 2022;46(3):268-284.
88. Teng J, Yu T, Yan F. GABA attenuates neurotoxicity of zinc oxide nanoparticles due to oxidative stress via DAF-16/FoxO and SKN-1/Nrf2 pathways. Sci Total Environ. 2024 ;934:173214.
89. Ma Y, Wang L, He J, Ma X, Wang J, Yan R, Ma W, Ma H, Liu Y, Sun H, Zhang X, Jia S, Wang H. Sodium Selenite Ameliorates Silver Nanoparticles Induced Vascular Endothelial Cytotoxic Injury by Antioxidative Properties and Suppressing Inflammation Through Activating the Nrf2 Signaling Pathway. Biol Trace Elem Res. 2024;202(10):4567-4585.
90. Cui G, Li Z, Cao F, Li P, Jin M, Hou S, Yang X, Mu Y, Peng C, Shao H, Du Z. Activation of Nrf2/HO-1 signaling pathway attenuates ROS-mediated autophagy induced by silica nanoparticles in H9c2 cells. Environ Toxicol. 2021;36(7):1389-1401.
91. Huang J, Zou L, Bao M, Feng Q, Xia W, Zhu C. Toxicity of polystyrene nanoparticles for mouse ovary and cultured human granulosa cells. Ecotoxicol Environ Saf. 2023;249:114371.
92. Zheng F, Luo Z, Zheng C, Li J, Zeng J, Yang H, Chen J, Jin Y, Aschner M, Wu S, Zhang Q, Li H. Comparison of the neurotoxicity associated with cobalt nanoparticles and cobalt chloride in Wistar rats. Toxicol Appl Pharmacol. 2019;369:90-99.
93. Hashim AR, Bashir DW, Rashad E, Galal MK, Rashad MM, Khalil HMA, Deraz NM, S M EG. Neuroprotective Assessment of Betaine against Copper Oxide Nanoparticle-Induced Neurotoxicity in the Brains of Albino Rats: A Histopathological, Neurochemical, and Molecular Investigation. ACS Chem Neurosci. 2024;15(8):1684-1701.
94. Wu Z, Yang H, Archana G, Rakshit M, Ng KW, Tay CY. Human keratinocytes adapt to ZnO nanoparticles induced toxicity via complex paracrine crosstalk and Nrf2-proteasomal signal transduction. Nanotoxicology. 2018;12(10):1215-1229.
95. Wang Y, Chang W, Li X, Jiang Z, Zhou D, Feng Y, Li B, Chen G, Li N. Apigenin exerts chemopreventive effects on lung injury induced by SiO_2 nanoparticles through the activation of Nrf2. J Nat Med. 2022;76(1):119-131.
96. Yang J, Liu J, Wang P, Sun J, Lv X, Diao Y. Toxic effect of titanium dioxide nanoparticles on corneas in vitro and in vivo. Aging (Albany NY). 2021;13(4):5020-5033.
97. Salama B, Alzahrani KJ, Alghamdi KS, Al-Amer O, Hassan KE, Elhefny MA, Albarakati AJA, Alharthi F, Althagafi HA, Al Sberi H, Amin HK, Lokman MS, Alsharif KF, Albrakati A, Abdel Moneim AE, Kassab RB, Fathalla AS. Silver Nanoparticles Enhance Oxidative Stress, Inflammation, and Apoptosis in Liver and Kidney Tissues: Potential Protective Role of Thymoquinone. Biol Trace Elem Res. 2023;201(6):2942-2954.
99. Fonseca E, Vázquez M, Rodriguez-Lorenzo L, Mallo N, Pinheiro I, Sousa ML, Cabaleiro S,

Quarato M, Spuch-Calvar M, Correa-Duarte MA, López-Mayán JJ, Mackey M, Moreda A, Vasconcelos V, Espiña B, Campos A, Araújo MJ. Getting fat and stressed: Effects of dietary intake of titanium dioxide nanoparticles in the liver of turbot Scophthalmus maximus. J Hazard Mater. 2023;458:131915.

99. Hou J, Zhao L, Tang H, He X, Ye G, Shi F, Kang M, Chen H, Li Y. Silver Nanoparticles Induced Oxidative Stress and Mitochondrial Injuries Mediated Autophagy in HC11 Cells Through Akt/AMPK/mTOR Pathway. Biol Trace Elem Res. 2021;199(3):1062-1073.
100. Kandeil MA, Mohammed ET, Hashem KS, Aleya L, Abdel-Daim MM. Moringa seed extract alleviates titanium oxide nanoparticles (TiO_2-NPs)-induced cerebral oxidative damage, and increases cerebral mitochondrial viability. Environ Sci Pollut Res Int. 2020;27(16):19169-19184.
101. Alshammari GM, Abdelhalim MA, Al-Ayed MS, Al-Harbi LN, Yahya MA. Concomitant Sub-Chronic Administration of Small-Size Gold Nanoparticles Aggravates Doxorubicin-Induced Liver Oxidative and Inflammatory Damage, Hyperlipidemia, and Hepatic Steatosis. Molecules. 2023;28(2):796.
102. Nemmar A, Al-Salam S, Beegam S, Yuvaraju P, Ali BH. Aortic Oxidative Stress, Inflammation and DNA Damage Following Pulmonary Exposure to Cerium Oxide Nanoparticles in a Rat Model of Vascular Injury. Biomolecules. 2019;9(8):376.
103. Li X, Li D, Zhang G, Zeng Y, Monteiro-Riviere NA, Chang YZ, Li Y. Biocorona modulates the inflammatory response induced by gold nanoparticles in human epidermal keratinocytes. Toxicol Lett. 2022;369:34-42.
104. Li T, Li Z, Fu J, Tang C, Liu L, Xu J, Zhao J, Li Z. Nickel nanoparticles exert cytotoxic effects on trophoblast HTR-8/SVneo cells possibly via Nrf2/MAPK/caspase 3 pathway. Environ Res. 2022;215(Pt 2):114336.
105. Zhou D, Chang W, Qi J, Chen G, Li N. Lung protective effects of dietary malate esters derivatives from Bletilla striata against SiO_2 nanoparticles through activation of Nrf2 pathway. Chin Herb Med. 2022;15(1):76-85.
106. Zou L, Cheng G, Xu C, Liu H, Wang Y, Li N, Fan X, Zhu C, Xia W. Copper Nanoparticles Induce Oxidative Stress via the Heme Oxygenase 1 Signaling Pathway in vitro Studies. Int J Nanomedicine. 2021;16:1565-1573.
107. Korakaki E, Simos YV, Karouta N, Spyrou K, Zygouri P, Gournis DP, Tsamis KI, Stamatis H, Dounousi E, Vezyraki P, Peschos D. Effect of Highly Hydrophilic Superparamagnetic Iron Oxide Nanoparticles on Macrophage Function and Survival. J Funct Biomater. 2023;14(10):514.
108. Li X, Zhang H, Sun F. CdSe/ZnS quantum dots exhibited nephrotoxicity through mediating oxidative damage and inflammatory response. Aging. 2020;13(8):12194-12206.
109. Afshari-Kaveh M, Abbasalipourkabir R, Nourian A, Ziamajidi N. The Protective Effects of Vitamins A and E on Titanium Dioxide Nanoparticles ($nTiO_2$)-Induced Oxidative Stress in the Spleen Tissues of Male Wistar Rats. Biol Trace Elem Res. 2021;199(10):3677-3687.
110. Rajkumar KS, Sivagaami P, Ramkumar A, Murugadas A, Srinivasan V, Arun S, Senthil Kumar P, Thirumurugan R. Bio-functionalized zinc oxide nanoparticles: Potential toxicity impact on freshwater fish Cyprinus carpio. Chemosphere. 2022; 290:133220.
111. Magaye R, Gu Y, Wang Y, Su H, Zhou Q, Mao G, Shi H, Yue X, Zou B, Xu J, Zhao J. In vitro and in vivo evaluation of the toxicities induced by metallic nickel nano and fine particles. J

Mol Histol. 2016;47(3):273-286.
112. Noshy PA, Yasin NAE, Rashad MM, Shehata AM, Salem FMS, El-Saied EM, Mahmoud MY. Zinc nanoparticles ameliorate oxidative stress and apoptosis induced by silver nanoparticles in the brain of male rats. Neurotoxicology. 2023;95:193-204.
113. Zhu B, He W, Hu S, Kong R, Yang L. The fate and oxidative stress of different sized SiO_2 nanoparticles in zebrafish (Danio rerio) larvae. Chemosphere. 2019;225:705-712
114. Zhou F, Liao F, Chen L, Liu Y, Wang W, Feng S. The size-dependent genotoxicity and oxidative stress of silica nanoparticles on endothelial cells. Environ Sci Pollut Res Int. 2019;26(2):1911-1920.
115. Liao F, Chen L, Liu Y, Zhao D, Peng W, Wang W, Feng S. The size-dependent genotoxic potentials of titanium dioxide nanoparticles to endothelial cells. Environ Toxicol. 2019;34(11):1199-1207.
116. Mytych J, Wnuk M, Rattan SI. Low doses of nanodiamonds and silica nanoparticles have beneficial hormetic effects in normal human skin fibroblasts in culture. Chemosphere. 2016;148:307-315.
117. Yasin NAE, El-Naggar ME, Ahmed ZSO, Galal MK, Rashad MM, Youssef AM, Elleithy EMM. Exposure to Polystyrene nanoparticles induces liver damage in rat via induction of oxidative stress and hepatocyte apoptosis. Environ Toxicol Pharmacol. 2022;94:103911.
118. González-Fernández C, Díaz Baños FG, Esteban MÁ, Cuesta A. Functionalized Nanoplastics (NPs) Increase the Toxicity of Metals in Fish Cell Lines. Int J Mol Sci. 2021;22(13):7141.
119. Tian J, Hu J, Liu G, Yin H, Chen M, Miao P, Bai P, Yin J. Altered Gene expression of ABC transporters, nuclear receptors and oxidative stress signaling in zebrafish embryos exposed to CdTe quantum dots. Environ Pollut. 2019;244:588-599.
120. Krishnasamy Sekar R, Arunachalam R, Anbazhagan M, Palaniyappan S, Veeran S, Sridhar A, Ramasamy T. Accumulation, Chronicity, and Induction of Oxidative Stress Regulating Genes Through Allium cepa L. Functionalized Silver Nanoparticles in Freshwater Common Carp (*Cyprinus carpio*). Biol Trace Elem Res. 2023;201(2):904-925
121. Skočaj M, Bizjak M, Strojan K, Lojk J, Erdani Kreft M, Miš K, Pirkmajer S, Bregar VB, Veranič P, Pavlin M. Proposing Urothelial and Muscle In Vitro Cell Models as a Novel Approach for Assessment of Long-Term Toxicity of Nanoparticles. Int J Mol Sci. 2020;21(20):7545.
122. Niu L, Shao M, Liu Y, Hu J, Li R, Xie H, Zhou L, Shi L, Zhang R, Niu Y. Reduction of oxidative damages induced by titanium dioxide nanoparticles correlates with induction of the Nrf2 pathway by GSPE supplementation in mice. Chem Biol Interact. 2017;275:133-144.
123. Santacruz-Márquez R, Flaws JA, Sánchez-Peña LDC, Hernández-Ochoa I. Exposure to Zinc Oxide Nanoparticles Increases Estradiol Levels and Induces an Antioxidant Response in Antral Ovarian Follicles In Vitro. Toxics. 2023;11(7):602.
124. Berg JM, Romoser AA, Figueroa DE, Spencer West C, Sayes CM. Comparative cytological responses of lung epithelial and pleural mesothelial cells following in vitro exposure to nanoscale SiO_2. Toxicol In Vitro. 2013;27(1):24-33.
125. Ferraro SA, Domingo MG, Etcheverrito A, Olmedo DG, Tasat DR. Neurotoxicity mediated by oxidative stress caused by titanium dioxide nanoparticles in human neuroblastoma (SH-SY5Y) cells. J Trace Elem Med Biol. 2020;57:126413.
126. Sun Q, Tan D, Zhou Q, Liu X, Cheng Z, Liu G, Zhu M, Sang X, Gui S, Cheng J, Hu R, Tang

M, Hong F. Oxidative damage of lung and its protective mechanism in mice caused by long-term exposure to titanium dioxide nanoparticles. J Biomed Mater Res A. 2012;100(10):2554-2562.

127. Ucar A, Parlak V, Ozgeris FB, Yeltekin AC, Arslan ME, Alak G, Turkez H, Kocaman EM, Atamanalp M. Magnetic nanoparticles-induced neurotoxicity and oxidative stress in brain of rainbow trout: Mitigation by ulexite through modulation of antioxidant, anti-inflammatory, and antiapoptotic activities. Sci Total Environ. 2022;838(Pt 1):155718.
128. Guo C, Yang M, Jing L, Wang J, Yu Y, Li Y, Duan J, Zhou X, Li Y, Sun Z. Amorphous silica nanoparticles trigger vascular endothelial cell injury through apoptosis and autophagy via reactive oxygen species-mediated MAPK/Bcl-2 and PI3K/Akt/mTOR signaling. Int J Nanomedicine. 2016;11:5257-5276.
129. Li Y, Pan M, Meng S, Xu W, Wang S, Dou M, Zhang C. The Effects of Zinc Oxide Nanoparticles on Antioxidation, Inflammation, Tight Junction Integrity, and Apoptosis in Heat-Stressed Bovine Intestinal Epithelial Cells In Vitro. Biol Trace Elem Res. 2024;202(5):2042-2051.
130. Kaur K, Narang RK, Singh S. Neuroprotective potential of Betulinic acid against TIO_2NP induced neurotoxicity in zebrafish. Int Immunopharmacol. 2024;138:112604..
131. Qiao L, Dou X, Yan S, Zhang B, Xu C. Biogenic selenium nanoparticles synthesized by Lactobacillus casei ATCC 393 alleviate diquat-induced intestinal barrier dysfunction in C57BL/6 mice through their antioxidant activity. Food Funct. 2020;11(4):3020-3031.
132. Hassanen EI, Morsy EA, Hussien AM, Ibrahim MA, Farroh KY. The effect of different concentrations of gold nanoparticles on growth performance, toxicopathological and immunological parameters of broiler chickens. Biosci Rep. 2020;40(3):BSR20194296.
133. Liu W, Liu H, Zhang S, Hao H, Meng F, Ma W, Guo Z, Jiang S, Shang X. Silica nanoparticles cause ovarian dysfunction and fertility decrease in mice via oxidative stress-activated autophagy and apoptosis. Ecotoxicol Environ Saf. 2024;285:117049.
134. Wang H, Ni J, Guo X, Zhou T, Ma X, Xue J, Wang X. Shelterin differentially respond to oxidative stress induced by TiO2-NPs and regulate telomere length in human hepatocytes and hepatocarcinoma cells in vitro. Biochem Biophys Res Commun. 2018;503(2):697-702.
135. Eom HJ, Choi J. Oxidative stress of silica nanoparticles in human bronchial epithelial cell, Beas-2B. Toxicol In Vitro. 2009;23(7):1326-1332.
136. Liu X, Tu B, Jiang X, Xu G, Bai L, Zhang L, Meng P, Qin X, Chen C, Zou Z. Lysosomal dysfunction is associated with persistent lung injury in dams caused by pregnancy exposure to carbon black nanoparticles. Life Sci. 2019;233:116741.
137. Wang J, Li N, Zheng L, Wang S, Wang Y, Zhao X, Duan Y, Cui Y, Zhou M, Cai J, Gong S, Wang H, Hong F. P38-Nrf-2 signaling pathway of oxidative stress in mice caused by nanoparticulate TiO2. Biol Trace Elem Res. 2011;140(2):186-197.
138. Eom HJ, Choi J. SiO_2 Nanoparticles Induced Cytotoxicity by Oxidative Stress in Human Bronchial Epithelial Cell, Beas-2B. Environ Health Toxicol. 2011;26:e2011013.
139. Liang Y, Wang Z, Huo D, Hu JN, Song L, Ma X, Jiang S, Li W. Nanoplastic-Induced Liver Damage Was Alleviated by Maltol via Enhancing Autophagic Flow: An In Vivo and In Vitro Study. J Agric Food Chem. 2024;72(29):16250-16262..
140. Dhupal M, Oh JM, Tripathy DR, Kim SK, Koh SB, Park KS. Immunotoxicity of titanium dioxide nanoparticles via simultaneous induction of apoptosis and multiple toll-like receptors

signaling through ROS-dependent SAPK/JNK and p38 MAPK activation. Int J Nanomedicine. 2018;13:6735-6750.
141. Brown DM, Kanase N, Gaiser B, Johnston H, Stone V. Inflammation and gene expression in the rat lung after instillation of silica nanoparticles: effect of size, dispersion medium and particle surface charge. Toxicol Lett. 2014;224(1):147-156.
142. da Rocha AM, Ferreira JR, Barros DM, Pereira TC, Bogo MR, Oliveira S, Geraldo V, Lacerda RG, Ferlauto AS, Ladeira LO, Pinheiro MV, Monserrat JM. Gene expression and biochemical responses in brain of zebrafish Danio rerio exposed to organic nanomaterials: carbon nanotubes (SWCNT) and fullerenol (C60(OH)18-22(OK4)). Comp Biochem Physiol A Mol Integr Physiol. 2013;165(4):460-467.
143. Eom HJ, Choi J. Oxidative stress of CeO_2 nanoparticles via p38-Nrf-2 signaling pathway in human bronchial epithelial cell, Beas-2B. Toxicol Lett. 2009;187(2):77-83.
144. Ze Y, Zheng L, Zhao X, Gui S, Sang X, Su J, Guan N, Zhu L, Sheng L, Hu R, Cheng J, Cheng Z, Sun Q, Wang L, Hong F. Molecular mechanism of titanium dioxide nanoparticles-induced oxidative injury in the brain of mice. Chemosphere. 2013;92(9):1183-1189.
145. Kluknavsky M, Micurova A, Skratek M, Balis P, Okuliarova M, Manka J, Bernatova I. A Single Infusion of Polyethylene Glycol-Coated Superparamagnetic Magnetite Nanoparticles Alters Differently the Expressions of Genes Involved in Iron Metabolism in the Liver and Heart of Rats. Pharmaceutics. 2023;15(5):1475
146.Eid A, Ghaleb SS, Zaki A, Ibrahim M, Farghali AA, Ali LE, Abdelgawad MA, Ghoneim MM, Al-Serwi RH, Hassan RM, Ahmad M. Hesperidin Attenuates Titanium Dioxide Nanoparticle-Induced Neurotoxicity in Rats by Regulating Nrf-2/TNF-α Signaling Pathway, the Suppression of Oxidative Stress, and Inflammation. ACS Omega. 2023;8(40):37584-37591.
147. Alghriany AAI, Omar HEM, Mahmoud AM, Atia MM. Assessment of the Toxicity of Aluminum Oxide and Its Nanoparticles in the Bone Marrow and Liver of Male Mice: Ameliorative Efficacy of Curcumin Nanoparticles. ACS Omega. 2022;7(16):13841-13852.
148. Wei S, Mao Y, Sokolova IM, Li Z, Li L, Khalid MS, Tu Z, Zhong Z, Hu M, Wang Y. Extreme heat event influences the toxic impacts of nano-TiO2 with different crystal structures in mussel Mytilus coruscus. Sci Total Environ. 2024;955:176916.
149. Voicu SN, Balas M, Stan MS, Trică B, Serban AI, Stanca L, Hermenean A, Dinischiotu A. Amorphous Silica Nanoparticles Obtained by Laser Ablation Induce Inflammatory Response in Human Lung Fibroblasts. Materials (Basel). 2019;12(7):1026.
150. Micurova A, Kluknavsky M, Liskova S, Balis P, Skratek M, Okruhlicova L, Manka J, Bernatova I. Differences in Distribution and Biological Effects of F_3O_4@PEG Nanoparticles in Normotensive and Hypertensive Rats-Focus on Vascular Function and Liver. Biomedicines. 2021;9(12):1855.
151. Abd-Eltawab Tammam A, A Khalaf AA, R Zaki A, Mansour Khalifa M, A Ibrahim M, M Mekkawy A, E Abdelrahman R, Farghali A, A Noshy P. Hesperidin protects rats' liver and kidney from oxidative damage and physiological disruption induced by nickel oxide nanoparticles. Front Physiol. 2022;13:912625.
152. Dou X, Qiao L, Song X, Chang J, Zeng X, Zhu L, Deng T, Yang G, Xu C. Biogenic selenium nanoparticles alleviate intestinal barrier injury in mice through TBC1D15/Fis1/Rab7 pathway.

Biomed Pharmacother. 2024;175:116740.

153. Kuang H, Zhang W, Yang L, Aguilar ZP, Xu H. Reproductive organ dysfunction and gene expression after orally administration of ZnO nanoparticles in murine. Environ Toxicol. 2021; 36(4):550-561
154. Yin X, Li Z, Lyu C, Wang Y, Ding S, Ma C, Wang J, Cui S, Wang J, Guo D, Xu R. Induced effect of zinc oxide nanoparticles on human acute myeloid leukemia cell apoptosis by regulating mitochondrial division. IUBMB Life. 2022;74(6):519-531.
155. Li X, Kang B, Eom Y, Zhong J, Lee HK, Kim HM, Song JS. Comparison of cytotoxicity effects induced by four different types of nanoparticles in human corneal and conjunctival epithelial cells. Sci Rep 2022;12(1):155.
156. Yang S, Zhang T, Ge Y, Yin L, Pu Y, Liang G. Inhalation exposure to polystyrene nanoplastics induces chronic obstructive pulmonary disease-like lung injury in mice through multi-dimensional assessment. Environ Pollut. 2024;347:123633.
157. Khan AA, Alanazi AM, Alsaif N, Al-Anazi M, Sayed AYA, Bhat MA. Potential cytotoxicity of silver nanoparticles: Stimulation of autophagy and mitochondrial dysfunction in cardiac cells. Saudi J Biol Sci. 2021;28(5):2762-2771.
158. Sapienza S, Tedeschi V, Apicella B, Palestra F, Russo C, Piccialli I, Pannaccione A, Loffredo S, Secondo A. Size-Based Effects of Anthropogenic Ultrafine Particles on Lysosomal TRPML1 Channel and Autophagy in Motoneuron-like Cells. Int J Mol Sci. 2022;23(21):13041.
159. Li Y, Guo M, Niu S, Shang M, Chang X, Sun Z, Zhang R, Shen X, Xue Y. ROS and DRP1 interactions accelerate the mitochondrial injury induced by polystyrene nanoplastics in human liver HepG2 cells. Chem Biol Interact. 2023;379:110502.
160. Li X, Piao J, Kang B, Eom Y, Kim DH, Song JS. The toxic effects of polystyrene microplastic/nanoplastic particles on retinal pigment epithelial cells and retinal tissue. Environ Sci Pollut Res Int. 2024;31(42):54950-54961.
161. Liang Y, Yang Y, Lu C, Cheng Y, Jiang X, Yang B, Li Y, Chen Q, Ao L, Cao J, Han F, Liu J, Zhao L. Polystyrene nanoplastics exposure triggers spermatogenic cell senescence via the Sirt1/ROS axis. Ecotoxicol Environ Saf. 2024;279:116461.
162. Bai H, Wu Y, Li H, Zhu Y, Che R, Wang F, Zhang C. Cerebral neurotoxicity of amino-modified polystyrene nanoplastics in mice and the protective effects of functional food Camellia pollen. Sci Total Environ. 2024;912:169511.
163. Shiwakoti S, Ko JY, Gong D, Dhakal B, Lee JH, Adhikari R, Gwak Y, Park SH, Jun Choi I, Schini-Kerth VB, Kang KW, Oak MH. Effects of polystyrene nanoplastics on endothelium senescence and its underlying mechanism. Environ Int. 2022;164:107248.
164. Shen J, Dong J, Shao F, Zhao J, Gong L, Wang H, Chen W, Zhang Y, Cai Y. Graphene oxide induces autophagy and apoptosis via the ROS-dependent AMPK/mTOR/ULK-1 pathway in colorectal cancer cells. Nanomedicine (Lond). 2022;17(9):591-605.
165. Tang Y, Zhao R, Pu Q, Jiang S, Yu F, Yang Z, Han T. Investigation of nephrotoxicity on mice exposed to polystyrene nanoplastics and the potential amelioration effects of DHA-enriched phosphatidylserine. Sci Total Environ. 2023;892:164808.
166. Chen W, Chu Q, Ye X, Sun Y, Liu Y, Jia R, Li Y, Tu P, Tang Q, Yu T, Chen C, Zheng X. Canidin-3-glucoside prevents nano-plastics induced toxicity via activating autophagy and promoting discharge. Environ Pollut. 2021;274:116524.

167. Huang Y, Liang B, Li Z, Zhong Y, Wang B, Zhang B, Du J, Ye R, Xian H, Min W, Yan X, Deng Y, Feng Y, Bai R, Fan B, Yang X, Huang Z. Polystyrene nanoplastic exposure induces excessive mitophagy by activating AMPK/ULK1 pathway in differentiated SH-SY5Y cells and dopaminergic neurons in vivo. Part Fibre Toxicol. 2023;20(1):44.
168. Zhang C, Li Y, Yu H, Ye L, Li T, Zhang X, Wang C, Li P, Ji H, Gao Q, Dong S. Nanoplastics promote arsenic-induced ROS accumulation, mitochondrial damage and disturbances in neurotransmitter metabolism of zebrafish (Danio rerio). Sci Total Environ. 2023;863:161005.
169. Fernandes AL, Nascimento JP, Santos AP, Furtado CA, Romano LA, Eduardo da Rosa C, Monserrat JM, Ventura-Lima J. Assessment of the effects of graphene exposure in Danio rerio: A molecular, biochemical and histological approach to investigating mechanisms of toxicity. Chemosphere. 2018;210:458-466.
170. Brown DM, Donaldson K, Stone V. Nuclear translocation of Nrf2 and expression of antioxidant defence genes in THP-1 cells exposed to carbon nanotubes. J Biomed Nanotechnol. 2010;6(3):224-233.
171. Pelka J, Gehrke H, Rechel A, Kappes M, Hennrich F, Hartinger CG, Marko D. DNA damaging properties of single walled carbon nanotubes in human colon carcinoma cells. Nanotoxicology. 2013;7(1):2-20.
172. Stanca L, Geicu OI, Serban AI, Dinischiotu A. Interplay of Oxidative Stress, Inflammation, and Autophagy in RAW 264.7 Murine Macrophage Cell Line Challenged with Si/SiO_2 Quantum Dots. Materials (Basel). 2023;16(14):5083.
173. Guo M, Li Y, Niu S, Zhang R, Shen X, Ma Y, Wu L, Wu T, Zhang T, Tang M, Xue Y. Oxidative stress-activated Nrf2 remitted polystyrene nanoplastic-induced mitochondrial damage and inflammatory response in HepG2 cells. Environ Toxicol Pharmacol. 2024;106:104385.
174. Sun R, Liu M, Xiong F, Xu K, Huang J, Liu J, Wang D, Pu Y. Polystyrene micro- and nanoplastics induce gastric toxicity through ROS mediated oxidative stress and P62/Keap1/Nrf2 pathway. Sci Total Environ. 2024;912:169228.
175. Wang W, Zhou C, Ma Z, Zeng L, Wang H, Cheng X, Zhang C, Xue Y, Yuan Y, Li J, Hu L, Huang J, Luo T, Zheng L. Co-exposure to polystyrene nanoplastics and triclosan induces synergistic cytotoxicity in human KGN granulosa cells by promoting reactive oxygen species accumulation. Ecotoxicol Environ Saf. 2024;273:116121.
176. Wu Y, Li L, Tang L, Peijnenburg W, Zhang H, Xie D, Geng R, Zheng T, Bi L, Wei X, Chae HJ, Wang L, Zhao L, Li B, Zheng Q. Ototoxicity of polystyrene nanoplastics in mice, HEI-OC1 cells and zebrafish. Front Mol Neurosci. 2024;17:1345536.
177. Ling X, Zuo J, Pan M, Nie H, Shen J, Yang Q, Hung TC, Li G. The presence of polystyrene nanoplastics enhances the MCLR uptake in zebrafish leading to the exacerbation of oxidative liver damage. Sci Total Environ. 2022;818:151749..
178. Ijaz MU, Nadeem N, Hamza A, Almutairi MH, Atique U. Didymin protects against polystyrene nanoplastic-induced hepatic damage in male albino rats by modulation of Nrf-2/Keap-1 pathway. Braz J Med Biol Res. 2024;57:e13173.
179. Liu L, Lu W, Dong J, Wu Y, Tang M, Liang G, Kong L. Study of the mechanism of mitochondrial division and mitochondrial autophagy in the male reproductive toxicity induced by nickel nanoparticles. Nanoscale. 2022;14(5):1868-1884.
180. Feng X, Zhang Y, Luo R, Lai X, Chen A, Zhang Y, Chen H, Hu C, Chen L, Shao L. Graphene

oxide disrupted mitochondrial homeostasis through inducing intracellular redox deviation and autophagy-lysosomal network dysfunction in SH-SY5Y cells. J Hazard Mater. 2021 Aug 15;416:126158..

181. Duan WX, He MD, Mao L, Qian FH, Li YM, Pi HF, Liu C, Chen CH, Lu YH, Cao ZW, Zhang L, Yu ZP, Zhou Z. NiO nanoparticles induce apoptosis through repressing SIRT1 in human bronchial epithelial cells. Toxicol Appl Pharmacol. 2015;286(2):80-91.
182. Zhao X, Wu Y, Li J, Li D, Jin Y, Zhu P, Liu Y, Zhuang Y, Yu S, Cao W, Wei H, Wang X, Han Y, Chen G. JNK activation-mediated nuclear SIRT1 protein suppression contributes to silica nanoparticle-induced pulmonary damage via p53 acetylation and cytoplasmic localisation. Toxicology. 2019;423:42-53.
183. Cai P, Wang Y, Feng N, Zou H, Gu J, Yuan Y, Liu X, Liu Z, Bian J. Polystyrene nanoplastics aggravate reproductive system damage in obese male mice by perturbation of the testis redox homeostasis. Environ Toxicol. 2023;38(12):2881-2893..
184. Abdel Aal SM, Mohammed MZ, Abdelrahman AA, Samy W, Abdelaal GMM, Deraz RH, Abdelrahman SA. Histological and biochemical evaluation of the effects of silver nanoparticles (AgNps) versus titanium dioxide nanoparticles (TiO_2NPs) on rat parotid gland. Ultrastruct Pathol. 2023 Jul 4;47(4):339-363..
185. da Silva Brito WA, Ravandeh M, Saadati F, Singer D, Dorsch AD, Schmidt A, Cecchini AL, Wende K, Bekeschus S. Sonicated polyethylene terephthalate nano- and micro-plastic-induced inflammation, oxidative stress, and autophagy in vitro. Chemosphere. 2024;355:141813.
186. Sarikhani M, Vaghefi Moghaddam S, Firouzamandi M, Hejazy M, Rahimi B, Moeini H, Alizadeh E. Harnessing rat derived model cells to assess the toxicity of TiO_2 nanoparticles. J Mater Sci Mater Med. 2022;33(5):41.
187. Hsiao TC, Han CL, Yang TT, Lee YL, Shen YF, Jheng YT, Lee CH, Chang JH, Chung KF, Kuo HP, Chuang HC. Importance of surface charge of soot nanoparticles in determining inhalation toxicity in mice. Environ Sci Pollut Res Int. 2023;30(7):18985-18997.
188. Li L, Li L, Zhou X, Yu Y, Li Z, Zuo D, Wu Y. Silver nanoparticles induce protective autophagy via Ca2+/CaMKKβ/AMPK/mTOR pathway in SH-SY5Y cells and rat brains. Nanotoxicology. 2019;13(3):369-391.
189. Zhang X, Yin H, Li Z, Zhang T, Yang Z. Nano-TiO2 induces autophagy to protect against cell death through antioxidative mechanism in podocytes. Cell Biol Toxicol. 2016;32(6):513-527.
190. Liang C, Jiang Q, Liu Z, Yang J, Zhang J, Zhang S, Xin W. Effect of Sublethal Concentrations of Metal Nanomaterials on Cell Energy Metabolism. Toxics. 2023;11(5):453.
191. Liu J, Xu F, Guo M, Gao D, Song Y. Nasal instillation of polystyrene nanoplastics induce lung injury via mitochondrial DNA release and activation of the cyclic GMP-AMP synthase-stimulator of interferon genes-signaling cascade. Sci Total Environ. 2024;948:174674.
192. Gurunathan S, Jeyaraj M, Kang MH, Kim JH. Anticancer Properties of Platinum Nanoparticles and Retinoic Acid: Combination Therapy for the Treatment of Human Neuroblastoma Cancer. Int J Mol Sci. 2020;21(18):6792.
193. Dora MF, Taha NM, Lebda MA, Hashem AE, Elfeky MS, El-Sayed YS, Jaouni SA, El-Far AH. Quercetin Attenuates Brain Oxidative Alterations Induced by Iron Oxide Nanoparticles in Rats. Int J Mol Sci. 2021;22(8):3829.

194. Liu G, Lv J, Wang Y, Sun K, Gao H, Li Y, Yao Q, Ma L, Kochshugulova G, Jiang Z. ZnO NPs induce miR-342-5p mediated ferroptosis of spermatocytes through the NF-κB pathway in mice. J Nanobiotechnology. 2024;22(1):390.
195. Zheng J, He L, Shi Q, Wang M, Ma Y, Yu W, Liu L, Yu G, Liu X, Wang B, Zhong J. In vivo and In vitro assessment of the retinal toxicity of polystyrene nanoplastics. Environ Int. 2025;198:109420.
196. Boukholda K, Elwej A, Slimen SB, Mhadhbi A, Marrekchi R, Boudawara O, Kaya B, Bouchard M, Fetoui H. Polystyrene nanoplastics exacerbate gentamicin-induced nephrotoxicity in adult rat by activating oxidative stress, inflammation and apoptosis pathways. Naunyn Schmiedebergs Arch Pharmacol. 2025; doi: 10.1007/s00210-025-03798-5
197. Chen Y, Nan Y, Xu L, Dai A, Orteg RMM, Ma M, Zeng Y, Li J. Polystyrene nanoplastics exposure induces cognitive impairment in mice via induction of oxidative stress and ERK/MAPK-mediated neuronal cuproptosis. Part Fibre Toxicol. 2025;22(1):13.
198. El Henafy HMA, Alghamdi MA, Zafrah H, Al-Zahrani NS, El Nasha EM. Impact of gold nanoparticle exposure on the development pancreas and kidney: Dose-dependent;oxidative stress; miRNA expression and Nrf2/ARE Signalling. Int Immunopharmacol. 2025;152:114409.
199. Mohamed HR, Hemdan SHA, El-Sherif AA. Y_2O_3NPs induce selective cytotoxicity, genomic instability, oxidative stress and ROS mediated mitochondrial apoptosis in human epidermoid skin A-431 Cancer cells. Sci Rep. 2025;15(1):1543.
200. Mohamed HRH, Mohamed BA, Hakeem GM, Elnawasani SH, Nagy M, Essam R, Diab A, Safwat G. Cobalt oxide nanoparticles induce cytotoxicity and excessive ROS mediated mitochondrial dysfunction and p53-independent apoptosis in melanoma cells. Sci Rep. 2025;15(1):2220.
201. Yao Y, Wang Z, Huang X, Wei T, Liu N, Zou L, Niu Y, Hu Y, Fang Q, Wang X, Qiao D, Li C, Chen M, Guan S, Xue Y, Wu T, Zhang T, Tang M. Adverse Outcome Pathway-Based Strategies to Mitigate Ag2Se Quantum Dot-Induced Neurotoxicity. ACS Nano. 2025;19(11):11029-11048.
202. Zhao M, Xie J, Zhang J, Zhao B, Zhang Y, Xue J, Zhang R, Zhang R, Wang H, Li Y, Ge W, Zhou X. Disturbance of mitochondrial dynamics led to spermatogenesis disorder in mice exposed to polystyrene micro- and nanoplastics. Environ Pollut. 2024;362:124935.
203. Zhang Y, Zhao Q, Zhao R, Lu Y, Jiang S, Tang Y. Efficacy of DHA-enriched phosphatidylserine and its underlying mechanism in alleviating polystyrene nanoplastics-induced hepatotoxicity in mice. Int Immunopharmacol. 2024;142(Pt A):113154.
204. Li S, Ma Y, Ye S, Su Y, Hu D, Xiao F. Endogenous hydrogen sulfide counteracts polystyrene nanoplastics-induced mitochondrial apoptosis and excessive autophagy via regulating Nrf2 and PGC-1α signaling pathway in mouse spermatocyte-derived GC-2spd(ts) cells. Food Chem Toxicol. 2022;164:113071..
205. Huang F, Feng Y, Wang ZA, Cao Y, Yan Q, Wang W, Feng S. Environmentally Relevant Concentrations of Commercial Titanium Dioxide Nanoparticles Induce Ferroptosis in HUVECs. Environ Toxicol. 2025; doi: 10.1002/tox.24517..
206. Li J, Gao H, Xu Z, Gao B, Zhang L, Su B, Yang S, Liu J, Liu Y, Wang X, Wang H, Lin Y, Shen H. Gestational exposure to carbon black nanoparticles triggered fetal growth restriction in mice: The mediation of inactivating autophagy-lysosomal degradation system in placental

ferroptosis. Sci Total Environ. 2025;959:178167.
207. Han M, Liang J, Wang K, Si Q, Zhu C, Zhao Y, Khan NAK, Abdullah ALB, Shau-Hwai AT, Li YM, Zhou Z, Jiang C, Liao J, Tay YJ, Qin W, Jiang Q. Integrin A5B1-mediated endocytosis of polystyrene nanoplastics: Implications for human lung disease and therapeutic targets. Sci Total Environ. 2024;953:176017
208. Gao X, Li Y, Shen J, Huang Y, Wang Y, Niu X. LC-MS untargeted metabolomics reveals metabolic disturbance and ferroptosis in MWCNTs-induced hepatotoxicity of Cyprinus carpio. Aquat Toxicol. 2024;275:107078.
209. Khayal EE, Elhadidy MG, Alnasser SM, Morsy MM, Farag AI, El-Nagdy SA. Podocyte-related biomarkers' role in evaluating renal toxic effects of silver nanoparticles with the possible ameliorative role of resveratrol in adult male albino rats. Toxicol Rep. 2024;14:101882.
210. Wang K, Hao Z, Xie J, Ma L, Zhang W, Mo J, Li L, Jin C. Nrf2-dependent hepatoprotective effect of ellagic acid in titanium dioxide nanoparticles-induced liver injury. Phytomedicine. 2024;135:156064.
211. He X, Ma L, Zhang J, Zhou B, Chen S, Tu M, Cai G, Wang T, Wang C. Dietary Rutin Ameliorates Nanoparticle Zinc Oxide-Induced Toxicity in Mice by Potentiating Antioxidant Defense Mechanisms. Nutrients. 2025;17(9):1495.
212. Saputra F, Pramata AD, Soegianto A, Hu SY. Polystyrene nanoplastics cause developmental abnormalities, oxidative damage and immune toxicity in early zebrafish development. Comp Biochem Physiol C Toxicol Pharmacol. 2025;295:110216.
213. Fan Z, Zhang Y, Fang Y, Zhong H, Wei T, Akhtar H, Zhang J, Yang M, Li Y, Zhou X, Sun Z, Wang J. Polystyrene nanoplastics induce lipophagy via the AMPK/ULK1 pathway and block lipophagic flux leading to lipid accumulation in hepatocytes. J Hazard Mater. 2024;476:134878.
214. Mohamed HRH, Elberry YA, Magdy H, Ismail M, Michael M, Eltayeb N, Safwat G. Erbium oxide nanoparticles induce potent cell death, genomic instability and ROS-mitochondrial dysfunction-mediated apoptosis in U937 lymphoma cells. Naunyn Schmiedebergs Arch Pharmacol. 2025; doi: 10.1007/s00210-025-03962-x.
215. Mohamed HRH, Essam R, Mohamed BA, Hakeem GM, Elnawasani SH, Nagy M, Safwat G, Diab A. Potent cytotoxicity and induction of ROS-mediated genomic instability, mitochondrial dysfunction, and apoptosis by Y_2O_3 NPs in Hep-G2 hepatic cancer cells. Naunyn Schmiedebergs Arch Pharmacol. 2025; doi: 10.1007/s00210-025-04051-9.
216. Wang L, Zhang L, Yun Y, Liang T, Yan C, Mao Z, Zhang J, Liu B, Zhang J, Liang T. Protective effect of astragaloside IV against zinc oxide nanoparticles induced human neuroblastoma SH-SY5Y cell death: a focus on mitochondrial quality control. Mol Cell Biochem. 2025;480(5):3079-3095.
217. Khedr MA, El-Kazaz SE, Rashed RR, Tohamy HG, Shukry M, Goma AA. Neuroprotective Effects of Alpha-Lipoic Acid Against Behavioral Toxicity, Oxidative and Inflammatory Damage Caused by Titanium Dioxide Nanoparticles. Biol Trace Elem Res. 2025; doi: 10.1007/s12011-025-04672-4.
218. Beghin M, De Groote A, Kestemont P. Single and combined effects of titanium (TiO2) and zinc (ZnO) oxide nanoparticles in the rainbow trout gill cell line RTgill-W1. Environ Sci Pollut Res Int. 2024;31(45):56523-56535.

219. Liang B, Huang X, Li Z, Huang Y, Deng Y, Chen X, Zhong Y, Yang X, Feng Y, Bai R, Fan B, Xian H, Li H, Tang S, Huang Z. Polystyrene nanoplastics trigger ferroptosis in Nrf2-deficient gut via ether phospholipid accumulation. Environ Int. 2025;197:109367.
220. Han D, Xu T, Lyu X, Li K, Xu S. Smaller-Sized Silica Nanoparticles Exacerbated Cardiomyocyte Pyroptosis by Impairing Mitophagy to Activate mtDNA-cGAS-STING Signaling. J Agric Food Chem. 2025;73(15):9359-9369.
221. Xia Y, Feng L, Lan Y, Chen X, Tang X, Xu H, Liu Y. Ghrelin relieves the reproductive damage of TiO_2 NPs in young male rats via ROS/AMPK/mTOR signaling pathway. Toxicol Appl Pharmacol. 2025:117425.
222. Das BC, Pillai D, V J RK. Sub-chronic nanoplastic toxicity in Etroplus suratensis (Pisces, Cichilidae): Insights into tissue accumulation, stress and metabolic disruption. Aquat Toxicol. 2025;285:107418..
223. Yu N, Shi J, Ma Y, Zhang Y, Guan L, Chen Z, Jia G. Absolute quantitative lipidomics reveals the disturbance of lipid metabolism induced by oral exposure of titanium dioxide nanoparticles. NanoImpact. 2025;37:100554.
224. Zheng PC, Pan XQ, Zhou YJ, Lai KP, Li R, Zhang XX. Unraveling the impact of micro- and nano-sized polymethyl methacrylate on gut microbiota and liver lipid metabolism: Insights from oral exposure studies. Environ Pollut. 2025;373:126157.
225. Mohamed HRH, Michael M, Elberry Y, Magdy H, Ismail M, Eltayeb N, Safwat G, Diab A. Induction of potent preferential cell death, severe DNA damage and p53-independent ROS-mediated mitochondrial apoptosis by $CaTiO_3$NPs in HNO-97 tongue cancer cells. Naunyn Schmiedebergs Arch Pharmacol. 2025; doi: 10.1007/s00210-025-04323-4
226. Sun D, He S, Li X, Jin B, Wu F, Liu D, Dong Z, Chen G. Toxic effects and mechanistic insights of cadmium telluride quantum dots on the homeostasis and regeneration in planarians. J Hazard Mater. 2025;486:137047.
227. Wang C, Ji X, Wang X, Song Y, Pan C, Qian M, Jin Y: The endoplasmic reticulum-mitochondrial crosstalk involved in nanoplastics and di(2-ethylhexyl) phthalate co-exposure induced the damage to mouse mammary epithelial cells. Environmental pollution (Barking, Essex : 1987) 2025, 372:126014.
228. Poinsignon L, Lefrère B, Ben Azzouz A, Chissey A, Colombel J, Djelidi R, Ferecatu I, Fournier T, Beaudeux JL, Lespes G, Zerrad-Saadi A. Exposure of the human placental primary cells to nanoplastics induces cytotoxic effects, an inflammatory response and endocrine disruption. J Hazard Mater. 2025;490:137713.
229. Yu J, Yoon JH, Park M, Lee HJ. Nobiletin-mediated autophagy mitigates nanoplastic-induced toxicity in human intestinal Caco-2 cells. FASEB J. 2025;39(6):e70452.
230. Wang L, Huang X, Dai T, Xie J, Lv QX, Hou Y, Kong L, Song Y, Liu F. The role of mitochondrial dynamics in the TiO_2 nanotube-accelerated osteogenic differentiation of MC3T3-E1 cells. Biochem Biophys Res Commun. 2021;535:33-38.
