# Supplementary material for: Toxicity Evaluation of Nano-Sized Particles by Analysis of mtDNA Content and Expression Levels of Genes Required for mtDNA Maintenance: A Meta-Analysis of Pre-Clinical Studies
Source: Antioxidants (Basel). 2026 Jul 4;15(7):848. doi: 10.3390/antiox15070848 (PMC13405982; doi:10.3390/antiox15070848)
Supplement: Supplementary file 1 [file antioxidants-15-00848-s001.zip › Table S1.pdf]

Table S1 Characteristics of included articles

No. (ref)	Author	Year	Country	Samples	No. (T/C)	Particle type	Particle size	Particle dose	Particle duration	Outcomes (assay method)
1	Sun Z	2023	China	Mouse testicular Leydig cell line (TM3)	3/3	PSNPs	20 nm	50, 100, 150 μg/mL	24 h	mtDNA content (ND1 dPCR)
2	Qi Y	2020	China	Normal human hepatocyte cell line (L-02)	3/3	SiNPs	58.04 ± 7.41 nm	12.5, 25, 50, 100 μg/mL	24 h	mtDNA content (12S rRNA dPCR), TFAM, PGC-1α, NRF1, MFN1, MFN2, OPA1, DRP1, FIS1 (WB)
3	Li Y	2020	China	Human induced pluripotent stem cell-derived cardiomyocytes	3/3	ZnONPs	40-60 nm	25, 50, 100 μM	6 h	mtDNA content (ND1 dPCR), TFAM, PGC-1α, COX4 (WB)
4	Guo C	2018	China	Human umbilical vein endothelial cells (HUVECs)	3/3	SiNPs	57.66 ± 7.30 nm	12.5, 25, 50, 100 μg/mL	24 h	mtDNA content (12S rRNA dPCR), ND1, ATP6, COX1, CYTB (RT-PCR), TFAM, PGC-1α, NRF1 (WB), MFN1, MFN2, OPA1, DRP1, FIS1 (RT-PCR, WB)
5	Yoisungnern T	2015	Thailand	Primary BDF1 mouse sperm cells	3/3	AgNPs	40 nm	0.1, 1, 10, 50 μg/mL	3 h	mtDNA content (CYTB dPCR)
6	Paesano L	2016	Italy	Human hepatocellular	9/9	CdSQDs	5 nm	3, 7, 14	4 h	mtDNA content (ND1,

				carcinoma cell line (HepG2)				μg/mL		ND4, CYTB dPCR)
7	Mohamed HRH	2023	Egypt	Male Swiss Webster mice	6/6	TiO_2NPs	60 nm	5 mg/kg	14 d	mtDNA content (12S rRNA dPCR)
8	Rivas-García L	2021	Spain	Human colorectal cancer cell line (HT-29)	8/8	FeNPs	4 nm	500 μM	48 h	mtDNA content (ND1-ND4 dPCR)
9	Mohamed HRH	2019	Egypt	Male Swiss Webster mice	6/6	$Ca(OH)_2$NPs	59.82 ± 19.62 nm	2000 mg/kg	14 d	TFAM (RT-PCR)
10	Gurunathan S	2020	Korea	Human lung adenocarcinoma cell line (A549)	3/3	PdNPs	10 nm	2.5 μM	24 h	mtDNA content (dPCR), TFAM, PGC-1α, NRF2 (RT-PCR)
11	Li L	2021	China	Primary lung cells; female C57BL/6 mice	3/3	AgNPs	100 nm	100 μg/mL; 2.5 mg/kg	24 h	mtDNA content (dPCR)
12	Xu C	2016	China	Mouse spermatocyte cell line (GC-2spd)	3/3	MWCNTs	4.5 ± 0.5 nm	0.5 μg/mL	24 h	ND1, ND2, ATP6, COX1, 2, CYTB (RT-PCR)
13	Anand AS	2023	India	Human lung adenocarcinoma cell line (A549)	5/5	ZnONPs	23 ± 8 nm	50, 100 μg/mL	4 h	MFN1, MFN2, OPA1, DRP1, FIS1 (IF)
14	Son MJ	2015	Korea	Immortalized brown mouse preadipocyte cell line	3/3	SiNPs	22.4 ± 4.3 nm; 51.4 ± 7.0 nm	50 μg/mL	24 h	PGC-1α, ND1 (RT-PCR)
15	Gurunathan S	2019	Korea	Human neuroblastoma cell line (SH-SY5Y)	3/3	AgNPs	18 nm	10 μg/mL	24 h	mtDNA content (dPCR), PGC-1α (RT-PCR)
16	Abd El-Maksoud EM	2019	Egypt	Male rats	10/10	AgNPs	54.9 nm	50 mg/kg	30 d	NRF2, PGC-1α, TFAM (RT-PCR)
17	Xu C	2019	China	Human intestinal	3/3	SeNPs	50-80 nm	4 μg/mL	12 h	mtDNA content (dPCR),

				epithelium cell line (NCM460)						NRF2 (WB)
18	Zhao T	2024	China	Yellow catfish	3/3	MnO_2NPs	55.8 ± 7.2 nm	20, 80 mg/kg	8 w	mtDNA content (ATP8 dPCR), NRF2 (RT-PCR)
19	Liu N	2022	China	Mouse leukemic monocyte/macrophage cell line (RAW264.7)	3/3	CdTeQDs	3.4 ± 0.56 nm	0.1, 0.5, 1 µM	24 h	mtDNA content (ND1, ND6, CYTB, ATP6, COX1 dPCR), PGC-1α, NRF1, TFAM, DRP1, p-DRP1, FIS1, MFN1, MFN2, MFF, OPA1 (WB)
20	Baghaee P	2024	Iran	Male Wistar rats	4/4	Y_2O_3NPs	40 nm	0.5 mg/kg	21 d	PGC-1α, TFAM, NRF1 (RT-PCR, ELISA)
21	Shen Y	2018	China	Primary rat bone marrow mesenchymal stem cells	3/3	CBNPs	14 nm	0.003, 0.03 µg/mL	7 d	mtDNA content (ND1, COX1 dPCR), PGC-1α, NRF1, TFAM, MFN2, DRP1 (RT-PCR, WB), MFN1, OPA1, FIS1 (RT-PCR), COX1 (WB)
22	Zhou H	2021	China	Male C57BL/6J mice	10/10	CuONPs	<50 nm	30, 50, 100 µg	3 d	MFN2, TFAM (WB)
23	Wang G	2023	China	Human neural stem cells	3/3	AuNPs	5 nm	10 µg/mL	24 h	DRP1, NRF1, TFAM (RT-PCR)
24	Li J	2020	China	Human hepatocellular carcinoma cell line (HepG2)	3/3	AgNPs	23.44 ± 4.92 nm	20, 40,80, 160 µg/mL	24 h	DRP1, p-DRP1, FIS1, MFN1, MFN2, PGC-1α,

										MFF, OPA1 (WB)
25	Chang X	2022	China	Male ICR mice	3/3	AgNPs	23.53 ± 4.79 nm	12, 120 mg/kg	7 d	PGC-1α (WB)
26	Yousef MI	2022	Egypt	Male Wistar rats	10/10	Al_2O_3NPs; ZnONPs	50; 100 nm	70; 100 mg/kg	75 d	PGC-1α, TFAM (RT-PCR)
27	Xu Z	2024	China	Male C57BL/6 mice; mouse myocardial cell line (HL-1)	4/4; 3/3	CBNPs	30 nm	50 μg; 1, 10, 25 μg/mL	13 w; 24 h	FIS1, MFN1, MFN2, OPA1 (WB)
28	Yousef MI	2019	Egypt	Male Wistar albino rats	10/10	Al_2O_3NPs; ZnONPs	50; 100 nm	70; 100 mg/kg	75 d	PGC-1α, TFAM (RT-PCR)
29	Chang X	2024	China	Mouse hippocampal cell line (HT22)	3/3	AgNPs	11-35 nm	2, 4, 8 μg/mL	24 h	PGC-1α (WB)
30	Nguyen KC	2020	Canada	Male BALB/c mice	8/8	CdTeQDs	5 nm	0.4, 2, 5, 6, 7, 10 mg/kg	2 h, 24 h, 3 d, 7 d	PGC-1α (ELISA)
31	Wang L	2022	China	Human neuroblastoma cell line (SH-SY5Y)	3/3	ZnONPs	50 nm	3, 6, 9, 10, 12 μg/mL	3, 6, 12, 24 h	COX2 (RT-PCR), COX4, DRP1, FIS1, MFN1, MFN2, OPA1, MFF, PGC-1α (WB)
32	Chen Y	2014	China	Human embryonic kidney cell line (HEK293T)	4/4	AgNPs	25 nm	2, 4, 8 μg/mL	24 h	PGC-1α (RT-PCR))
33	Chang X	2023	China	Mouse hippocampal cell line (HT22)	3/3	AgNPs	23.53 ± 4.79 nm	2, 4, 8 μg/mL	24 h	DRP1, p-DRP1, FIS1, MFN1, MFN2, OPA1 (WB)
34	Wang L	2024	China	Human neuroblastoma cell line (SH-SY5Y)	3/3	TiO_2NPs	10-25 nm	25, 50 μg/mL	24 h	mtDNA content (COX1 dPCR), DRP1, FIS1,

										MFN1, OPA1 (WB)
35	Arslan NP	2022	Turkey	Male BALB/c mice	10/10	AgNPs; TiO_2NPs	20 nm; <25 nm	5 mg/kg	15 d	DRP1 (RT-PCR, WB), MFN1, MFN2, OPA1 (RT-PCR)
36	Natarajan V	2015	USA	Primary rat hepatocytes	4/4	TiO_2NPs (P25, rutile, anatase)	21, 50, 50 nm	50 µg/mL	72 h	MFN1, OPA1 (RT-PCR)
37	Ma W	2020	China	Male SD rats	10/10	AgNPs	10-20 nm	200 µg	21 d	DRP1, p-DRP, FIS1, OPA1, MFN2 (WB)
38	He Y	2024	China	Porcine oocytes	100/100	PSNPs	100 nm	25, 50, 100 µg/mL	44 h	ND1, MFN1, MFN2, DRP1, OPA1 (RT-PCR)
39	Yousef MI	2021	Egypt	Male albino rats	5/5	HANPs	100 nm	300 mg/kg	45 d	TFAM (RT-PCR)
40	Gutiérrez-Carcedo P	2020	Spain	Human cervical cancer cell line (HeLa)	3/3	CeO_2NPs, AuCeO_2NPs, TPP-AuCeO_2NPs	5.2 ± 0.3 nm	20 µg/mL	24 h	NRF1 (RT-PCR, WB)
41	Hong F	2017	China	Female ICR mice	5/5	TiO_2NPs	5-6 nm	1.25, 2.5, 5 mg/kg	9 m	PGC-1α (ELISA)
42	Eldeeb GM	2024	Egypt	Male Wistar rats	10/10	HANPs	1-2 nm	280 mg/kg	45 d	PGC-1α, TFAM (RT-PCR)
43	Gurunathan S	2020	Korea	Human monocytic leukemia cell line (THP-1)	3/3	PtNPs; AgNPs	30 nm	25, 50, 75, 100, 125, 150 µg/mL; 50 µg/mL	24 h	,mtDNA content (dPCR), PGC-1α (RT-PCR)
44	Skóra B	2024	Poland	Normal mouse spermatogonia cell line (GC-1 spg); spermatocyte	3/3	AgNPs	5 nm	1 µg/mL	24, 48 h	PGC-1α, NRF2 (WB)

				cell line [GC-2 spd(ts)]						
45	Zhao X	2021	China	Human lung bronchial epithelial cell line (BEAS-2B)	3/3	SiNPs	57.7 ± 7.3 nm	2, 10, 50 μg/mL	24 h	NRF2 (RT-PCR)
46	Wei S	2019	China	Human bronchial epithelial cells (HBE)	3/3	ZnONPs	37.3 nm	7.5 μg/mL	6 h	NRF2 (RT-PCR)
47	Lin C	2016	China	Human lung adenocarcinoma cell line (A549)	3/3	SiO_2NPs	41.32 ± 3.19 nm	50 μg/mL	12 h	NRF2 (RT-PCR)
48	Nguyen KC	2013	Canada	Human hepatocellular carcinoma cell line (HepG2)	6/6	CdTeQDs	5 nm	10 μg/mL	24 h	NRF2 (ELISA)
49	Nguyen KC	2015	Canada	Human hepatocellular carcinoma cell line (HepG2)	6/6	CdTeQDs	5 nm	10 μg/mL	24 h	PGC-1α (ELISA)
50	Dey S	2022	India	Human breast carcinoma cell line (MCF-7)	3/3	AgNPs	9 nm	25 μM	6 h	p-DRP1 (WB)
51	Zhao X	2022	China	Human cardiomyocyte cell line (AC16)		SiNPs	57.70 ± 7.30 nm	12.5, 25, 50, 100 μg/mL	24 h	ND1, COX1, CYTB, ATP6 (RT-PCR), FIS1, MFN1, MFN2, DRP1, p-DRP1, OPA1 (WB)
52	Li J	2021	China	ICR male mice; human hepatocellular carcinoma cell line (HepG2)	3/3	AgNPs	23.44 ± 4.92 nm	12, 120 mg/kg; 160 μg/mL	7 d; 48 h	DRP1, p-DRP1, MFN1, MFN2, FIS1, OPA1(WB)
53	Zheng H	2024	China	Mouse spermatogonia cell line (GC-1)	3/3	NiNPs	30-100 nm	25, 50, 100 μg/mL	24 h	DRP1, MFN1, MFN2, FIS1, OPA1 (WB)
54	Bittner A	2019	Switzerlan d	Rat brain capillary endothelial cell line	3/3	PCLNPs	90 nm	24.9, 2.49 x 10^{-3}, 2.49 x	24 h	DRP1, OPA1, MFN2 (WB)

				(rBCEC4)				10^{-7} μg/mL		
55	Wilson CL	2015	USA	Primary rat cortical astrocytes	3/3	TiO_2NPs (P25, rutile, anatase)	50 nm	25, 100 μg/mL	24 h	MFN1, DRP1, MFN2 (RT-PCR)
56	Li Y	2022	China	Human bronchial epithelial cell line (16HBE)	3/3	SiNPs	58 nm	12.5, 25, 50, 100 μg/mL	24 h	FIS1, MFN1, DRP1, p-DRP, MFN2, OPA1 (WB)
57	Fan Y	2022	China	Human umbilical vein endothelial cell line (EA.hy926); C57BL/6 J male mice	3/3	CuONPs	50 nm	5, 7.5, 10, 15 μg/mL; 2.5, 5 mg/kg	12 h; 3 d	FIS1, p-DRP1/DRP1 (WB)
58	Ma W	2020	China	Male SD rats	10/10	AgNPs	20 nm	200 μg	7 d	FIS1, DRP1, p-DRP1 (WB)
59	Kong L	2021	China	Male BALB/c mice	10/10	NiNPs	90 nm	5, 15, 45 mg/kg	28 d	DRP1 (WB)
60	Fu Y	2022	China	Human umbilical vein endothelial cells (HUVECs)	3/3	PSNPs; NH2-PSNPs	50 nm	10, 20 μg/mL	24 h	COX1, MFN1, MFN2, DRP1, OPA1, FIS1, TFAM, ATP6, (RT-PCR)
61	Liu Z	2021	China	Human umbilical vein endothelial cells (HUVECs)	3/3	γ-Fe_2O_3NPs; Fe_3O_4NPs	55.50 ± 15.51 nm; 14.05 ± 3.56 nm	50, 200 μg/mL	24 h	OPA1, DRP1 (RT-PCR)
62	Tian T	2024	China	Mouse hippocampal cell line (HT22)	3/3	SiNPs	57.35 ± 9.46 nm	6.25, 12.5, 25, 50, 100 μg/mL	24 h	OPA1, DRP1, p-DRP, MFN2 (WB)
63	Ko WC	2020	China	Rat vascular smooth muscle cells (VSMCs)	3/3	AuNPs	3.38 ± 0.974 nm	2 μg/mL	0.5, 1, 2, 4, 8, 16	NRF2, p-NRF2 (WB)

									h	
64	Zhang X	2022	China	Human lung bronchial epithelial cell line (BEAS-2B); male ICR mice	6/6	AgNPs	38.17 ± 7.90 nm	320 μg/mL; 1 mg/kg	24 h; 3 d	NRF2 (WB)
65	Liu N	2022	China	Mouse leukemic monocyte/macrophage cell line (RAW264.7)	3/3	CdTeQDs	3.4 ± 0.56 nm	0.1, 0.5, 1 μM	24 h	NRF2 (WB)
66	Vineetha VP	2020	India	Nile tilapia	60/60	TiO_2NPs	< 25 nm	10 μg/mL	14 d	NRF2 (WB)
67	Zhai S	2024	China	Mouse leukemic monocyte/macrophage cell line (RAW264.7)	3/3	AuNPs	5.8 ± 0.4 nm; 6.1 ± 0.5 nm; 7.8 ± 1.2 nm; 9.6 ± 2.3 nm; 10.2 ± 2.5 nm	136 nM	24 h	NRF2 (WB)
68	Yin Y	2021	China	Human keratinocyte cell line (HaCaT)	3/3	ZnONPs	50 nm	25, 50, 100, 200, 300 μM	6 h	NRF2 (WB)
69	Li N	2022	China	Human umbilical vein endothelial cell line (HUVECs); male C57BL/6-J mice	3/3	CuONPs	< 50 nm	5, 10, 15, 20, 30 μg/mL; 5 mg/kg	3, 6, 9, 12 h; 3d	NRF2 (WB)
70	Mahmoud AM	2019	Egypt	Male Wistar rats	6/6	SiNPs	50 nm	25, 50, 100, 200 mg/kg	30 d	NRF2 (WB)
71	Sun X	2017	China	Human umbilical vein endothelial cells (HUVECs)	3/3	AgNPs	20-50 nm	0.5, 1, 2 μg/mL	24 h	NRF2 (WB, RT-PCR)
72	Hong F	2020	China	Male ICR mice	5/5	Anatase TiO_2NPs	5-7 nm	1.25, 2.5, 5 mg/kg	4 m	NRF2 (WB)

73	Wang M	2023	China	Human keratinocyte cell line (HaCaT)	3/3	ZnONPs	50 nm	40 μg/mL	6 h	NRF2 (WB)
74	Wen Y	2024	China	Male Kunming mice; alpha mouse liver-12 cell line (AML-12)	6/6	PSNPs	20 nm	0.05, 0.5, 5 mg/kg; 50, 100, 150 μg/mL	28 d; 24 h	NRF2, p-NRF2 (WB)
75	Maher AM	2024	China	Male SD rats	3/3	ZnONPs	50 nm	5 mg/kg	4 w	NRF2 (WB)
76	Chen T	2023	China	Mouse leukemic monocyte/macrophage cell line (RAW264.7)	3/3	ZnONPs	47.53 nm	10, 20, 40 μg/mL	24 h	NRF2 (WB)
77	Liu W	2017	China	Human lung adenocarcinoma cell line (A549)	3/3	SiNPs	10-20 nm	15 μg/mL	24 h	NRF2 (WB)
78	Shi Z	2015	China	Human hepatocellular carcinoma cell line (HepG2)	3/3	TiO_2NPs	10-25 nm	0.1, 1, 10 μg/mL	24 h	NRF2 (RT-PCR ,WB)
79	Zhang L	2021	China	Human umbilical vein endothelial cells (HUVECs); male C57BL/6J mice	3/3	ZnONPs	50 nm	5, 10, 15, 20 μg/mL; 12 μg	3, 6, 9, 12, 24 h; 3 d	NRF2 (RT-PCR ,WB)
80	Guo C	2015	China	Human umbilical vein endothelial cells (HUVECs)	3/3	SiNPs	58 nm	12.5, 25, 50, 100 μg/mL	24 h	NRF2 (RT-PCR)
81	Elblehi SS	2022	Egypt	Wistar Albino rats	7/7	AgNPs	10 nm	50 mg/kg	30 d	NRF2 (RT-PCR)
82	Abdou KH	2019	Egypt	Male albino rats	10/10	TiO_2NPs	63.8 nm	500 mg/kg	60 d	NRF2 (RT-PCR)
83	Kang SJ	2012	Korea	Human ovarian carcinoma cell line (SK-OV3)	3/3	AgNPs	7.5 ± 2.5 nm	10 μg/mL	24 h	NRF2 (RT-PCR)
84	Zhang H	2017	USA	Human acute monocytic	3/3	Si-FeNPs	60 ± 12 nm	20 μg/mL	0.5, 1, 3,	NRF2 (WB)

				leukemia cell line (THP-1)					4, 6, 9, 12, 18 h	
85	Fu X	2024	China	Mouse spermatocyte cell line (GC-2 spd); male ICR mice	3/3	PSNPs	50 nm; 90 nm	12.5, 25 μg/mL; 15 mg/kg	24 h; 60 d	NRF2 (WB)
86	Guo M	2023	China	Human hepatocellular carcinoma cell line (HepG2), normal human hepatocyte cell line (L-02)	3/3	AgNPs	21.31 ± 4.00 nm	20, 40, 80, 160 μg/mL	48 h	NRF2 (WB)
87	Abdelrahman SA	2022	Egypt	Male Wistar albino rats	10/10	AgNPs	<100 nm	500 mg/kg	28 d	NRF2, SIRT1 (RT-PCR)
88	Teng J	2024	China	Zebrafish	40/40	ZnONPs	<50 nm	20 μg/mL	24 h	NRF2 (RT-PCR)
89	Ma Y	2024	China	Male SD rats; human umbilical vein endothelial cells (HUVECs)	3/3	AgNPs	20 nm	200 μg; 0.3 μg/mL	2 w; 24 h	NRF2 (IF, RT-PCR, WB)
90	Cui G	2021	China	Rat myocardial cell line (H9c2)	3/3	SiNPs	59.65 ± 5.34 nm	25, 50, 100, 200 μg/mL	24 h	NRF2 (WB)
91	Huang J	2023	China	Human ovarian granulosa cell line (COV434)	3/3	PSNPs	50 nm	150 μg/mL	12, 24, 48 h	NRF2 (WB)
92	Zheng F	2019	China	Male Wistar rats	5/5	CoNPs	96 nm	2, 4, 8 mg/kg	20 d	NRF2 (WB)
93	Hashim AR	2024	Egypt	Male Wistar albino rats	10/10	CuONPs	20 nm	0.5 mg/kg	28 d	NRF2 (RT-PCR)
94	Wu Z	2018	Singapore	Human dermal keratinocyte cell line (HaCaTs)	3/3	ZnONPs	<100 nm	0.5 μg/mL	4 h	NRF2 (IF)
95	Wang Y	2022	China	Human lung adenocarcinoma cell line (A549)	3/3	SiNPs	50 nm	20 $\mu g/cm^2$	24 h	NRF2 (WB)

96	Yang J	2021	China	Mouse primary corneal endothelial cells	3/3	TiO_2NPs	20 nm	25 µg/mL	24 h	NRF2 (RT-PCR, WB)
97	Salama B	2023	Egypt	Male Wistar albino rats	7/7	AgNPs	< 40 nm	50 mg/kg	28 d	NRF2 (RT-PCR)
98	Fonseca E	2023	Portugal	Turbots	3/3	TiO_2NPs	5, 25 nm	1.5 mg/kg	14, 28 d	NRF2 (RT-PCR)
99	Hou J	2021	China	Mouse mammary epithelial cell line (HC11)	3/3	AgNPs	<100 nm	12.5, 25, 50 µg/mL	24 h	NRF2, p-AMPK (WB)
100	Kandeil MA	2020	Egypt	Male albino rats	20/20	TiO_2NPs	90 nm	500 mg/kg	14 d	NRF2 (WB)
101	Alshammari GM	2023	Saudi Arabia	Male Wistar rats	8/8	AuNPs	10 nm	200 µL	5 w	NRF2 (RT-PCR, ELISA, WB)
102	Nemmar A	2019	UAE	Male Wistar rats	6/6	CeO_2NPs	20 nm	1 mg/kg	24 h	NRF2 (IHC)
103	Li X	2022	China	Primary human epidermal keratinocytes (HEK)	3/3	AuNPs	50 nm	25, 75 µg/mL	24 h	NRF2 (RT-PCR)
104	Li T	2022	China	Human trophoblastic cell line (HTR-8/SVneo)	4/4	NiNPs	< 100 nm	2.5, 5, 7.5, 10, 12.5 $\mu g/cm^2$	24 h	mtDNA content (ND1 dPCR), NRF2 (WB)
105	Zhou D	2022	China	Human lung adenocarcinoma cell line (A549)	3/3	TiO_2NPs	50 nm	20 $\mu g/cm^2$	24 h	NRF2 (WB)
106	Zou L	2021	China	Human ovarian granulosa tumor cell line (COV434)	3/3	CuNPs	100 nm	150 µg/mL	12 h	NRF2 (WB)
107	Korakaki E	2023	Greece	Human acute monocytic leukemia cell line (THP-1)	3/3	γ-Fe_2O_3NPs-Fe_3O4NPs	4 nm	0.1, 1, 5, 10 µg/mL	24 h	NRF2 (WB)
108	Li X	2020	China	Mice	10/10	CdSe/ZnSQDs	10 nm	50 mg/kg	14 d	NRF2 (RT-PCR, ELISA)
109	Afshari-Kaveh M	2021	Iran	Male Wistar rats	6/6	TiO_2NPs	20 nm	300 mg/kg	14 d	NRF2 (RT-PCR)
110	Rajkumar KS	2022	India	Common carp	30/30	ZnONPs	35 nm	0.382, 0.573,	28 d	NRF2 (RT-PCR)

								1.146 μg/mL		
111	Magaye R	2016	China	Human lung adenocarcinoma cell line (A549); male SD rats	3/3; 5/5	NiNPs	40.50 ± 18.6 nm	1, 5, 10, 15, 25 μg/cm^2	24 h	NRF2 (WB)
112	Noshy PA	2023	Egypt	Male Wistar rats	6/6	AgNPs; ZnNPs	< 50 nm; < 100 nm	50 mg/kg; 30 mg/kg	12 w	NRF2 (RT-PCR)
113	Zhu B	2019	China	Zebrafish	120/120	SiNPs	13.56 ± 0.34 nm; 27.79 ± 0.46 nm	25, 50, 100 μg/mL	5 d	NRF2 (RT-PCR)
114	Zhou F	2019	China	Human umbilical vein endothelial cells (HUVECs)	3/3	SiNPs	S50 (50 ± 8.7 nm); S25 (25 ± 4.3 nm); S10 (10 ± 1.5 nm)	25 μg/mL	24 h	NRF2 (WB)
115	Liao F	2019	China	Human umbilical vein endothelial cells (HUVECs)	3/3	TiO_2NPs	T50 (50 ± 7.6 nm); T30 (30 ± 5.1 nm); T10 (10 ± 2.3 nm)	25 μg/mL	24 h	NRF2 (WB)
116	Mytych J	2016	Poland	Normal diploid human facial skin fibroblast cells	3/3	ND; SiO_2NPs	< 10 nm; 12 nm	0.5 μg/mL	48 h	NRF2, SIRT1 (IF)
117	Yasin NAE	2022	Egypt	Male albino rats	10/10	PSNPs	25 nm	3, 10 mg/kg	5 w	NRF2 (RT-PCR)
118	González-Fernández C	2021	Spain	Gilthead seabream neuronal-like stem cell line (SaB-1)	3/3	PSNPs-NH_2; PSNPs-COO; PSNPs-plain	52.3 ± 0.1 nm ; 55.6 ± 0.2 nm; 54.8 ± 0.1 nm	1, 12 μg/mL	24 h	NRF2 (RT-PCR)
119	Tian J	2019	China	Zebrafish	10/10	MPA-CdTeQDs; MPA-CdSeCdTe QDs	4.15 ± 0.93 nm; 8.12 ± 1.45 nm	0.05, 0.1, 0.5 μg/mL	24, 48 h	NRF2 (RT-PCR)

120	Krishnasamy Sekar R	2023	India	Common carp	3/3	AgNPs	8-50 nm	0.184, 0.276, 0.552 µg/mL	28 d	NRF2 (RT-PCR)
121	Skočaj M	2020	Slovenia	Rat skeletal muscle cell line (L6)	3/3	SiO_2NPs,TiO_2NPs, PAANPs	47 ± 2 nm; 64 ± 22 nm	50 µg/mL	10 d	NRF2 (RT-PCR)
122	Niu L	2017	China	Female and male Kunming mice	8/8	TiO_2NPs	10-25 nm	2000 mg/kg	7 d	NRF2 (WB)
123	Santacruz-Márquez R	2023	Mexico	Female CD-1 mice antral follicles	36/36	ZnONPs	13-89 nm	5, 10, 15 µg/mL	4 d	NRF2 (RT-PCR)
124	Berg JM	2013	USA	Human mesothelium-derived epithelial cell line (MeT-5A)	3/3	SiO_2NPs	33.5 ± 7.73 nm	75 µg/mL	0.5, 1, 2, 4, 8, 12, 24, 48 h	NRF2 (RT-PCR)
125	Ferraro SA	2020	Argentina	Human neuroblastoma cell line (SH-SY5Y)	3/3	TiO_2NPs	5 nm	5, 10, 50, 100 µg/mL	24 h	NRF2 (ICC)
126	Sun Q	2012	China	Female CD-1 ICR mice	5/5	TiO_2NPs	5-6 nm	10 mg/kg	15, 30, 45, 60, 75, 90 d	NRF2 (RT-PCR, ELISA)
127	Ucar A	2022	Turkey	Rainbow trout	7/7	Fe_3O_4NPs	30 nm	0.013 mL/L	48, 96 h	NRF2 (ELISA)
128	Guo C	2016	China	Human umbilical vein endothelial cells (HUVECs)	3/3	SiNPs	57.66 ± 7.30 nm	50 µg/mL	24 h	NRF2 (RT-PCR)
129	Li Y	2024	China	Primary bovine intestinal epithelial cells (BIECs)	3/3	ZnONPs	18.29 nm	0.8 µg/mL	6 h	NRF2 (RT-PCR)
130	Kaur K	2024	India	Zebrafish	12/12	TiO_2NPs	< 100 nm	10 µg/mL	7 d	NRF2 (IHC)
131	Qiao L	2020	China	Male C57BL/6 mice	10/10	SeNPs	50-80 nm	1 mg/kg	14 d	mtDNA content (COX2 dPCR), NRF2 (WB)
132	Hassanen EI	2020	Egypt	Chicks	7/7	AuNPs	25 ± 5 nm	5, 15 µg/mL	36 d	NRF2 (RT-PCR)

133	Liu W	2024	China	Female C57BL/6 mice	3/3	SiNPs	5-20 nm	3, 10 mg/kg	8 w	NRF2 (RT-PCR)
134	Wang H	2018	China	Human hepatocyte cell line (L-02), hepatocellular carcinoma cell line (QGY)	3/3	TiO_2NPs	21 nm	40, 80 µg/mL	72 h	NRF2 (RT-PCR)
135	Eom HJ	2009	Korea	Human bronchial epithelial cell line (Beas-2B)	3/3	Fumed SiNPs; porous SiNPs	7 nm; 5-15 nm	40 µg/mL	24 h	NRF2 (WB)
136	Liu X	2019	China	Female and male C57BL/6J mice	3/3	CBNPs	14 nm	21, 103, 515 µg	49 d	NRF2 (WB)
137	Wang J	2011	China	Female CD-1 (ICR) mice	5/5	TiO_2NPs	7.5 nm	5, 10, 150 mg/kg	30 d	NRF2 (RT-PCR, ELISA)
138	Eom HJ	2011	Korea	Human bronchial epithelial cell line (Beas-2B)	3/3	SiO_2NPs	20-40 nm	1 µg/mL	24 h	NRF2 (WB)
139	Liang Y	2024	China	Male C57BL/6 mice; Human hepatocyte cell line (L-02)	3/3	PSNPs	80 nm	100, 150 mg/kg; 100, 200, 300, 400, 500 µg/mL	30 d; 24 h	NRF2 (WB)
140	Dhupal M	2018	Korea	Mouse leukemic monocyte/macrophage cell line (RAW264.7)	3/3	TiO_2NPs	21 ± 3 nm	2.5, 5, 10 µg/mL	6 h	NRF2 (WB)
141	Brown DM	2014	UK	Male SD rats	3/3	NH2-, plain SiNPs	50	30 µg	24 h	NRF2 (IF)
142	da Rocha AM	2013	Brazil	Zebrafish	4/4	SWCNT, fullerenol	< 100 nm	30 mg/kg	48 h	NRF2 (RT-PCR)
143	Eom HJ	2009	Korea	Human bronchial epithelial cells Beas-2B	3/3	CeO_2 NPs	15, 30, 45 nm	1 µg/mL	24 h	NRF2 (WB)

144	Ze Y	2013	China	Male CD-1 ICR mice	5/5	TiO_2NPs	5-6 nm	2.5, 5, 10 mg/kg	90 d	NRF2 (RT-PCR, ELISA)
145	Kluknavsky M	2023	Slovakia	Male WKY rats	6/6	Fe_3O_4NPs	28-32 nm	1 mg/kg	1.6 h	NRF2 (RT-PCR)
146	Eid A	2023	Egypt	Male albino rats	20/20	TiO_2NPs	27 nm	200 mg/kg	8 w	NRF2 (RT-PCR)
147	Alghriany AAI	2022	Egypt	Male mice	15/12	Al_2O_3NPs	38.31 ± 2.45 nm	6 mg/kg	4 w	NRF2 (WB)
148	Wei S	2024	China	Mussel (Mytilus coruscus)	3/3	TiO_2NPs	25 nm	0.1 μg/mL	14 d	NRF2 (RT-PCR)
149	Voicu SN	2019	Romania	Human lung fibroblast cell line (MRC-5)	3/3	SiO_2NPs	< 10 nm	62.5 μg/mL	24, 48, 72 h	NRF2 (WB)
150	Micurova A	2021	Slovakia	Male WKY rats	7/7	Fe_3O_4NPs	45 nm	2 mg/kg	2 d	NRF2 (RT-PCR)
151	Abd-Eltawab Tammam A	2022	Saudi Arabia	Male Wistar rats	10/10	NiONPs	42 nm	100 mg/kg	8 w	NRF2 (RT-PCR)
152	Dou X	2024	China	Male C57BL/6 mice	3/3	SeNPs	50-80 nm	1 mg/kg	4 w	FIS1 (WB)
153	Kuang H	2021	China	Male and female Kunming mice	5/5	ZnONPs	30 nm	100 mg/kg	3 d	NRF2 (RT-PCR)
154	Yin X	2022	China	Human monocytic cell line (THP-1)	3/3	ZnONPs	30 nm	10, 20, 40 μg/mL	24 h	DRP1 (RT-PCR, WB)
155	Li X	2022	Korea	Human corneal epithelial cells (HCECs), human conjunctival epithelial cells (HCjECs)	3/3	TiO_2-, CB-, ZnO-, SiO_2-NPs	< 100 nm; 25 nm; < 100 nm; 10-20 nm	100 μg/mL	6 h	SIRT1 (WB)
156	Yang S	2024	China	Male C57BL/6 mice	6/6	PSNPs	40 nm	16, 40, 100 μg	1 w, 1, 3 m	DRP1, MFN1, MFN2 (WB)
157	Khan AA	2021	Saudi Arabia	Rat heart-tissue derived embryonic cardiac myoblast cell line (H9c2)	3/3	AgNPs	4-12 nm	3.5 μg/mL	24, 48 h	p-AMPK (WB)

158	Sapienza S	2022	Italy	Mouse motor-neuron-like cell line (NSC-34)	3/3	nPM	< 20 nm; < 100 nm	0.71 μg/mL; 2.86 μg/mL	48 h	p-AMPK (WB)
159	Li Y	2023	China	Human hepatocellular carcinoma cell line (HepG2)	3/3	PSNPs	21.5 ± 2.7 nm	6.25, 12.5, 25, 50 μg/mL	24 h	DRP1, p-DRP1, MFN2, MFN1, OPA1, PGC-1α, FIS1 (WB)
160	Li X	2024	Korea	Human retinal pigment epithelial cell line (ARPE-19)	3/3	PSNPs	50 nm	200 μg/mL	48 h	DRP1, FIS1 (WB)
161	Liang Y	2024	China	Male C57BL/6 mice; mouse spermatogonia cell line (GC-1)	3/3	PSNPs	80 nm	10, 40 mg/kg; 5, 10, 20 μg/mL	60 d; 5 d	SIRT1 (WB)
162	Bai H	2024	China	Mouse hippocampal neuron cell line (HT22); male C57BL/6 mice	3/3	PSNPs	100 nm	50 μg/mL; 40 mg/kg	12 h; 15 w	SIRT1 (WB)
163	Shiwakoti S	2022	Korea	Porcine coronary artery endothelial cells (PCAECs)	3/3	PSNPs	25 nm	0.1, 1, 10 μg/mL	24 h	SIRT1 (WB)
164	Shen J	2022	China	Human colorectal cancer cell line (HCT116)	3/3	GO	0.67 nm	10, 50 μg/mL	10 d	p-AMPK/AMPK (WB)
165	Tang Y	2023	China	Male C57BL/6J mice	8/8	PSNPs	100 nm	25 mg/kg	6 w	SIRT1, AMPK (IHC)
166	Chen W	2021	China	Human colorectal adenocarcinoma cell line (Caco2)	3/3	PSNPs	80 nm	100 μg/mL	24 h	SIRT1 (WB)
167	Huang Y	2023	China	Human neuroblastoma cell line (SH-SY5Y)	3/3; 5/5	PSNPs	50.7 nm	0.5, 5, 50, 500 μg/mL	48 h	COX2, p-AMPK/AMPK (WB)
168	Zhang C	2023	China	Zebrafish	5/5	PSNPs	100 nm	1 μg/mL	30 d	mtDNA content (ND1

										dPCR), PGC-1α, MFN1, OPA1, DRP1, FIS1, MFF (RT-PCR)
169	Fernandes AL	2018	Brazil	Zebrafish	3/3	Graphene	100 nm	0.05, 0.5 μg	48 h	NRF2 (RT-PCR)
170	Brown DM	2010	UK	Human monocytic leukemia cell line (THP-1)	3/3	MWCNTs; CBNPs	20 nm; 14 nm	62.5 μg/mL	4 h	NRF2 (RT-PCR)
171	Pelka J	2013	Austria	Human colon carcinoma cell line (HT29)	3/3	SWCNTs	1-1.4 nm	0.00001, 0.00005, 0.001, 0.005, 0.01, 0.05, 0.1, 0.2 μg/mL	1 h	NRF2 (WB)
172	Stanca L	2023	Romania	Mouse leukemic monocyte/macrophage cell line (RAW264.7)	3/3	Si/SiO_2QDs	6-8 nm	5, 15 μg/mL	6, 12, 24 h	NRF2 (WB)
173	Guo M	2024	China	Human hepatocellular carcinoma cell line (HepG2)	3/3	PSNPs	20 nm	50 μg/mL	12 h	NRF2 (WB)
174	Sun R	2024	China	Human normal gastric epithelial cell line (GES-1)	3/3	PSNPs	50 nm	20, 40, 80 μg/mL	24 h	NRF2, MFN1, MFN2, DRP1, FIS1 (WB)
175	Wang W	2024	China	Human ovarian granulosa cell line (KGN)	3/3	PSNPs	25 nm	150 μg/mL	24 h	NRF2 (WB)
176	Wu Y	2024	China	Male C57BL/6 mice; mouse auditory cell line (HEI-OC1)	3/3	PSNPs	21.9 ± 0.6 nm	5, 25 mg/kg; 50, 100, 200, 500, 214.5 μg/mL	8 w; 24 h	NRF2 (WB)

177	Ling X	2022	China	Zebrafish	4/4	PSNPs	83.06 ± 15.3 nm	0.1 μg/mL	90 d	NRF2 (RT-PCR)
178	Ijaz MU	2024	Pakistan	Albino rats	12/12	PSNPs	100 nm	50 μg/kg	30 d	NRF2 (RT-PCR)
179	Liu L	2022	China	Male BALB/c mice; mouse spermatogonia cell line (GC-1)	8/8; 3/3	NiNPs	90 nm	5, 15, 45 mg/kg; 25, 50, 100 μg/mL	28 d; 24 h	DRP1 (WB)
180	Feng L	2021	China	Human neuroblastoma cell line (SH-SY5Y)	3/3	GO	1 nm	40, 60, 80, 100 μg/mL	24 h	COX4 (ELISA), p-AMPK/AMPK (WB)
181	Duan WX	2015	China	Human bronchial epithelial cell line (BEAS-2B)	3/3	NiONPs	< 50 nm	5, 10, 20 μg/cm^2	24, 48 h	SIRT1 (RT-PCR, WB)
182	Zhao X	2019	China	Human bronchial epithelial cell line (BEAS-2B); male ICR mice	3/3	SiNPs	38.6 nm	625, 12.5, 25, 50 μg/mL; 5 mg/kg	1, 3, 6, 12 h; 1w	SIRT1 (WB, RT-PCR)
183	Cai P	2023	China	Male BALB/c mice	6/6	PSNPs	80-100 nm	1000 μg	28 d	NRF2 (WB)
184	Abdel Aal SM	2023	Egypt	Male albino rats	9/9	AgNPs; TiO_2NPs	100 nm	100 mg/kg; 150 mg/kg	4, 8 w	PGC1-α (RT-PCR)
185	da Silva Brito WA	2024	Brazil	Human lung carcinoma cell line (A549); human keratinocytes (HaCaT)	3/3	PSNPs-NH2; PET-NPs	54.46 ± 0.289 nm	1, 10, 100 μg/mL	24 h	NRF2 (WB)
186	Sarikhani M	2022	Iran	Rat bone marrow mesenchymal stem cells (rBMSCs); rat adipose mesenchymal stem cells (rATSC)	3/3	TiO_2NPs	77.91 nm	200; 100 μg/mL	48 h	SIRT1 (RT-PCR)
187	Hsiao TC	2023	China	Female BALB/c mice	5/5	SNPs	69.58 - 70.02 nm	1.56-3.53×	7 d	SIRT1 (WB)

							for the neutral case; 70.60-72.38 nm for the positively charged case; 70.19-71.78 nm for the negatively charged case	10^4 cm^3; 7.29 × 10^3 -3.62 × 10^4 cm^3; 9.66 × 10^3 -5.27 × 10^4 cm^3		
188	Li L	2019	China	Human neuroblastoma cell line (SH-SY5Y)	3/3	AgNPs	31.1 ± 6.8 nm	12.5 μg/mL	6, 12, 24, 48 h	p-AMPK (WB)
189	Zhang X	2016	China	Mouse podocyte cells (MPCs)	3/3	TiO_2NPs	21 nm	10, 100 μg/cm^2	24 h	P-AMPK/AMPK (WB)
190	Liang C	2023	China	Human proximal tubule epithelial cell line (HK-2)	3/3	ZnONPs; CuONPs; AgNPs; AgNWs	10-50 nm; 30 nm; 30 nm	10 μg/mL	24 h	Mt D-loop (RT-PCR), COX2 (WB)
191	Liu J	2024	China	male C57BL/6 mice	3/3	PSNPs	90 nm	5, 10 mg/kg	7 d	mtDNA content
194192	Gurunathan S	2020	Korea	Human neuroblastoma cell line (SH-SY5Y)	3/3	PtNPs	25 nm	25 μg/mL	24 h	mtDNA content (dPCR), PGC1-α (RT-PCR)
193	Dora MF	2021	Egypt	Male albino rats	5/5	FeONPs	16.34-22.88 nm	50 mg/kg	30 d	PGC-1α, TFAM (RT-PCR)
194	Liu G	2024	China	Mouse spermatocyte cell line (GC-2spd)	3/3	ZnONPs	30 ± 5 nm	1 μg/mL	24 h	CYTB, COX2 (RT-PCR)
195	Zheng J	2025	China	Male C57BL/6 mice; human retinal pigment epithelial cell	3/3	PSNPs	72.90 ± 18.46 nm	10, 50 mg/kg; 50, 200	28 d; 24 h	NRF2 (WB)

				line (ARPE-19)				μg/mL		
196	Boukholda K	2025	Tunisia	Male Wistar rats	3/3	PSNPs	50 nm	2.5 mg/kg	15 d	NRF2 (RT-PCR)
197	Chen Y	2025	China	Male C57BL/6 mice	3/3	PSNPs	60 nm	12.5 mg/kg	30 d	NRF2 (IHC)
198	El Henafy HMA	2025	Saudi Arabia	Offspring of female pregnant Wistar rats	10/10	AuNPs	50 nm	5, 10, 15, 20 mg/kg	60 d	NRF2 (ELISA)
199	Mohamed HRH	2025	Egypt	Human epidermoid skin cancer cell line (A-431)	3/3	Y_2O_3NPs	14.15 nm	7.47, 14.94, 29.89 μg/mL	72 h	ND3 (RT-PCR)
200	Mohamed HRH	2025	Egypt	Human melanoma cell line (A-375)	3/3	Co_3O_4NPs	20.08 nm	303.8 μg/mL	72 h	ND3 (RT-PCR)
201	Yao Y	2025	China	Mouse microglial cell line (BV2)	3/3	Ag_2SeQDs	2.5 ± 0.3 nm	2, 4, 8 μg/mL	24 h	NRF2, DRP1, p-DRP1, FIS1 (WB)
202	Zhao M	2024	China	Male Balb/c mice; mouse spermatocyte cell line (GC-2spd)	5/5	PSNPs	67.19 ± 6.437 nm	10 mg/kg; 25 μg/mL	28 d; 24 h	FIS1, MFN1/2, OPA1 (WB)
203	Zhang Y	2024	China	Male C57BL/6J mice	3/3	PSNPs	100 nm	25 mg/kg	6 w	SIRT1, AMPK (IHC)
204	Li S	2022	China	Mouse spermatocyte cell line [GC-2spd(ts)]	3/3	PSNPs	80 nm	400 μg/mL	24 h	NRF2, PGC-1α (IF, WB)
205	Huang F	2025	China	Human umbilical vein endothelial cells (HUVECs)	3/3	TiO_2NPs	30 ± 10 nm	3, 12, 48 μg/mL	24 h	NRF2, p-NRF2 (WB)
206	Li J	2025	China	Female and male C57BL/6 mice; human placental trophoblast cell line (HTR-8/SVneo)	5/5	CBNPs	30	200 μg	18 d	DRP1, p-DRP1, FIS1, MFN1, MFN 2, OPA1 (WB), mtDNA content (ND1/5 dPCR)
207	Han M	2024	China	Human bronchial epithelial cell line (BEAS-2B)	3/3	PSNPs	80 nm	100 μg/mL	24 h	NRF2 (WB)

208	Gao X	2024	China	Common carp	15/15	MWCNTs	4-6 nm	2.5 μg/mL	28 d	NRF2 (RT-PCR)
209	Khayal EE	2024	Egypt	Male Wistar albino rats	3/3	AgNPs	< 100 nm	300 mg/kg	28 d	SIRT1 (ELISA)
210	Wang K	2024	China	Primary mouse hepatocytes and cell line (L02)	3/3	TiO_2NPs	21 nm	10 μg/mL	24 h	NRF2 (WB)
211	He X	2025	China	Male ICR mice	8/8	ZnONPs	30 nm	5000 mg/kg	21 d	NRF2, MFN2, FIS1, DRP1 (RT-PCR)
212	Saputra F	2025	Indonesia	Zebrafish	60/60	PSNPs	25 nm	0.01, 0.1, 1, 10 μg/mL	48, 96 h	NRF2 (RT-PCR)
213	Fan Z	2024	China	Normal human hepatocyte cell line (L-02)	3/3	PSNPs	50 nm	6.25, 12.5, 25 μg/mL	24 h	p-AMPK/AMPK (WB)
214	Mohamed HRH	2025	Egypt	Human lymphoma cell line (U937)	3/3	Er_2O_3NPs	60.67 ± 4.43 nm	3.20 μg/mL	48 h	ND3 (RT-PCR)
215	Mohamed HRH	2025	Egypt	Human hepatocellular carcinoma cell line (HepG2)	3/3	Y_2O_3NPs	50 nm	13.15 μg/mL	72 h	ND3 (RT-PCR)
216	Wang L	2025	China	Human neuroblastoma cell line (SH-SY5Y)	3/3	ZnONPs	50 nm	7 μg/mL	24 h	PGC-1α, DRP1, FIS1, MFF, MFN1, MFN2 (WB)
217	Khedr MA	2025	Egypt	Male SD rats	6/6	TiO_2NPs	50 nm	150 mg/kg	28 d	NRF2 (RT-PCR)
218	Beghin M	2024	Belgium	Rainbow trout gill epithelial cell line (RTgill-W1)	9/9	TiO_2NPs; ZnONPs	22.2 ± 3 nm; 27.3 ± 4.2 nm	30 μg/mL; 0.3, 3 μg/mL	24 h	NRF2 (RT-PCR)
219	Liang B	2025	China	Male C57BL/6 mice; human colorectal adenocarcinoma cell line (Caco-2)	5/5	PSNPs	50 nm	2.5, 25, 250 mg/kg; 10, 100, 1000 μg/mL	28 d; 24 h	NRF2 (RT-PCR; WB)
220	Han D	2025	China	Male C57BL/6 mice; mouse	3/3	SiNPs	50	10 mg/kg	22 d	mtDNA content (D-loop

				cardiomyocyte cell line (HL-1)						dPCR)
221	Xia Y	2025	China	Female SD rats	6/6	TiO_2NPs	37.73 ± 5.05 nm	100, 200, 400 mg/kg	28 d	AMPK (RT-PCR)
222	Das BC	2025	India	Pearl spot (Etroplus suratensis)	36/36	PSNPs	100 nm	0.2, 2, 4 μg/mL	14 d	NRF2 (RT-PCR)
223	Yu N	2025	China	Male SD rats	6/6	TiO_2NPs	25 nm	50 mg/kg	90 d	NRF2 (RT-PCR)
224	Zheng PC	2025	China	Female C57BL/6J mice	5/5	PMMANPs	100 nm	0.2 μg/mL	60 d	NRF2 (WB)
225	Mohamed HRH	2025	Egypt	Human tongue cancer cell line (HNO-97)	3/3	$CaTiO_3$NPs	3.62 nm	262.6 μg/mL	72 h	ND3 (RT-PCR)
226	Sun D	2025	China	Planarians (Dugesia constrictiva)	3/3	CdTeQDs	3.5 nm	10, 20 μg/mL	10 d	DRP1, MFN2, OPA1, FIS1 (WB)
227	Wang C	2025	China	Mouse mammary epithelial cell line (HC11)	3/3	PSNPs	50 nm	50 μg/mL	24 h	DRP1, MFN2 (WB)
228	Poinsignon L	2025	France	Primary human villous cytotrophoblasts	7/7	PSNPs	20, 100 nm	0.01, 1, 10, 100 μg/mL	24 h	DRP1, MFN1, MFN12, OPA1, FIS1 (WB), NRF2 (IF)
229	Yu J	2025	Korea	Human intestinal epithelial cell line (Caco-2)	3/3	PSNPs	100 nm	100 μg/mL	24 h	p-AMPK/AMPK (WB)
230	Wang L	2021	China	Mouse pre-osteoblast cell line (MC3T3-E1)	3/3	TiO_2NPs	100 nm	Cells seeded on discs	1, 2, 3, 7 d	MFN1, MFN2, OPA1, DRP1 (RT-PCR)

NPs, nanoparticles; PSNPs, polystyrene NPs; SiNPs, silica NPs; ZnONPs, zinc oxide NPs; AgNPs, silver NPs; CdSQDs, cadmium sulphide quantum dots; TiO_2NPs, titanium dioxide NPs; FeNPs, iron NPs; $Ca(OH)_2$NPs, calcium hydroxide NPs; PdNPs, palladium NPs; Co_3O_4NPs, cobalt oxide NPs; SeNPs, selenium NPs; MnO_2NPs, manganese dioxide NPs; Y_2O_3NPs, yttrium oxide NPs; CBNPs, carbon black NPs; CuONPs, copper oxide NPs; AuNPs, gold NPs; Al_2O_3NPs, aluminum

oxide NPs; CdTeQDs, cadmium telluride quantum dots; HANPs, hydroxyapatite NPs; CeO_2NPs, ceria NPs; TPP, triphenylphosphonium; PtNPs, platinum NPs; NiNPs, nickel NPs; PCLNPs, polycaprolactone NPs; Fe_3O_4NPs, magnetite iron NPs; γ-Fe_2O_3NPs, maghemite iron NPs; POCA, poly(octylcyanoacrylate); CoNPs, cobalt NPs; ND, nanodiamonds; MPA, mercaptopropionic acid; PAANPs, co-ferrite-polyacrylic acid NPs; SWCNTs, single-walled carbon nanotubes; GO, graphene oxides; PEI, polyethylenimine; MWCNTs, multi-walled carbon nanotubes; SNPs, soot NPs; NW, nanowires; Ag_2SeQDs, silver selenide quantum dots; PMMANPS, polymethyl methacrylate NPs; nPM, nano-scale particulate matter; SD, Sprague-Dawley; ICR, Institute of Cancer Research; H, hour; d, day; w, week; m, month; ND1, NADH dehydrogenase subunit 1; ND2, NADH dehydrogenase subunit 2; ND3, NADH dehydrogenase subunit 3; ND4, NADH dehydrogenase subunit 4; ND6, NADH dehydrogenase subunit 6; COX1, cytochrome c oxidase subunit 1; COX2, cytochrome c oxidase subunit 2; COX3, cytochrome c oxidase subunit 3; ATPase 6, ATP synthase F0 subunit 6; ATPase 8, ATP synthase F0 subunit 8; CYTB, cytochrome b; PGC-1α, peroxisome proliferator-activated receptor-γ coactivator 1alpha; NRF1, nuclear respiratory factor-1; NRF2, nuclear respiratory factor-2; TFAM, mitochondrial transcription factor A; DRP1, dynamin-related protein 1; FIS1, fission protein 1; MFF, mitochondrial fission factor; MFN1, mitochondrial fusion protein 1; MFN2, mitochondrial fusion protein 2; OPA1, optic atrophy protein 1; AMPK, AMP-activated protein kinase; SIRT1, sirtuin 1; dPCR, DNA polymerase chain reaction; RT-PCR, reverse transcription polymerase chain reaction; WB, western blotting; IHC, immunohistochemistry; ICC, immunocytochemistry; IF, immunofluorescence; ELISA, enzyme-linked immunosorbent assay.
