# Supplementary material for: Toxicity Evaluation of Nano-Sized Particles by Analysis of mtDNA Content and Expression Levels of Genes Required for mtDNA Maintenance: A Meta-Analysis of Pre-Clinical Studies
Source: Antioxidants (Basel). 2026 Jul 4;15(7):848. doi: 10.3390/antiox15070848 (PMC13405982; doi:10.3390/antiox15070848)
Supplement: Supplementary file 1 [file antioxidants-15-00848-s001.zip › Table S3.pdf]

Table S3 Quality assessments for in vitro studies

No.	Author	Toxrtool checklist of study quality																			
		(1)	(2)	(3)	(4)	(5)	(6)	(7)	(8)	(9)	(10)	(11)	(12)	(13)	(14)	(15)	(16)	(17)	(18)	Total	Reliability of evidence
1	Sun Z	1	1	1	1	1	1	1	1	1	1	1	0	1	1	1	1	1	1	17	1
2	Qi Y	1	0	1	1	1	1	1	1	1	1	1	0	1	1	1	1	1	1	16	1
3	Li Y	1	0	1	1	1	1	1	1	1	1	1	0	1	1	1	1	1	1	16	1
4	Guo C	1	1	1	1	1	1	1	1	1	1	1	0	1	1	1	1	1	1	17	1
5	Yoisungnern T	1	0	1	1	1	1	1	1	1	1	1	0	1	1	1	1	1	1	16	1
6	Paesano L	1	1	1	1	1	1	1	1	1	1	1	0	1	1	1	1	1	1	17	1
8	Rivas-García L	1	0	1	1	1	1	1	1	1	1	1	0	1	1	1	1	1	1	16	1
10	Gurunathan S	1	0	1	1	1	1	1	1	1	1	1	0	1	1	1	1	1	1	16	1
11	Li L	1	0	1	1	1	1	1	1	1	1	1	0	1	1	1	1	1	1	16	1
12	Xu C	1	0	1	1	1	1	1	1	1	1	1	0	1	1	1	1	1	1	16	1
13	Anand AS	1	1	1	1	1	1	1	1	1	1	1	0	1	1	1	1	1	1	17	1
14	Son MJ	1	1	1	1	1	1	1	1	1	1	1	0	1	1	1	1	1	1	17	1
15	Gurunathan S	1	1	1	1	1	1	1	1	1	1	1	0	1	1	1	1	1	1	17	1
17	Xu C	1	0	1	0	1	1	1	1	1	1	1	0	1	1	1	1	1	1	15	1
19	Liu N	1	0	1	1	1	1	1	1	1	1	1	0	1	1	1	1	1	1	16	1
21	Shen Y	1	0	1	1	1	1	1	1	1	1	1	0	1	1	1	1	1	1	16	1
23	Wang G	1	0	1	0	1	1	1	1	1	1	1	0	1	1	1	1	1	1	15	1
24	Li J	1	0	1	1	1	1	1	1	1	1	1	0	1	1	1	1	1	1	16	1
27	Xu Z	1	0	1	1	1	1	1	1	1	1	1	0	1	1	1	1	1	1	16	1
29	Chang X	1	0	1	1	1	1	1	1	1	1	1	0	1	1	1	1	1	1	16	1

31	Wang L	1	1	1	1	1	1	1	1	1	1	1	0	1	1	1	1	1	1	17	1
32	Chen Y	1	0	1	1	1	1	1	1	1	1	1	0	1	1	1	1	1	1	16	1
33	Chang X	1	0	1	1	1	1	1	1	1	1	1	0	1	1	1	1	1	1	16	1
34	Wang L	1	1	1	1	1	1	1	1	1	1	1	0	1	1	1	1	1	1	17	1
36	Natarajan V	1	0	1	1	1	1	1	1	1	1	1	0	1	1	1	1	1	1	16	1
38	He Y	1	1	1	1	1	1	1	1	1	1	1	0	1	1	1	1	1	1	17	1
40	Gutiérrez-Carcedo P	1	1	1	1	1	1	1	1	1	1	1	0	1	1	1	1	1	1	17	1
43	Gurunathan S	1	0	1	1	1	1	1	1	1	1	1	0	1	1	1	1	1	1	16	1
44	Skóra B	1	0	1	1	1	1	1	1	1	1	1	0	1	1	1	1	1	1	16	1
45	Zhao X	1	0	1	1	1	1	1	1	1	1	1	0	1	1	1	1	1	1	16	1
46	Wei S	1	1	1	1	1	1	1	1	1	1	1	0	1	1	1	1	1	1	17	1
47	Lin C	1	1	1	1	1	1	1	1	1	1	1	0	1	1	1	1	1	1	17	1
48	Nguyen KC	1	0	1	1	1	1	1	1	1	1	1	0	1	1	1	1	1	1	16	1
49	Nguyen KC	1	0	1	1	1	1	1	1	1	1	1	0	1	1	1	1	1	1	16	1
50	Dey S	1	0	1	1	1	1	1	1	1	1	1	0	1	1	1	1	1	1	16	1
51	Zhao X	1	0	1	1	1	1	1	1	1	1	1	0	1	1	1	1	1	1	16	1
52	Li J	1	0	1	1	1	1	1	1	1	1	1	0	1	1	1	1	1	1	16	1
53	Zheng H	1	1	1	1	1	1	1	1	1	1	1	0	1	1	1	1	1	1	17	1
54	Bittner A	1	0	1	1	1	1	1	1	1	1	1	0	1	1	1	1	1	1	16	1
55	Wilson CL	1	0	1	1	1	1	1	1	1	1	1	0	1	1	1	1	1	1	16	1
56	Li Y	1	0	1	1	1	1	1	1	1	1	1	0	1	1	1	1	1	1	16	1
57	Fan Y	1	0	1	1	1	1	1	1	1	1	1	0	1	1	1	1	1	1	16	1
60	Fu Y	1	0	1	1	1	1	1	1	1	1	1	0	1	1	1	1	1	1	16	1
61	Liu Z	1	0	1	1	1	1	1	1	1	1	1	0	1	1	1	1	1	1	16	1
62	Tian T	1	0	1	1	1	1	1	1	1	1	1	0	1	1	1	1	1	1	16	1

63	Ko WC	1	0	1	1	1	1	1	1	1	1	1	0	1	1	1	1	1	1	16	1
64	Zhang X	1	1	1	1	1	1	1	1	1	1	1	0	1	1	1	1	1	1	17	1
65	Liu N	1	0	1	1	1	1	1	1	1	1	1	0	1	1	1	1	1	1	16	1
66	Vineetha VP	1	1	1	1	1	1	1	1	1	1	1	0	1	1	1	1	1	1	17	1
67	Zhai S	1	0	1	1	1	1	1	1	1	1	1	0	1	1	1	1	1	1	16	1
68	Yin Y	1	0	1	1	1	1	1	1	1	1	1	0	1	1	1	1	1	1	16	1
69	Li N	1	0	1	1	1	1	1	1	1	1	1	0	1	1	1	1	1	1	16	1
71	Sun X	1	0	1	1	1	1	1	1	1	1	1	0	1	1	1	1	1	1	16	1
73	Wang M	1	1	1	1	1	1	1	1	1	1	1	0	1	1	1	1	1	1	17	1
74	Wen Y	1	1	1	1	1	1	1	1	1	1	1	0	1	1	1	1	1	1	17	1
76	Chen T	1	0	1	1	1	1	1	1	1	1	1	0	1	1	1	1	1	1	16	1
77	Liu W	1	0	1	1	1	1	1	1	1	1	1	0	1	1	1	1	1	1	16	1
78	Shi Z	1	1	1	1	1	1	1	1	1	1	1	0	1	1	1	1	1	1	17	1
79	Zhang L	1	1	1	1	1	1	1	1	1	1	1	0	1	1	1	1	1	1	17	1
80	Guo C	1	0	1	1	1	1	1	1	1	1	1	0	1	1	1	1	1	1	16	1
83	Kang SJ	1	0	1	1	1	1	1	1	1	1	1	0	1	1	1	1	1	1	16	1
84	Zhang H	1	0	1	1	1	1	1	1	1	1	1	0	1	1	1	1	1	1	16	1
85	Fu X	1	0	1	1	1	1	1	1	1	1	1	0	1	1	1	1	1	1	16	1
86	Guo M	1	0	1	1	1	1	1	1	1	1	1	0	1	1	1	1	1	1	16	1
89	Ma Y	1	0	1	1	1	1	1	1	1	1	1	0	1	1	1	1	1	1	16	1
90	Cui G	1	0	1	1	1	1	1	1	1	1	1	0	1	1	1	1	1	1	16	1
91	Huang J	1	0	1	1	1	1	1	1	1	1	1	0	1	1	1	1	1	1	16	1
94	Wu Z	1	0	1	1	1	1	1	1	1	1	1	0	1	1	1	1	1	1	16	1
95	Wang Y	1	0	1	1	1	1	1	1	1	1	1	0	1	1	1	1	1	1	16	1
96	Yang J	1	0	1	1	1	1	1	1	1	1	1	0	1	1	1	1	1	1	16	1

99	Hou J	1	1	1	1	1	1	1	1	1	1	1	0	1	1	1	1	1	1	17	1
103	Li X	1	0	1	1	1	1	1	1	1	1	1	0	1	1	1	1	1	1	16	1
104	Li T	1	0	1	1	1	1	1	1	1	1	1	0	1	1	1	1	1	1	16	1
105	Zhou D	1	0	0	1	1	1	1	1	1	1	1	0	1	1	1	1	1	1	15	1
106	Zou L	1	0	1	1	1	1	1	1	1	1	1	0	1	1	1	1	1	1	16	1
107	Korakaki E	1	0	1	1	1	1	1	1	1	1	1	0	1	1	1	1	1	1	16	1
111	Magaye R	1	0	1	1	1	1	1	1	1	1	1	0	1	1	1	1	1	1	16	1
114	Zhou F	1	0	1	1	1	1	1	1	1	1	1	0	1	1	1	1	1	1	16	1
115	Liao F	1	0	1	1	1	1	1	1	1	1	1	0	1	1	1	1	1	1	16	1
116	Mytych J	1	1	1	1	1	1	1	1	1	1	1	0	1	1	1	1	1	1	17	1
118	González-Fernández C	1	0	1	1	1	1	1	1	1	1	1	0	1	1	1	1	1	1	16	1
121	Skočaj M	1	0	1	1	1	1	1	1	1	1	1	0	1	1	1	1	1	1	16	1
123	Santacruz-Márquez R	1	0	1	1	1	1	1	1	1	1	1	0	1	1	1	1	1	1	16	1
124	Berg JM	1	0	1	1	1	1	1	1	1	1	1	0	1	1	1	1	1	1	16	1
125	Ferraro SA	1	0	1	1	1	1	1	1	1	1	1	0	1	1	1	1	1	1	16	1
128	Guo C	1	0	1	1	1	1	1	1	1	1	1	0	1	1	1	1	1	1	16	1
129	Li Y	1	0	1	1	1	1	1	1	1	1	1	0	1	1	1	1	1	1	16	1
134	Wang H	1	0	1	1	1	1	1	1	1	1	1	0	1	1	1	1	1	1	16	1
135	Eom HJ	1	0	1	1	1	1	1	1	1	1	1	0	1	1	1	1	1	1	16	1
138	Eom HJ	1	0	1	1	1	1	1	1	1	1	1	0	1	1	1	1	1	1	16	1
139	Liang Y	1	0	1	1	1	1	1	1	1	1	1	0	1	1	1	1	1	1	16	1
140	Dhupal M	1	1	1	1	1	1	1	1	1	1	1	0	1	1	1	1	1	1	17	1
143	Eom HJ	1	0	1	1	1	1	1	1	1	1	1	0	1	1	1	1	1	1	16	1
149	Voicu SN	1	0	1	1	1	1	1	1	1	1	1	0	1	1	1	1	1	1	16	1
154	Yin X	1	0	1	1	1	1	1	1	1	1	1	0	1	1	1	1	1	1	16	1

155	Li X	1	1	1	1	1	1	1	1	1	1	1	0	1	1	1	1	1	1	17	1
157	Khan AA	1	0	1	1	1	1	1	1	1	1	1	0	1	1	1	1	1	1	16	1
158	Sapienza S	1	0	1	1	1	1	1	1	1	1	1	0	1	1	1	1	1	1	16	1
159	Li Y	1	0	1	1	1	1	1	1	1	1	1	0	1	1	1	1	1	1	16	1
160	Li X	1	0	1	1	1	1	1	1	1	1	1	0	1	1	1	1	1	1	16	1
161	Liang Y	1	0	1	1	1	1	1	1	1	1	1	0	1	1	1	1	1	1	16	1
162	Bai H	1	0	1	1	1	1	1	1	1	1	1	0	1	1	1	1	1	1	16	1
163	Shiwakoti S	1	0	1	1	1	1	1	1	1	1	1	0	1	1	1	1	1	1	16	1
164	Shen J	1	0	1	1	1	1	1	1	1	1	1	0	1	1	1	1	1	1	16	1
166	Chen W	1	0	1	1	1	1	1	1	1	1	1	0	1	1	1	1	1	1	16	1
167	Huang Y	1	0	1	1	1	1	1	1	1	1	1	0	1	1	1	1	1	1	16	1
170	Brown DM	1	0	1	1	1	1	1	1	1	1	1	0	1	1	1	1	1	1	16	1
171	Pelka J	1	1	1	1	1	1	1	1	1	1	1	0	1	1	1	1	1	1	17	1
172	Stanca L	1	0	1	1	1	1	1	1	1	1	1	0	1	1	1	1	1	1	16	1
173	Guo M	1	0	1	1	1	1	1	1	1	1	1	0	1	1	1	1	1	1	16	1
174	Sun R	1	0	1	1	1	1	1	1	1	1	1	0	1	1	1	1	1	1	16	1
175	Wang W	1	0	1	1	1	1	1	1	1	1	1	0	1	1	1	1	1	1	16	1
176	Wu Y	1	0	1	1	1	1	1	1	1	1	1	0	1	1	1	1	1	1	16	1
179	Liu L	1	1	1	1	1	1	1	1	1	1	1	0	1	1	1	1	1	1	17	1
180	Feng L	1	0	1	1	1	1	1	1	1	1	1	0	1	1	1	1	1	1	16	1
181	Duan WX	1	1	1	1	1	1	1	1	1	1	1	0	1	1	1	1	1	1	17	1
182	Zhao X	1	0	1	1	1	1	1	1	1	1	1	0	1	1	1	1	1	1	16	1
185	da Silva Brito WA	1	0	1	1	1	1	1	1	1	1	1	0	1	1	1	1	1	1	16	1
186	Sarikhani M	1	1	1	1	1	1	1	1	1	1	1	0	1	1	1	1	1	1	17	1
188	Li L	1	1	1	1	1	1	1	1	1	1	1	0	1	1	1	1	1	1	17	1

189	Zhang X	1	1	1	1	1	1	1	1	1	1	1	0	1	1	1	1	1	1	17	1
190	Liang C	1	0	1	1	1	1	1	1	1	1	1	0	1	1	1	1	1	1	16	1
192	Gurunathan S	1	0	1	1	1	1	1	1	1	1	1	0	1	1	1	1	1	1	16	1
194	Liu G	1	0	1	1	1	1	1	1	1	1	1	0	1	1	1	1	1	1	16	1
195	Zheng J	1	0	1	1	1	1	1	1	1	1	1	0	1	1	1	1	1	1	16	1
199	Mohamed HRH	1	0	1	1	1	1	1	1	1	1	1	0	1	1	1	1	1	1	16	1
200	Mohamed HRH	1	1	1	1	1	1	1	1	1	1	1	0	1	1	1	1	1	1	17	1
201	Yao Y	1	0	1	1	1	1	1	1	1	1	1	0	1	1	1	1	1	1	16	1
202	Zhao M	1	0	1	1	1	1	1	1	1	1	1	0	1	1	1	1	1	1	16	1
203	Li S	1	0	1	1	1	1	1	1	1	1	1	0	1	1	1	1	1	1	16	1
205	Huang F	1	0	1	1	1	1	1	1	1	1	1	0	1	1	1	1	1	1	16	1
206	Li J	1	0	1	1	1	1	1	1	1	1	1	0	1	1	1	1	1	1	16	1
207	Han M	1	0	1	1	1	1	1	1	1	1	1	0	1	1	1	1	1	1	16	1
210	Wang K	1	0	1	1	1	1	1	1	1	1	1	0	1	1	1	1	1	1	16	1
213	Fan Z	1	0	1	1	1	1	1	1	1	1	1	0	1	1	1	1	1	1	16	1
214	Mohamed HRH	1	1	1	1	1	1	1	1	1	1	1	0	1	1	1	1	1	1	17	1
215	Mohamed HRH	1	1	1	1	1	1	1	1	1	1	1	0	1	1	1	1	1	1	17	1
216	Wang L	1	0	1	1	1	1	1	1	1	1	1	0	1	1	1	1	1	1	16	1
218	Beghin M	1	1	1	1	1	1	1	1	1	1	1	0	1	1	1	1	1	1	17	1
219	Liang B	1	0	1	1	1	1	1	1	1	1	1	0	1	1	1	1	1	1	16	1
220	Han D	1	0	0	1	1	1	1	1	1	1	1	0	1	1	1	1	1	1	15	1
225	Mohamed HRH	1	1	1	1	1	1	1	1	1	1	1	0	1	1	1	1	1	1	17	1
227	Wang C	1	0	1	1	1	1	1	1	1	1	1	0	1	1	1	1	1	1	16	1
228	Poinsignon L	1	0	1	1	1	1	1	1	1	1	1	0	1	1	1	1	1	1	16	1
229	Yu J	1	0	1	1	1	1	1	1	1	1	1	0	1	1	1	1	1	1	16	1

230	Wang L	1	1	1	1	1	1	1	1	0	1	1	0	1	1	1	1	1	1	16	1

(1)Test substance identification; (2) substance purity statement; (3) the source/origin information of the substance; (4) information on physicochemical properties of the test item given; (5) cell culture description; (6) the source/origin of cell culture; (7) necessary information on cell culture properties, conditions of cultivation and maintenance; (8) the method of particle administration; (9) doses or concentration statement; (10) frequency and duration of exposure as well as time-points of observations statement; (11) have negative controls; (12) have positive controls; (13) the number of replicates; (14) are the study endpoint(s) and their method(s) of determination clearly described?; (15) is the description of the study results for all endpoints investigated transparent and complete?; (16) are the statistical methods for data analysis given and applied in a transparent manner?; (17) is the study design chosen appropriate for obtaining the substance-specific data aimed at?; (18) are the quantitative study results reliable? The values of 0 (not satisfied the criteria) or 1 (satisfied the criteria) point allocated for each item. Reliable level of evidence: 1, score 15-18, reliable without restrictions; 2, score 11-14, reliable with restrictions; 3, score < 11, not reliable.
