# Supplementary material for: Toxicity Evaluation of Nano-Sized Particles by Analysis of mtDNA Content and Expression Levels of Genes Required for mtDNA Maintenance: A Meta-Analysis of Pre-Clinical Studies
Source: Antioxidants (Basel). 2026 Jul 4;15(7):848. doi: 10.3390/antiox15070848 (PMC13405982; doi:10.3390/antiox15070848)
Supplement: Supplementary file 1 [file antioxidants-15-00848-s001.zip › Table S4.pdf]

Table S4 Quality assessments for in vivo studies

No.	Author	Toxrtool checklist of study quality																						
		(1)	(2)	(3)	(4)	(5)	(6)	(7)	(8)	(9)	(10)	(11)	(12)	(13)	(14)	(15)	(16)	(17)	(18)	(19)	(20)	(21)	Total	Reliability of evidence
7	Mohamed HRH	1	1	1	1	1	1	1	1	1	1	1	1	1	1	1	1	1	1	1	1	1	21	12
9	Mohamed HRH	1	0	1	1	1	1	1	1	1	1	1	1	1	1	1	1	1	1	1	1	1	20	1
11	Li L	1	0	1	1	1	1	1	1	0	1	1	1	1	1	1	1	1	1	1	1	1	19	1
16	Abd El-Maksoud EM	1	0	1	1	1	1	1	1	1	1	1	1	1	1	1	1	1	1	1	1	1	20	1
18	Zhao T	1	1	1	1	1	0	1	1	1	1	1	1	1	1	1	1	1	1	1	1	1	20	1
20	Baghaee P	1	1	1	1	1	1	1	1	1	1	1	1	1	1	1	1	1	1	1	1	1	21	1
22	Zhou H	1	0	1	1	1	1	1	1	1	1	1	1	1	1	1	1	1	1	1	1	1	20	1
25	Chang X	1	0	1	1	1	1	1	1	1	1	1	1	1	1	1	1	1	1	1	1	1	20	1
26	Yousef MI	1	0	1	1	1	1	1	1	1	1	1	1	1	1	1	1	1	1	1	1	1	20	1
27	Xu Z	1	0	1	1	1	1	1	1	1	1	1	1	1	1	1	1	1	1	1	1	1	20	1
28	Yousef MI	1	0	1	1	1	1	1	1	1	1	1	1	1	1	1	1	1	1	1	1	1	20	1
30	Nguyen KC	1	0	1	1	1	1	1	1	1	1	1	1	1	1	1	1	1	1	1	1	1	20	1
35	Arslan NP	1	1	1	1	1	1	1	1	1	1	1	1	1	1	1	1	1	1	1	1	1	21	1
37	Ma W	1	0	1	1	1	1	1	1	1	1	1	1	1	1	1	1	1	1	1	1	1	20	1
39	Yousef MI	1	0	1	1	1	1	1	1	1	1	1	1	1	1	1	1	1	1	1	1	1	20	1
41	Hong F	1	0	1	1	1	1	1	1	1	1	1	1	1	1	1	1	1	1	1	1	1	20	1
42	Eldeeb GM	1	0	1	1	1	1	1	1	1	1	1	1	1	1	1	1	1	1	1	1	1	20	1

52	Li J	1	0	1	1	1	1	1	1	1	1	1	1	1	1	1	1	1	1	1	1	1	20	1
57	Fan Y	1	0	1	1	1	1	1	1	1	1	1	1	1	1	1	1	1	1	1	1	1	20	1
58	Ma W	1	0	1	1	1	1	1	1	1	1	1	1	1	1	1	1	1	1	1	1	1	20	1
59	Kong L	1	1	1	1	1	1	1	1	1	1	1	1	1	1	1	1	1	1	1	1	1	21	1
64	Zhang X	1	1	1	1	1	1	1	1	1	1	1	1	1	1	1	1	1	1	1	1	1	21	1
66	Vineetha VP	1	1	0	1	1	0	1	1	1	1	1	1	1	1	1	1	1	1	1	1	1	19	1
69	Li N	1	0	1	1	1	1	1	1	0	1	1	1	1	1	1	1	1	1	1	1	1	20	1
70	Mahmoud AM	1	0	1	1	1	1	1	1	1	1	1	1	1	1	1	1	1	1	1	1	1	20	1
72	Hong F	1	1	1	1	1	1	1	1	1	1	1	1	1	1	1	1	1	1	1	1	1	21	1
74	Wen Y	1	1	1	1	1	1	1	1	1	1	1	1	1	1	1	1	1	1	1	1	1	21	1
75	Maher AM	1	0	1	1	1	1	1	1	1	1	1	1	1	1	1	1	1	1	1	1	1	20	1
79	Zhang L	1	0	1	1	1	1	1	1	0	1	1	1	1	1	1	1	1	1	1	1	1	19	1
81	Elblehi SS	1	0	1	1	1	1	1	1	1	1	1	1	1	1	1	1	1	1	1	1	1	20	1
82	Abdou KH	1	0	1	1	1	1	1	1	1	1	1	1	1	1	1	1	1	1	1	1	1	20	1
85	Fu X	1	0	1	1	1	1	1	1	1	1	1	1	1	1	1	1	1	1	1	1	1	20	1
87	Abdelrahman SA	1	1	1	1	1	1	1	1	1	1	1	1	1	1	1	1	1	1	1	1	1	21	1
88	Teng J	1	0	0	1	1	0	1	1	1	1	1	1	1	1	1	1	1	1	1	1	1	18	1
89	Ma Y	1	0	1	1	1	1	1	1	1	1	1	1	1	1	1	1	1	1	1	1	1	20	1
92	Zheng F	1	0	1	1	1	1	1	1	1	1	1	1	1	1	1	1	1	1	1	1	1	20	1
93	Hashim AR	1	0	1	1	1	1	1	1	1	1	1	1	1	1	1	1	1	1	1	1	1	20	1
97	Salama B	1	0	1	1	1	1	1	1	1	1	1	1	1	1	1	1	1	1	1	1	1	20	1
98	Fonseca E	1	0	1	1	1	0	1	1	1	1	1	1	1	1	1	1	1	1	1	1	1	19	1
100	Kandeil MA	1	0	1	1	1	1	1	1	1	1	1	1	1	1	1	1	1	1	1	1	1	20	1

101	Alshammari GM	1	0	1	1	1	1	1	1	1	1	1	1	1	1	1	1	1	1	1	1	1	20	1
102	Nemmar A	1	0	1	1	1	1	1	1	1	1	1	1	1	1	1	1	1	1	1	1	1	20	1
108	Li X	1	0	1	1	1	0	0	0	0	1	1	1	1	1	1	1	1	1	1	1	1	16	2
109	Afshari-Kaveh M	1	0	1	1	1	1	1	1	1	1	1	1	1	1	1	1	1	1	1	1	1	20	1
110	Rajkumar KS	1	0	1	1	1	0	1	1	1	1	1	1	1	1	1	1	1	1	1	1	1	19	1
111	Magaye R	1	0	1	1	1	1	1	1	1	1	1	1	1	1	1	1	1	1	1	1	1	20	1
112	Noshy PA	1	0	1	1	1	1	1	1	1	1	1	1	1	1	1	1	1	1	1	1	1	20	1
113	Zhu B	1	1	1	1	1	0	1	1	1	1	1	1	1	1	1	1	1	1	1	1	1	20	1
117	Yasin NAE	1	0	1	1	1	1	1	1	1	1	1	1	1	1	1	1	1	1	1	1	1	20	1
119	Tian J	1	0	1	1	1	0	1	1	1	1	1	1	1	1	1	1	1	1	1	1	1	19	1
120	Krishnasamy Sekar R	1	1	1	1	1	0	1	1	1	1	1	1	1	1	1	1	1	1	1	1	1	20	1
122	Niu L	1	0	1	1	1	1	1	1	1	1	1	1	1	1	1	1	1	1	1	1	1	20	1
126	Sun Q	1	0	1	1	1	1	1	1	1	1	1	1	1	1	1	1	1	1	1	1	1	20	1
127	Ucar A	1	0	1	1	1	0	1	1	1	1	1	1	1	1	1	1	1	1	1	1	1	19	1
130	Kaur K	1	0	1	1	1	0	1	1	1	1	1	1	1	1	1	1	1	1	1	1	1	19	1
131	Qiao L	1	0	1	1	1	1	1	1	1	1	1	1	1	1	1	1	1	1	1	1	1	20	1
132	Hassanen EI	1	0	1	1	1	1	1	1	1	1	1	1	1	1	1	1	1	1	1	1	1	20	1
133	Liu W	1	0	1	1	1	1	1	1	0	1	1	1	1	1	1	1	1	1	1	1	1	19	1
136	Liu X	1	0	1	1	1	1	1	1	1	1	1	1	1	1	1	1	1	1	1	1	1	20	1
137	Wang J	1	1	1	1	1	1	1	1	1	1	1	1	1	1	1	1	1	1	1	1	1	21	1
139	Liang Y	1	0	1	1	1	1	1	1	1	1	1	1	1	1	1	1	1	1	1	1	1	20	1

141	Brown DM	1	0	1	1	1	1	1	1	0	1	1	1	1	1	1	1	1	1	1	1	1	19	1
142	da Rocha AM	1	0	1	1	1	0	1	1	1	1	1	1	1	1	1	1	1	1	1	1	1	19	1
144	Ze Y	1	1	1	1	1	1	1	1	1	1	1	1	1	1	1	1	1	1	1	1	1	21	1
145	Kluknavsky M	1	0	1	1	1	1	1	1	1	1	1	1	1	1	1	1	1	1	1	1	1	20	1
146	Eid A	1	0	1	1	1	1	1	1	1	1	1	1	1	1	1	1	1	1	1	1	1	20	1
147	Alghriany AAI	1	0	1	1	1	1	0	1	1	1	1	1	1	1	1	1	1	1	1	1	1	19	1
148	Wei S	1	1	1	1	1	0	1	1	1	1	1	1	1	1	1	1	1	1	1	1	1	20	1
150	Micurova A	1	0	1	1	1	1	1	1	1	1	1	1	1	1	1	1	1	1	1	1	1	20	1
151	Abd-Eltawab Tammam A	1	0	1	1	1	1	1	1	1	1	1	1	1	1	1	1	1	1	1	1	1	20	1
152	Dou X	1	0	1	1	1	1	1	1	1	1	1	1	1	1	1	1	1	1	1	1	1	20	1
153	Kuang H	1	0	1	1	1	1	1	1	1	1	1	1	1	1	1	1	1	1	1	1	1	20	1
156	Yang S	1	0	1	1	1	1	1	1	1	1	1	1	1	1	1	1	1	1	1	1	1	20	1
161	Liang Y	1	0	1	1	1	1	1	1	1	1	1	1	1	1	1	1	1	1	1	1	1	20	1
162	Bai H	1	0	1	1	1	1	1	1	1	1	1	1	1	1	1	1	1	1	1	1	1	20	1
165	Tang Y	1	0	1	1	1	1	1	1	1	1	1	1	1	1	1	1	1	1	1	1	1	20	1
168	Zhang C	1	0	1	1	1	0	1	1	1	1	1	1	1	1	1	1	1	1	1	1	1	19	1
169	Fernandes AL	1	0	1	1	1	1	1	1	1	1	1	1	1	1	1	1	1	1	1	1	1	20	1
176	Wu Y	1	0	1	1	1	1	1	1	1	1	1	1	1	1	1	1	1	1	1	1	1	20	1
177	Ling X	1	0	1	1	1	0	1	1	1	1	1	1	1	1	1	1	1	1	1	1	1	19	1
178	Ijaz MU	1	0	1	1	1	0	1	1	1	1	1	1	1	1	1	1	1	1	1	1	1	19	1

179	Liu L	1	1	1	1	1	1	1	1	1	1	1	1	1	1	1	1	1	1	1	1	1	21	1
182	Zhao X	1	0	1	1	1	1	1	1	1	1	1	1	1	1	1	1	1	1	1	1	1	20	1
183	Cai P	1	0	1	1	1	1	1	1	1	1	1	1	1	1	1	1	1	1	1	1	1	20	
184	Abdel Aal SM	1	0	1	1	1	1	1	1	1	1	1	1	1	1	1	1	1	1	1	1	1	20	1
187	Hsiao TC	1	0	1	1	1	1	1	1	1	1	1	1	1	1	1	1	1	1	1	1	1	20	1
191	Liu J	1	0	1	1	1	1	1	1	1	1	1	1	1	1	1	1	1	1	1	1	1	20	1
192	Dora MF	1	0	1	1	1	1	1	1	1	1	1	1	1	1	1	1	1	1	1	1	1	20	1
195	Zheng J	1	0	1	1	1	1	1	1	1	1	1	1	1	1	1	1	1	1	1	1	1	20	1
196	Boukholda K	1	0	1	1	1	1	1	1	1	1	1	1	1	1	1	1	1	1	1	1	1	20	1
197	Chen Y	1	0	1	1	1	1	1	1	1	1	1	1	1	1	1	1	1	1	1	1	1	20	1
198	El Henafy HMA	1	0	1	1	1	1	1	1	1	1	1	1	1	1	1	1	1	1	1	1	1	20	1
202	Zhao M	1	0	1	1	1	1	1	1	1	1	1	1	1	1	1	1	1	1	1	1	1	20	1
203	Zhang Y	1	0	1	1	1	1	1	1	1	1	1	1	1	1	1	1	1	1	1	1	1	20	1
206	Li J	1	0	1	1	1	1	1	1	1	1	1	1	1	1	1	1	1	1	1	1	1	20	1
208	Gao X	1	0	1	1	1	0	1	1	1	1	1	1	1	1	1	1	1	1	1	1	1	19	1
209	Khayal EE	1	1	1	1	1	1	1	1	1	1	1	1	1	1	1	1	1	1	1	1	1	21	1
211	He X	1	0	1	1	1	1	1	1	1	1	1	1	1	1	1	1	1	1	1	1	1	20	1
212	Saputra F	1	0	1	1	1	0	1	1	1	1	1	1	1	1	1	1	1	1	1	1	1	19	1
217	Khedr MA	1	0	1	1	1	1	1	1	1	1	1	1	1	1	1	1	1	1	1	1	1	20	1
219	Liang B	1	0	1	1	1	1	1	1	1	1	1	1	1	1	1	1	1	1	1	1	1	20	1
220	Han D	1	0	0	1	1	1	1	1	1	1	1	1	1	1	1	1	1	1	1	1	1	19	1
221	Xia Y	1	0	1	1	1	1	1	1	1	1	1	1	1	1	1	1	1	1	1	1	1	20	1
222	Das BC	1	0	1	1	1	0	1	1	1	1	1	1	1	1	1	1	1	1	1	1	1	19	1

223	Yu N	1	1	1	1	1	1	1	1	1	1	1	1	1	1	1	1	1	1	1	1	1	21	1
224	Zheng PC	1	0	1	1	1	1	1	1	1	1	1	1	1	1	1	1	1	1	1	1	1	20	1
226	Sun D	1	0	1	1	1	0	1	1	1	1	1	1	1	1	1	1	1	1	1	1	1	19	1

(1)Test substance identification; (2) substance purity statement; (3) the source/origin information of the substance; (4) information on physicochemical properties of the test item given; (5) species description; (6) the sex of the test organism; (7) information on the strain of test animals plus; (8) age or body weight of the test organisms; (9) information given on the housing or feeding conditions; (10) the administration route given; (11) doses or concentration statement; (12) frequency and duration of exposure as well as time-points of observations statement; (13) have negative and positive controls; (14) the number of animals per group; (15) sufficient details of the administration scheme; (16) concentrations analytically verified; (17) Are the study endpoint(s) and their method(s) of determination clearly described?; (18) is the description of the study results for all endpoints investigated transparent and complete?; (19) are the statistical methods for data analysis given and applied in a transparent manner?; (20) is the study design chosen appropriate for obtaining the substance-specific data aimed at?; (21) are the quantitative study results reliable? The values of 0 (not satisfied the criteria) or 1 (satisfied the criteria) point allocated for each item. Reliable level of evidence: 1, score 18-21, reliable without restrictions; 2, score 13-17, reliable with restrictions; 3, score < 13, not reliable.
