# Supplementary material for: Toxicity Evaluation of Nano-Sized Particles by Analysis of mtDNA Content and Expression Levels of Genes Required for mtDNA Maintenance: A Meta-Analysis of Pre-Clinical Studies
Source: Antioxidants (Basel). 2026 Jul 4;15(7):848. doi: 10.3390/antiox15070848 (PMC13405982; doi:10.3390/antiox15070848)
Supplement: Supplementary file 1 [file antioxidants-15-00848-s001.zip › Table S5.pdf]

Table S5 Subgroup meta-analysis for in vitro studies

**Variable**			No.	SMD	95%CI	P_{ES}-value	I^2	P_H-value	Model
**mtDNA content**			69	-1.08	-1.72,-0.45	**0.001**	81.0	< 0.001	R
Country	Asian		58	-1.39	-2.29,-0.48	**0.003**	80.6	< 0.001	R
	Non-Asian		11	-0.94	-1.73,-0.15	**0.020**	83.7	< 0.001	R
Particle type	AgNPs		7	1.44	-1.82,4.69	0.387	81.9	< 0.001	R
	CBNPs		5	-9.65	-14.29,-5.01	**< 0.001**	44.3	0.127	F
	QDs		24	-0.41	-1.04,0.21	0.197	74.9	< 0.001	R
	FeNPs		2	-2.93	-3.90,-1.95	< 0.001	0.0	0.581	F
	NiNPs		5	-14.34	-18.93,-9.74	**< 0.001**	0.0	0.807	F
	PSNPs		3	-0.43	-4.52,3.66	0.837	83.2	0.003	R
	PdNPs		1	-9.26	-15.88,-2.65	0.006	-	-	R
	PtNPs		7	-4.56	-6.88,-2.23	**< 0.001**	64.1	0.010	R
	SeNPs		1	-3.40	-6.25,-0.55	0.019	-	-	R
	SiNPs		9	-0.79	-2.54,0.95	0.373	76.5	< 0.001	R
	TiO_2NPs		2	2.07	0.55,3.59	0.008	0.0	0.825	F
	ZnONPs		3	3.22	-2.84,9.29	0.298	88.1	< 0.001	R
Cell type	Species	Human	41	-1.80	-2.56,-1.05	**< 0.001**	81.1	< 0.001	R
		Murine (rat)	4	-11.28	-18.08,-4.48	**0.001**	55.0	0.083	F
		Murine (mouse)	24	0.71	-0.35,1.77	0.192	76.7	< 0.001	R
		Murine (total)	28	0.16	-0.99,1.32	0.781	79.4	< 0.001	R
	Tissue source	Placenta (HTR-8/SVneo)	6	-11.65	-15.38,-7.92	**< 0.001**	8.2	0.364	F
		Lung (A549, primary)	2	-3.08	-14.29,8.13	0.590	90.4	0.001	R
		Liver (L-02, HepG2)	12	-0.33	-0.99,0.33	0.322	75.4	< 0.001	R
		Heart (H9C2, stem cell-derived	5	2.67	-1.51,6.84	0.211	86.3	< 0.001	R

		cardiomyocytes, HL-1)							
		Testis (TM3, primary sperm)	7	2.37	-0.06,4.79	0.056	75.9	< 0.001	R
		Colorectum (HT-29, NCM460)	3	-2.98	-3.90,-2.06	**< 0.001**	0.0	0.819	F
		Vessel (HUVECs)	4	-3.34	-6.59,-0.09	**0.044**	74.0	0.009	R
		Bone-muscle (BM-MSCs)	4	-11.28	-18.08,-4.48	**0.001**	55.0	0.083	F
		Blood immune (THP-1, RAW264.7)	22	-1.33	-2.47,-0.19	**0.022**	76.3	< 0.001	R
		Neuron (SH-SY5Y)	4	-1.11	-4.72,2.51	0.549	84.9	< 0.001	R
Particle dose	≤ 50 μg/mL		59	-0.74	-1.38,-0.10	**0.023**	80.7	< 0.001	R
	> 50 μg/mL		10	-5.09	-8.10,-2.08	**0.001**	83.2	< 0.001	R
Particle duration	≤ 24 h		63	-0.79	-1.42,-0.16	**0.014**	79.6	< 0.001	R
	> 24 h		6	-5.38	-8.14,-2.62	**< 0.001**	72.2	0.003	R
Assay method (dPCR amplification of mtDNA-encoded genes)	ND1		21	-0.89	-2.23,0.46	0.197	82.0	< 0.001	R
	ND4		3	-1.10	-2.07,-0.12	**0.027**	62.9	0.068	F
	ND1-ND4		2	-2.93	-3.90,-1.95	< 0.001	0.0	0.581	F
	ND1/5		1	-7.31	-12.63,-2.00	0.007	-	-	R
	ND6		3	-3.82	-5.61,-2.03	**< 0.001**	0.0	0.937	F
	COX1		7	-0.51	-2.72,1.69	0.648	80.0	< 0.001	R
	CYTB		19	-1.21	-2.42,0.01	0.051	81.0	< 0.001	R
	ATP6		3	4.02	-3.08,11.12	0.267	88.9	< 0.001	R
	12S rRNA		8	-1.24	-2.83,0.36	0.128	72.6	0.001	R
	D-loop		2	2.28	-9.48,14.05	0.704	91.6	0.001	R
**Expression of mitochondrial biogenesis-related gene PGC-1α**			66	-3.03	-3.88,-2.18	**< 0.001**	78.3	< 0.001	R
Country	Asian		61	-3.27	-4.15,-2.40	**< 0.001**	77.3	< 0.001	R
	Non-Asian		5	-0.20	-3.63,3.23	0.910	83.9	< 0.001	R
Particle type	AgNPs		20	-4.20	-5.76,-2.64	**< 0.001**	76.5	< 0.001	R

	SiNPs		10	-1.87	-3.10,-0.64	**0.003**	60.8	0.006	R
	ZnONPs		12	-1.08	-2.23,0.07	0.064	67.9	< 0.001	R
	PdNPs		1	-14.00	-23.83,-4.17	0.005	-	-	R
	QDs		4	1.44	-2.12,5.01	0.427	81.9	0.001	R
	CBNPs		4	-4.69	-6.52,-2.86	**< 0.001**	0.0	0.880	F
	PtNPs		7	-11.71	-17.74,-5.68	**< 0.001**	76.2	< 0.001	R
	PSNPs		8	-8.91	-15.96,-1.85	**0.013**	88.0	< 0.001	R
Cell type	Species	Human	44	-3.18	-4.18,-2.18	**< 0.001**	76.5	< 0.001	R
		Murine (mouse)	18	-2.26	-4.05,-0.46	**0.014**	82.5	< 0.001	R
		Murine (rat)	4	-4.69	-6.52,-2.86	**< 0.001**	0.0	0.880	F
		Murine (total)	22	-2.75	-4.38,-1.13	**0.001**	81.8	< 0.001	R
	Tissue source	Adipose (brown preadipocytes)	2	-1.58	-2.96,-0.20	0.024	0.0	0.703	F
		Blood immune (THP-1, RAW264.7)	10	-6.46	-10.52,-2.39	**0.002**	85.7	< 0.001	R
		Bone-muscle (BM-MSCs)	4	-4.69	-6.52,-2.86	**< 0.001**	0.0	0.880	F
		Neuron (SH-SY5Y, HT22)	18	-2.65	-4.02,-1.28	**< 0.001**	75.9	< 0.001	R
		Heart (stem cell-derived cardiomyocytes)	3	-7.25	-13.50,-0.99	**0.023**	71.0	0.032	R
		Kidney (HEK293T)	3	-1.73	-3.08,-0.39	**0.012**	40.8	0.185	F
		Liver (L-02, HepG2)	15	-3.39	-5.38,-1.40	**0.001**	78.2	< 0.001	R
		Lung (A549)	1	-14.00	-23.83,-4.17	0.005	-	-	R
		Testis (TM4, GC-2 spd(ts), GC-1 spg)	6	0.39	-2.40,3.18	0.785	81.8	< 0.001	R
		Vessel (HUVECs)	4	-6.30	-11.30,-1.31	**0.013**	70.5	0.017	R
Particle dose	≤ 50 μg/mL		56	-2.85	-3.68,-2.02	**< 0.001**	76.4	< 0.001	R
	> 50 μg/mL		10	-7.17	-12.23,-2.12	**0.005**	86.4	< 0.001	R
Particle duration	≤ 24 h		60	-2.94	-3.83,-2.05	**< 0.001**	78.3	< 0.001	R
	> 24 h		6	-3.83	-7.15,-0.51	**0.024**	81.2	< 0.001	R

Assay method	mRNA (RT-PCR)		17	-4.76	-6.52,-3.00	**< 0.001**	73.1	< 0.001	R
	Protein (WB, ELISA, IF)		49	-2.52	-3.49,-1.56	**< 0.001**	79.0	< 0.001	R
**Expression of mtDNA maintenance gene MFN1**			87	-1.26	-1.87,-0.64	**< 0.001**	88.4	< 0.001	R
Country	Asian		72	-1.79	-2.46,-1.12	**< 0.001**	87.2	< 0.001	R
	Non-Asian		15	0.74	-0.21,1.69	0.127	76.3	< 0.001	R
Particle type	SiNPs		14	-2.51	-3.82,-1.20	**< 0.001**	70.9	< 0.001	R
	ZnONPs		11	-1.84	-3.39,-0.30	**0.020**	79.2	< 0.001	R
	QDs		3	-3.34	-6.92,0.25	0.068	68.4	0.042	R
	CBNPs		7	-1.87	-3.21,-0.53	**0.006**	58.5	0.025	R
	AgNPs		9	4.26	2.00,6.52	**< 0.001**	73.9	< 0.001	R
	TiO_2NPs		15	0.29	-1.39,1.97	0.738	82.2	< 0.001	R
	PSNPs		23	-1.36	-2.44,-0.28	**0.014**	94.2	< 0.001	R
	NiNPs		5	-12.43	-19.11,-5.76	**< 0.001**	63.2	0.028	R
Cell type	Species	Human	51	-0.98	-1.59,-0.37	**0.002**	75.5	< 0.001	R
		Murine (mouse)	22	-1.76	-3.51,-0.01	**0.049**	80.9	< 0.001	R
		Murine (rat)	11	1.13	-1.34,3.60	0.371	85.2	< 0.001	R
		Murine (total)	33	-0.75	-2.15,0.65	0.294	82.4	< 0.001	R
		Porcine	3	-3.71	-5.54,-1.89	**< 0.001**	97.9	< 0.001	R
	Tissue source	Blood immune (RAW264.7)	3	-3.34	-6.92,0.25	0.068	68.4	0.042	R
		Bone-muscle (BM-MSCs, MC3T3-E1)	6	-2.72	-4.88,-0.55	**0.014**	73.2	0.002	R
		Neuron (SH-SY5Y, HT22, primary cortical astrocytes, BV-2)	22	1.64	0.31,2.98	**0.016**	79.5	< 0.001	R
		Heart (HL-1, AC16)	8	-3.81	-5.87,-1.75	**< 0.001**	68.7	0.002	R
		Liver (L-02, HepG2, primary hepatocytes)	17	-1.89	-3.25,-0.54	**0.006**	78.6	< 0.001	R
		Lung (A549, 16HBE)	6	-5.29	-8.42,-2.15	**0.001**	80.2	< 0.001	R

		Ovarian (oocytes)	3	-3.71	-5.54,-1.89	**< 0.001**	97.9	< 0.001	R
		Placenta (HTR-8/SVneo, primary villous cytotrophoblasts)	7	0.37	-0.05,0.79	0.085	0.0	0.809	F
		Stomach (GES-1)	3	-0.33	-3.91,3.26	0.858	79.3	0.008	R
		Testis (TM4, GC-2 spd, GC-1)	6	-9.52	-14.31,-4.74	**< 0.001**	64.8	0.014	R
		Vessel (HUVECs, HBVPs)	6	0.73	-0.82,2.28	0.358	69.1	0.006	R
Particle dose	≤ 50 μg/mL		66	-1.24	-1.97,-0.51	**0.001**	88.7	< 0.001	R
	> 50 μg/mL		17	-1.37	-3.17,0.44	0.138	89.4	< 0.001	R
	Unknown		4	-2.26	-5.12,0.59	0.120	78.9	0.003	R
Particle duration	≤ 24 h		76	-0.81	-1.43,-0.19	**0.010**	78.9	< 0.001	R
	> 24 h		11	-3.33	-4.46,-2.20	**< 0.001**	91.3	< 0.001	R
Assay method	mRNA (RT-PCR)		23	-0.55	-1.66,0.56	0.331	92.5	< 0.001	R
	Protein (WB, IF)		64	-1.43	-2.08,-0.77	**< 0.001**	78.3	< 0.001	R
**Expression of mtDNA maintenance gene MFN2**			102	-0.96	-1.58,-0.35	**0.002**	90.0	< 0.001	R
Country	Asian		87	-1.48	-2.19,-0.76	**< 0.001**	90.8	< 0.001	R
	Non-Asian		15	1.06	0.05,2.07	**0.039**	76.4	< 0.001	R
Particle type	AgNPs		9	2.81	0.83,4.78	**0.005**	76.7	< 0.001	R
	CBNPs		9	-1.79	-2.74,-0.83	**< 0.001**	39.8	0.102	F
	QDs		3	-3.19	-4.78,-1.61	**< 0.001**	0.0	0.927	F
	NiNPs		5	-14.17	-18.78,-9.55	**< 0.001**	0.0	0.585	F
	PSNPs		27	-0.01	-0.90,0.88	0.985	93.1	< 0.001	R
	SiNPs		28	-2.29	-3.46,-1.12	**< 0.001**	78.3	< 0.001	R
	TiO_2NPs		10	2.49	0.12,4.87	**0.039**	82.4	< 0.001	R
	ZnONPs		11	-2.24	-3.98,-0.50	**0.012**	80.1	< 0.001	R
Cell type	Species	Human	49	-0.50	-1.07,0.07	0.083	74.0	< 0.001	R

		Murine (mouse)	37	-2.28	-3.29,-1.28	**< 0.001**	74.0	< 0.001	R
		Murine (rat)	13	0.66	-1.53,2.84	0.556	83.2	< 0.001	R
		Murine (total)	50	-1.58	-2.51,-0.66	**0.001**	77.1	< 0.001	R
		Porcine	3	3.78	3.12,4.45	**< 0.001**	83.7	0.002	R
	Tissue source	Bone-muscle (BM-MSCs, MC3T3-E1)	8	-2.08	-3.50,-0.66	**0.004**	63.5	0.008	R
		Breast (HC11)	1	-1.82	-3.86,0.22	0.080	-	-	R
		Neuron (SH-SY5Y, HT22, primary cortical astrocytes, BV-2, N2a, stem cell-derived)	34	-0.29	-1.51,0.93	0.641	80.7	< 0.001	R
		Heart (HL-1, AC16)	8	-2.81	-4.04,-1.59	**< 0.001**	37.7	0.128	F
		Blood immune (RAW264.7)	3	-3.19	-4.78,-1.61	**< 0.001**	0.0	0.927	F
		Liver (L-02, HepG2)	14	0.23	-0.76,1.21	0.655	67.9	< 0.001	R
		Lung (A549, 16HBE)	6	-2.59	-4.65,-0.53	**0.001**	79.5	< 0.001	R
		Ovarian (oocytes)	3	3.78	3.12,4.45	**< 0.001**	83.7	0.002	R
		Placenta (HTR-8/SVneo, primary villous cytotrophoblasts)	7	0.63	0.20,1.07	**0.004**	0.0	0.465	F
		Stomach (GES-1)	3	-0.92	-2.90,1.06	0.363	64.8	0.059	R
		Testis (TM4, GC-2 spd, GC-1)	6	-12.36	-19.63,-5.08	**0.001**	78.2	< 0.001	R
		Vessel (HUVECs, HBVPs, rBCEC4)	9	0.09	-1.38,1.56	0.907	74.9	0.010	R
Particle dose	≤ 50 μg/mL		79	-0.94	-1.64,-0.24	**0.009**	89.7	< 0.001	R
	> 50 μg/mL		19	-1.07	-2.70,0.57	0.201	90.0	< 0.001	R
	Unknown		4	-1.34	-3.35,0.68	0.194	70.8	0.016	R
Particle duration	≤ 24 h		92	-0.92	-1.47,-0.38	**0.001**	78.1	< 0.001	R
	> 24 h		10	-0.21	-1.61,1.20	0.775	94.7	< 0.001	R
Assay method	mRNA (RT-PCR)		20	1.60	0.58,2.62	**0.002**	90.0	< 0.001	R
	Protein (WB, IF)		82	-1.47	-2.03,-0.91	**< 0.001**	77.2	< 0.001	R

**Expression of mtDNA maintenance gene OPA1**			101	-1.91	-2.44,-1.39	**< 0.001**	87.9	< 0.001	R
Country	Asian		86	-2.20	-2.81,-1.58	**< 0.001**	88.8	< 0.001	R
	Non-Asian		15	-0.78	-1.57,0.006	0.052	73.5	< 0.001	R
Particle type	AgNPs		9	-0.17	-2.85,2.52	0.903	82.7	< 0.001	R
	CBNPs		7	-2.61	-4.68,-0.54	**0.013**	75.0	< 0.001	R
	QDs		3	-1.06	-3.84,1.73	0.458	77.3	0.012	R
	FeNPs		4	-6.62	-10.23,-3.00	**< 0.001**	48.2	0.122	F
	NiNPs		5	-15.39	-24.18,-6.61	**0.001**	68.3	0.013	R
	PSNPs		26	-0.92	-1.73,-0.11	**0.026**	91.9	< 0.001	R
	SiNPs		28	-2.22	-2.99,-1.46	**< 0.001**	62.0	< 0.001	R
	TiO_2NPs		9	-1.41	-2.93,0.11	0.069	75.7	< 0.001	R
	ZnONPs		10	-1.51	-3.03,0.02	0.053	79.2	< 0.001	R
Cell type	Species	Human	51	-1.10	-1.69,-0.51	**< 0.001**	74.3	< 0.001	R
		Murine (mouse)	36	-3.40	-4.43,-2.37	**< 0.001**	73.0	< 0.001	R
		Murine (rat)	11	-2.70	-3.91,-1.48	**< 0.001**	60.9	0.004	R
		Murine (total)	47	-3.15	-3.95,-2.35	**< 0.001**	70.4	< 0.001	R
		Porcine	3	2.30	0.77,3.82	**0.003**	98.1	< 0.001	R
	Tissue source	Placenta (HTR-8/SVneo, primary villous cytotrophoblasts)	7	0.62	0.19,1.05	**0.005**	0.0	0.612	F
		Blood immune (RAW264.7)	3	-1.06	-3.84,1.73	0.458	77.3	0.012	R
		Bone-muscle (BM-MSCs, MC3T3-E1)	6	-2.84	-5.94,0.86	0.143	84.7	< 0.001	R
		Neuron (SH-SY5Y, HT22, BV-2)	29	-2.51	-3.38,-1.65	**< 0.001**	67.5	< 0.001	R
		Heart (HL-1, AC16)	8	-1.52	-2.36,-0.67	**< 0.001**	24.0	0.238	F
		Liver (L-02, HepG2, primary hepatocytes)	17	-1.34	-2.90,0.22	0.092	80.6	< 0.001	R
		Lung (A549, 16HBE)	6	-2.96	-5.32,-0.60	**0.014**	82.0	< 0.001	R

		Ovarian (oocytes)	3	2.30	0.77,3.82	**0.003**	98.1	< 0.001	R
		Testis (GC-2 spd, GC-1)	6	-11.11	-17.81,-4.42	**0.001**	79.7	< 0.001	R
		Vessel (HUVECs, rBCEC4)	16	-2.86	-3.87,-1.84	**< 0.001**	53.8	0.005	R
Particle dose	≤ 50 µg/mL		80	-2.09	-2.69,-1.48	**< 0.001**	87.5	< 0.001	R
	> 50 µg/mL		17	-1.80	-3.29,-0.31	**0.018**	88.6	< 0.001	R
	Unknown		4	-0.43	-3.70,2.84	0.798	83.7	< 0.001	R
Particle duration	≤ 24 h		90	-2.00	-2.52,-1.49	**< 0.001**	75.0	< 0.001	R
	> 24 h		11	-0.13	-1.33,1.07	0.836	94.8	< 0.001	R
Assay method	mRNA (RT-PCR)		21	-1.82	-2.90,-0.75	**0.001**	93.3	< 0.001	R
	Protein (WB, IF)		80	-1.76	-2.28,-1.23	**< 0.001**	74.9	< 0.001	R
**Expression of mtDNA maintenance gene DRP1**			125	1.73	1.29,2.18	**< 0.001**	82.4	< 0.001	R
Country	Asian		110	1.99	1.49,2.48	**< 0.001**	82.7	< 0.001	R
	Non-Asian		15	0.41	-0.44,1.26	0.347	71.6	< 0.001	R
Particle type	QDs		6	3.08	1.15,5.02	**0.002**	63.6	0.017	R
	AgNPs		14	4.13	1.41,6.85	**0.003**	84.6	< 0.001	R
	AuNPs		1	6.67	1.78,11.55	0.008	-	-	R
	CBNPs		5	-3.03	-5.31,-0.75	**0.009**	69.3	0.011	R
	FeNPs		4	2.05	-1.15,5.25	0.209	80.2	0.002	R
	NiNPs		9	8.39	5.81,10.97	**< 0.001**	43.2	0.079	R
	PSNPs		28	0.31	-0.36,0.98	0.368	89.2	< 0.001	R
	SiNPs		28	3.03	2.02,4.04	**< 0.001**	69.9	< 0.001	R
	TiO_2NPs		12	1.39	-0.43,3.21	0.133	78.6	< 0.001	R
	ZnONPs		18	2.07	0.76,3.38	**0.002**	78.0	< 0.001	R
Cell type	Species	Human	66	0.91	0.44,1.38	**< 0.001**	72.8	< 0.001	R
		Murine (mouse)	43	5.16	3.97,6.34	**< 0.001**	76.3	< 0.001	R

		Murine (rat)	13	-0.52	-2.56,1.52	0.618	82.6	< 0.001	R
		Murine (total)	56	4.18	2.94,5.41	**< 0.001**	82.2	< 0.001	R
		Porcine	3	2.00	0.65,3.36	**0.004**	97.9	< 0.001	R
	Tissue source	Ovarian (oocytes)	3	2.00	0.65,3.36	**0.004**	97.9	< 0.001	R
		Blood immune (RAW264.7, THP-1)	10	4.98	3.45,6.51	**< 0.001**	26.8	0.197	F
		Bone-muscle (BM-MSCs, MC3T3-E1)	8	-3.14	-5.26,-1.03	**0.004**	72.3	0.001	F
		Neuron (SH-SY5Y, HT22, BV-2, N2a, primary cortical astrocytes, stem cell-derived, neural stem)	44	3.84	2.82,4.86	**< 0.001**	75.9	< 0.001	R
		Breast (HC11)	1	2.15	-0.04,4.34	0.054	-	-	R
		Eye (ARPE-19)	1	4.20	0.88,7.52	0.013	-	-	R
		Heart (AC16)	4	1.89	-0.54,4.32	0.127	73.5	0.010	R
		Liver (L-02, HepG2)	16	1.04	-0.32,2.40	0.135	46.8	0.059	F
		Lung (A549, 16HBE)	6	1.95	0.67,3.22	**0.003**	59.2	0.031	R
		Placenta (HTR-8/SVneo, primary villous cytotrophoblasts)	7	0.33	-0.10,0.75	0.131	0.0	0.761	F
		Stomach (GES-1)	3	-0.93	-3.02,1.16	0.382	67.5	0.046	R
		Testis (GC-1, TM4)	9	8.39	5.81,10.97	**< 0.001**	43.2	0.079	F
		Vessel (HUVECs, rBCEC4)	13	-0.97	-2.36,0.42	0.171	74.4	< 0.001	R
Particle dose	≤ 50 μg/mL		95	1.68	1.16,2.19	**< 0.001**	82.1	< 0.001	R
	> 50 μg/mL		26	3.00	1.84,4.17	**< 0.001**	82.0	< 0.001	R
	Unknown		4	-2.41	-6.26,1.44	0.220	84.0	< 0.001	R
Particle duration	≤ 24 h		113	2.32	1.79,2.85	**< 0.001**	77.7	< 0.001	R
	> 24 h		12	-1.013	-2.190,0.164	0.092	94.1	< 0.001	R
Assay method	mRNA (RT-PCR)		29	0.78	-0.05,1.61	0.066	89.3	< 0.001	R

	Protein (WB, IF)		96	2.21	1.64,2.78	**< 0.001**	77.9	< 0.001	R
**Expression of mtDNA maintenance gene p-DRP1**			49	2.67	1.67,3.67	< 0.001	77.0	< 0.001	R
Particle type	QDs		6	1.65	0.37,2.93	**0.011**	52.3	0.062	R
	AgNPs		12	4.97	3.29,6.65	**< 0.001**	52.5	0.017	R
	CBNPs		1	-3.50	-6.40,-0.59	0.018	-	-	R
	PSNPs		7	6.34	3.38,9.30	**< 0.001**	60.1	0.020	R
	SiNPs		23	0.90	-0.72,2.51	0.278	80.8	< 0.001	R
Cell type	Species	Human	31	1.85	0.39,3.30	**0.013**	80.2	< 0.001	R
		Murine (mouse)	18	3.55	2.24,4.86	**< 0.001**	69.7	< 0.001	R
	Tissue source	Neuron (HT22, BV-2)	15	4.28	2.63,5.93	**< 0.001**	71.9	< 0.001	R
		Liver (HepG2)	13	5.63	3.68,7.59	**< 0.001**	61.7	0.002	R
		Breast (MCF-7)	1	2.74	0.26,5.22	0.031	-	-	R
		Placenta (HTR-8/SVneo)	1	-3.50	-6.40,-0.59	0.018	-	-	R
		Blood immune (RAW264.7)	3	1.66	-0.21,3.53	0.082	55.4	0.106	F
		Lung (16HBE)	8	-1.79	-5.30,1.73	0.319	85.3	< 0.001	R
		Heart (AC16)	8	-0.37	-3.08,2.34	0.787	81.1	< 0.001	R
Particle dose	≤ 50 μg/mL		39	2.74	1.72,3.76	**< 0.001**	74.7	< 0.001	R
	> 50 μg/mL		10	2.53	-0.95,6.02	0.154	84.4	< 0.001	R
Particle duration	≤ 24 h		47	2.60	1.57,3.63	**< 0.001**	77.3	< 0.001	R
	> 24 h		2	5.81	-3.28,14.90	0.210	79.8	0.026	R
**Expression of mtDNA maintenance gene FIS1**			80	0.51	-0.07,1.09	0.084	77.8	< 0.001	R
Country	Asian		74	0.65	-0.05,1.35	0.120	78.9	< 0.001	R
	Non-Asian		6	0.05	-0.55,0.64	0.877	44.9	0.106	F
Particle type	QDs		6	2.77	1.07,4.47	**0.001**	55.3	0.048	R
	AgNPs		9	2.35	-2.46,7.15	0.339	87.2	< 0.001	R

	CBNPs		7	0.77	-1.71,3.26	0.541	81.3	< 0.001	R
	CuONPs		5	21.79	5.72,37.85	**0.008**	84.9	< 0.001	R
	PSNPs		21	-0.86	-1.72,0.01	**0.052**	75.8	< 0.001	R
	NiNPs		5	12.20	6.86,17.54	**< 0.001**	38.9	0.162	F
	SiNPs		14	0.53	-0.40,1.46	0.262	65.5	< 0.001	R
	TiO_2NPs		2	1.72	0.29,3.16	**0.019**	0.0	0.466	F
	ZnONPs		11	-0.27	-1.32,0.79	0.620	69.8	< 0.001	R
Cell type	Species	Human	57	-0.30	-0.85,0.26	0.300	74.2	< 0.001	R
		Murine (mouse)	21	5.16	3.63,6.70	**< 0.001**	67.1	< 0.001	R
		Murine (rat)	2	-5.08	-7.82,-2.34	< 0.001	0.0	0.781	F
		Murine (total)	23	4.50	2.80,6.20	**< 0.001**	76.3	< 0.001	R
	Tissue source	Blood immune (RAW264.7)	3	5.74	1.81,9.68	**0.004**	51.7	0.126	F
		Bone-muscle (BM-MSCs)	2	-5.08	-7.82,-2.34	< 0.001	0.0	0.781	F
		Neuron (SH-SY5Y, HT22, BV-2)	19	0.84	-0.16,1.84	0.100	71.4	< 0.001	R
		Eye (ARPE-19)	1	1.77	-0.25,3.78	0.086	-	-	R
		Heart (HL-1, AC16)	8	0.65	-1.10,2.40	0.468	77.5	< 0.001	R
		Liver (L-02, HepG2)	14	-3.30	-5.29,-1.31	**0.001**	80.8	< 0.001	R
		Lung (A549, 16HBE)	6	1.48	-0.11,3.07	0.068	73.0	0.002	R
		Placenta (HTR-8/SVneo, primary villous cytotrophoblasts)	7	0.04	-0.49,0.57	0.875	34.0	0.169	R
		Stomach (GES-1)	3	-0.66	-3.85,2.53	0.686	80.1	0.007	R
		Testis (GC-1, GC-2spd, TM4)	6	10.38	4.75,16.01	**< 0.001**	71.6	0.003	R
		Vessel (HUVECs, EA.hy926)	11	2.35	-0.43,5.13	0.097	83.8	< 0.001	R
Particle dose	≤ 50 μg/mL		66	0.48	-0.13,1.10	0.125	76.7	< 0.001	R
	> 50 μg/mL		14	0.81	-0.98,2.60	0.376	83.3	< 0.001	R

Particle duration	≤ 24 h		77	0.61	0.02,1.20	**0.042**	77.6	< 0.001	R
	> 24 h		3	-2.60	-7.79,2.59	0.325	87.2	< 0.001	R
Assay method	mRNA (RT-PCR)		7	-1.74	-4.01,0.53	**0.132**	78.7	< 0.001	R
	Protein (WB, IF)		73	0.73	0.13,1.33	**0.017**	77.4	< 0.001	R
**Expression of mitochondrial biogenesis-related gene NRF2 (total)**			248	1.69	1.32,2.05	**< 0.001**	80.4	< 0.001	R
Country	Asian		188	2.10	1.56,2.65	**< 0.001**	81.3	< 0.001	R
	Non-Asian		60	1.14	0.70,1.58	**< 0.001**	77.5	< 0.001	R
Particle type	AgNPs		24	1.86	0.51,3.21	**0.007**	80.7	< 0.001	R
	AuNPs		14	3.95	2.37,5.54	**< 0.001**	66.9	< 0.001	R
	CuNPs		22	5.92	3.94,7.91	**< 0.001**	76.7	< 0.001	R
	NiNPs		10	-2.24	-4.36,-0.12	**0.039**	83.3	< 0.001	R
	PSNPs		55	1.79	1.22,2.35	**< 0.001**	78.2	< 0.001	R
	PdNPs		1	-5.22	-9.17,-1.26	0.010	-	-	R
	QDs		24	1.35	0.30,2.40	**0.012**	74.2	< 0.001	R
	SeNPs		1	21.50	6.52,36.48	0.005	-	-	R
	SiNPs		33	1.69	0.60,2.79	**0.002**	79.0	< 0.001	R
	TiO_2NPs		33	3.05	1.19,4.92	**0.001**	85.4	< 0.001	R
	TiO_2NPs + ZnONPs		2	0.62	-0.05,1.29	0.070	0.0	0.782	F
	ZnONPs		25	1.43	0.63,2.22	**< 0.001**	84.6	< 0.001	R
	FeNPs		4	-0.38	-1.21,0.45	0.369	0.0	0.648	F
Cell type	Species	Human	158	1.45	0.98,1.93	**< 0.001**	79.5	< 0.001	R
		Murine (mouse)	63	1.92	1.14,2.69	**< 0.001**	83.4	< 0.001	R
		Murine (rat)	15	4.16	2.66,5.66	**< 0.001**	56.1	0.004	R
		Murine (total)	78	2.35	1.64,3.06	**< 0.001**	82.1	< 0.001	R
		Fish (gilthead)	6	3.27	1.52,5.02	**< 0.001**	50.8	0.071	R

		Fish (rainbow trout)	5	0.43	0.006,0.85	**0.047**	0.0	0.872	F
		Fish (zebrafish, gilthead, rainbow trout)	11	1.12	0.41,1.84	**0.002**	59.1	0.007	R
		Bovine	1	-0.63	-1.80,0.53	0.287	-	-	R
	Tissue source	Blood immune (RAW264.7, THP-1)	32	0.93	-0.01,1.87	0.053	76.4	< 0.001	R
		Bone-muscle (L6)	2	-2.47	-14.27,9.32	0.681	91.5	0.001	R
		Breast (MDA-MB-231, HC11)	3	14.98	8.88,21.09	**< 0.001**	0.0	0.861	R
		Colorectum (Caco-2, NCM460, BIECs)	5	4.30	0.15,8.46	**0.042**	84.2	< 0.001	R
		Ear (HEI-OC1)	5	17.15	7.14,27.15	**0.001**	69.5	0.011	R
		Eye (ARPE-19, primary corneal endothelial cells)	4	3.97	-3.76,11.70	0.314	86.2	< 0.001	R
		Lung (A549, BEAS-2B, HBE, MeT-5A, MRC-5, RTgill-W1)	34	0.51	-0.13,1.16	0.119	72.1	< 0.001	R
		Heart (H9c2)	5	3.47	2.12,4.81	**< 0.001**	0.0	0.500	F
		Liver (HepG2, AML-12, L-02, QGY, primary hepatocytes)	39	1.46	0.36,2.55	**0.009**	81.3	< 0.001	R
		Neuron (SH-SY5Y, BV2, SaB-1)	16	3.91	2.75,5.08	**< 0.001**	46.7	0.021	F
		Ovarian (SK-OV3, COV434, follicles)	10	2.70	1.15,4.24	**0.001**	90.9	< 0.001	R
		Placenta (HTR-8/SVneo, primary villous cytotrophoblasts)	13	-0.37	-1.58,0.84	0.551	84.8	< 0.001	R
		Skin (HaCaT, HaCaTs, HEK, primary cells)	8	3.13	1.09,5.18	**0.003**	69.8	0.002	R
		Stomach (GES-1)	4	-0.43	-1.91,1.04	0.565	59.2	0.061	R
		Testis (GC-1 spg, GC-2spd, GC-2 spd(ts))	12	1.44	-0.42,3.31	0.129	80.8	< 0.001	R
		Vessel (HUVECs, VSMCs)	56	2.84	1.81,3.86	**< 0.001**	80.4	< 0.001	R
Particle dose	≤ 50 μg/mL		194	1.70	1.29,2.10	**< 0.001**	80.7	< 0.001	R
	> 50 μg/mL		54	1.71	0.88,2.55	**< 0.001**	79.6	< 0.001	R

Particle duration	≤ 24 h		223	1.68	1.28,2.07	**< 0.001**	79.5	< 0.001	R
	> 24 h		25	2.03	1.01,3.05	**< 0.001**	85.0	< 0.001	R
Assay method	mRNA (RT-PCR)		60	0.75	0.17,1.32	**0.011**	81.9	< 0.001	R
	Protein (WB, ELISA, ICC, IF)		188	2.15	1.67,2.63	**< 0.001**	80.0	< 0.001	R
**Expression of mitochondrial biogenesis-related gene NRF2 (nuclear)**			60	1.62	0.99,2.25	**< 0.001**	75.3	< 0.001	R
Country	Asian		29	1.67	0.67,2.67	**0.001**	77.7	< 0.001	R
	Non-Asian		31	1.56	0.74,2.38	**< 0.001**	73.6	< 0.001	R
Particle type	AgNPs		4	-0.82	-4.24,2.60	0.639	88.4	< 0.001	R
	AuNPs		1	11.20	3.28,19.12	0.006	-	-	R
	CBNPs		1	1.62	0.15,3.09	0.031	-	-	F
	CeO_2NPs		3	2.83	1.37,4.30	**< 0.001**	0.0	0.956	F
	CNTs		12	1.23	0.11,12.34	**0.031**	72.7	< 0.001	R
	ND		2	11.42	4.36,18.48	0.002	23.6	0.253	F
	PSNPs		11	0.78	-0.48,2.05	**0.224**	71.2	< 0.001	R
	QDs		8	1.80	-0.52,4.12	0.128	80.2	< 0.001	R
	Si-FeNPs		12	1.63	0.57,2.69	**0.003**	62.1	0.002	R
	SiNPs		5	6.08	3.36,8.81	**< 0.001**	36.8	0.176	F
	TiO_2NPs		1	-3.29	-5.32,-1.25	0.002	-	-	R
Cell type	Species	Human	50	1.50	0.83,2.17	**< 0.001**	75.2	< 0.001	R
		Murine (mouse)	10	1.42	0.88,1.96	**0.027**	78.0	< 0.001	R
	Tissue source	Blood immune (RAW264.7, THP-1)	19	1.36	0.31,2.41	**0.011**	76.0	< 0.001	R
		Neuron (BV2)	5	3.55	1.97,5.14	**< 0.001**	20.8	0.282	F
		Colorectum (Caco-2, HT29)	12	0.89	0.10,1.69	**0.028**	49.6	0.026	R
		Vessel (HUVECs, VSMCs)	4	-0.80	-4.30,2.71	0.655	80.3	0.002	R
		Liver (L-02)	1	-3.29	-5.32,-1.25	0.002	-	-	R

		Lung (A549, BEAS-2B)	8	2.85	0.99,4.71	**0.003**	67.9	< 0.003	R
		Ovarian (KGN)	1	0.00	-1.60,1.60	1.000	-	-	R
		Skin (HaCaT, primaryfacial skin fibroblasts)	5	8.25	0.22,16.28	**0.044**	88.1	< 0.001	R
		Stomach (GES-1)	3	1.52	0.40,2.63	**0.008**	0.0	0.881	F
		Testis (GC-2 spd)	2	3.66	1.53,5.80	0.001	0.0	0.681	F
Particle dose	≤ 50 μg/mL		53	1.75	1.08,2.41	**< 0.001**	74.6	< 0.001	R
	> 50 μg/mL		7	0.70	-1.39,2.01	0.511	82.2	< 0.001	R
Particle duration	≤ 24 h		56	1.40	0.79,1.87	**< 0.001**	74.2	< 0.001	R
	> 24 h		4	10.67	6.80,14.54	**< 0.001**	0.0	0.683	F
Assay method	mRNA (RT-PCR)		4	2.47	-1.10,6.05	0.175	89.5	< 0.001	R
	Protein (WB, IF)		56	1.58	0.93,2.22	**< 0.001**	73.9	< 0.001	R

ND1, NADH dehydrogenase subunit 1; COX1, cytochrome c oxidase subunit 1; COX2, cytochrome c oxidase subunit 2; ATPase 6, ATP synthase F0 subunit 6; CYTB, cytochrome b; PGC-1α, peroxisome proliferator-activated receptor-γ coactivator 1alpha; NRF1, nuclear respiratory factor-1; NRF2, nuclear respiratory factor-2; TFAM, mitochondrial transcription factor A; DRP1, dynamin-related protein 1; FIS1, fission protein 1; MFF, mitochondrial fission factor; MFN1, mitochondrial fusion protein 1; MFN2, mitochondrial fusion protein 2; OPA1, optic atrophy protein 1; AMPK, AMP-activated protein kinase; SIRT1, sirtuin 1; p, phosphorylated; PSNPs, polystyrene NPs; SiNPs, silica NPs; ZnONPs, zinc oxide NPs; AgNPs, silver NPs; QDs, quantum dots; TiO_2NPs, titanium dioxide NPs; FeNPs, iron NPs; PdNPs, palladium NPs; SeNPs, selenium NPs; CBNPs, carbon black NPs; CuONPs, copper oxide NPs; AuNPs, gold NPs; CeO_2NPs, ceria NPs; PtNPs, platinum NPs; NiNPs, nickel NPs; ND, nanodiamonds; nPM, nano-scale particulate matter; SWCNTs, single-walled carbon nanotubes; GO, graphene oxides; MWCNTs, multi-walled carbon nanotubes; RT-PCR, reverse transcription polymerase chain reaction; WB, western blotting; IHC, immunohistochemistry; ICC, immunocytochemistry; IF, immunofluorescence; ELISA, enzyme-linked immunosorbent assay; SMD, standardized mean difference; CI, confidence interval; F, fixed-effects; R, random-effects; P_H-value, significance for heterogeneity; P_{ES}-value, significance for effect size. Bold indicates the indicators with significant results after analysis of more than two datasets.
