# Supplementary material for: Toxicity Evaluation of Nano-Sized Particles by Analysis of mtDNA Content and Expression Levels of Genes Required for mtDNA Maintenance: A Meta-Analysis of Pre-Clinical Studies
Source: Antioxidants (Basel). 2026 Jul 4;15(7):848. doi: 10.3390/antiox15070848 (PMC13405982; doi:10.3390/antiox15070848)
Supplement: Supplementary file 1 [file antioxidants-15-00848-s001.zip › Table S6.pdf]

Table S6 Subgroup meta-analysis for in vivo studies

**Variable**		No.	SMD	95%CI	P_E-value	I^2	P_H-value	Model
**Expression of mitochondrial biogenesis-related gene PGC-1α**		37	-2.43	-3.37,-1.50	**< 0.001**	92.7	< 0.001	R
Country	Asian	8	-4.06	-6.21,-1.91	**< 0.001**	75.9	< 0.001	R
	Non-Asian	29	-2.07	-3.08,-1.06	**< 0.001**	93.6	< 0.001	R
Particle type	AgNPs	5	-4.87	-7.86,-1.89	**0.001**	87.5	< 0.001	R
	Y_2O_3NPs	2	34.66	-55.89,125.21	0.453	93.0	< 0.001	R
	Al_2O_3NPs	4	-3.05	-4.48,-1.62	**< 0.001**	77.8	0.004	R
	ZnONPs	4	-4.44	-7.203,-1.674	**0.002**	91.7	< 0.001	R
	ZnONPs + Al_2O_3NPs	4	-6.62	10.87,-2.36	**0.002**	94.0	< 0.001	R
	QDs	10	1.65	1.15,2.15	**< 0.001**	44.2	0.064	F
	TiO_2NPs	5	-3.84	-5.52,-2.16	**< 0.001**	71.1	0.008	R
	HANPs	1	-0.67	-1.57,0.24	0.148	-	-	R
	PSNPs	1	-1.58	-3.04,-0.12	0.034	-	-	R
	FeONPs	1	-10.00	-15.05,-4.94	< 0.001	-	-	R
Animal type	Murine (mouse)	15	0.23	-0.75,1.22	0.642	85.6	< 0.001	R
	Murine (rat)	21	-4.27	-5.37,-3.18	**< 0.001**	89.0	< 0.001	R
	Murine (total)	36	-2.47	-3.43,-1.51	**< 0.001**	92.9	< 0.001	R
	Zebrafish	1	-1.58	-3.04,-0.12	0.034	-	-	R
Particle dose	≤ 50 mg/kg	19	-0.78	-2.01,0.44	0.211	90.1	< 0.001	R
	> 50 mg/kg	18	-3.60	-4.52,-2.67	**< 0.001**	86.4	< 0.001	R
Particle duration	≤ 14 d	12	1.29	0.58,1.99	**< 0.001**	71.3	< 0.001	R
	> 14 d	25	-4.24	-5.23,-3.26	**< 0.001**	87.7	< 0.001	R
Assay method	mRNA (RT-PCR)	21	-4.10	-5.11,-3.09	**< 0.001**	88.3	< 0.001	R
	Protein (WB, ELISA)	16	0.24	-0.80,1.27	0.656	86.2	< 0.001	R

Tissue source	Liver	14	-0.57	-2.00,0.86	0.433	92.9	< 0.001	R
	Brain	10	-4.21	-6.20,-2.23	**< 0.001**	87.4	< 0.001	R
	Heart	3	-3.56	-4.58,-2.54	**< 0.001**	30.1	0.239	F
	Lung	3	-1.23	-1.79,-0.67	**< 0.001**	0.0	0.922	F
	Thymus	3	-5.17	-6.82,-3.51	**< 0.001**	0.0	0.485	F
	Parotids	4	-2.83	-3.90,-1.76	**< 0.001**	59.0	0.062	R
**Expression of mtDNA maintenance gene MFN2**		23	-1.80	-2.70,-0.90	**< 0.001**	87.5	< 0.001	R
Country	Asian	21	-2.13	-2.99,-1.27	**< 0.001**	83.2	< 0.001	R
	Non-Asian	2	1.59	0.86,2.31	< 0.001	0.0	0.324	F
Particle type	AgNPs	4	-1.66	-4.96,1.64	0.325	93.6	< 0.001	R
	CBNPs	2	-2.41	-7.54,2.72	0.358	88.0	0.004	R
	QDs	2	-4.44	-6.93,-1.95	< 0.001	0.0	0.497	F
	CuNPs	3	-1.66	-2.76,-0.57	**0.003**	68.8	0.040	R
	PSNPs	10	-2.37	-3.40,-1.33	**< 0.001**	76.0	< 0.001	R
	TiO_2NPs	1	1.27	0.30,2.23	0.011	-	-	R
	ZnONPs	1	4.57	2.61,6.52	< 0.001	-	-	R
Animal type	Murine (mouse)	20	-1.36	-2.24,-0.48	**0.002**	86.7	< 0.001	R
	Murine (rat)	1	-7.52	-10.13,-4.91	< 0.001	-	-	R
	Murine (total)	21	-1.62	-2.55,-0.70	**0.001**	88.1	< 0.001	R
	Planarians	2	-4.44	-6.93,-1.95	< 0.001	0.0	0.497	F
Particle dose	≤ 50 mg/kg	21	-2.09	-2.98,-1.20	**< 0.001**	86.2	< 0.001	R
	> 50 mg/kg	2	1.72	-3.82,7.26	0.542	94.3	< 0.001	R
Particle duration	≤ 14 d	11	-1.76	-2.59,-0.93	**< 0.001**	68.5	< 0.001	R
	> 14 d	12	-1.72	-3.30,-0.15	**0.032**	91.9	< 0.001	R
Assay method	mRNA (RT-PCR)	3	2.39	0.85,3.92	**0.002**	77.3	0.012	R

	Protein (WB)	20	-2.35	-3.10,-1.60	**< 0.001**	76.0	< 0.001	R
Tissue source	Brain	3	-1.66	-2.76,-0.57	**0.003**	68.8	0.040	R
	Heart	1	-5.25	-8.53,-1.97	0.002	-	-	R
	Jejunum	1	4.57	2.61,6.52	< 0.001	-	-	R
	Liver	2	-0.86	-2.07,0.35	0.165	0.0	0.726	R
	Lung	10	-2.96	-4.27,-1.66	**< 0.001**	83.9	< 0.001	R
	Placenta	1	-0.00	-1.39,1.39	0.877	-	-	R
	Testis	3	0.51	-1.51,2.53	0.620	88.5	< 0.001	R
	The whole body	2	-4.44	-6.93,-1.95	< 0.001	0.0	0.497	F
**Expression of mtDNA maintenance gene DRP1**		28	2.89	1.88,3.89	**< 0.001**	90.0	< 0.001	R
Country	Asian	24	3.40	2.38,4.42	**< 0.001**	86.2	< 0.001	R
	Non-Asian	4	-0.10	-2.69,2.50	0.943	94.8	< 0.001	R
Particle type	AgNPs	6	2.49	0.41,4.58	**0.019**	90.7	< 0.001	R
	CBNPs	1	-1.06	-2.82,0.70	0.239	-	-	R
	QDs	2	5.58	2.55,8.61	< 0.001	0.0	0.398	F
	NiNPs	6	4.96	3.07,6.84	**< 0.001**	83.4	< 0.001	R
	PSNPs	10	2.67	1.44,3.91	**< 0.001**	81.0	< 0.001	R
	TiO_2NPs	2	-2.55	-6.09,1.00	0.159	92.6	< 0.001	R
	ZnONPs	1	5.23	3.06,7.40	< 0.001	-	-	R
Animal type	Murine (mouse)	23	2.43	1.37,3.50	**< 0.001**	90.3	< 0.001	R
	Murine (rat)	2	5.88	3.51,8.25	< 0.001	57.7	0.124	R
	Murine (total)	25	2.73	1.67,3.79	**< 0.001**	90.8	< 0.001	R
	Zebrafish	1	3.08	1.13,5.03	0.002	-	-	R
	Planarians	2	5.58	2.55,8.61	< 0.001	0.0	0.398	F
Particle dose	≤ 50 mg/kg	26	2.94	1.89,3.98	**< 0.001**	90.1	< 0.001	R

	> 50 mg/kg	2	2.29	-3.38,7.97	0.429	94.2	< 0.001	R
Particle duration	≤ 14 d	9	1.82	0.09,3.55	**0.039**	86.8	< 0.001	R
	> 14 d	19	3.36	2.13,4.59	**< 0.001**	90.7	< 0.001	R
Assay method	mRNA (RT-PCR)	4	1.17	-2.35,4.70	0.514	94.8	< 0.001	R
	Protein (WB, IF)	24	3.17	2.12,4.22	**< 0.001**	88.6	< 0.001	R
Tissue source	Brain	1	3.08	1.13,5.03	0.002	-	-	R
	Heart	1	7.27	4.74,9.80	< 0.001	-	-	R
	Jejunum	1	5.23	3.06,7.40	< 0.001	-	-	R
	Liver	2	-0.57	-1.74,0.59	0.335	62.7	0.030	R
	Lung	10	2.90	1.57,4.23	**< 0.001**	83.8	< 0.001	R
	Placenta	1	-1.06	-2.82,0.70	0.239	-	-	R
	Testis	10	2.98	1.08,4.87	**0.002**	94.0	< 0.001	R
	The whole body	2	5.58	2.55,8.61	< 0.001	0.0	0.398	F
**Expression of mtDNA maintenance gene FIS1**		15	1.71	0.76,2.67	**< 0.001**	75.2	< 0.001	R
Particle type	AgNPs	4	1.04	-1.01,3.09	0.319	86.7	< 0.001	R
	CBNPs	2	2.10	0.70,3.49	0.003	0.0	0.763	F
	Ds	2	5.14	1.33,8.94	0.008	36.1	0.211	F
	CuONPs	2	2.26	0.67,3.85	0.005	0.0	0.725	F
	PSNPs	2	2.16	1.01,3.32	< 0.001	0.0	0.952	F
	SeNPs	2	2.38	-4.22,8.98	0.480	86.8	0.006	R
	ZnONPs	1	-0.39	-1.38,0.60	0.438	-	-	R
Animal type	Murine (mouse)	10	1.01	-0.06,2.08	0.063	70.0	< 0.001	R
	Murine (rat)	2	2.74	1.64,3.84	< 0.001	33.4	0.220	F
	Murine (total)	12	1.39	0.37,2.41	**0.008**	76.8	< 0.001	R
	Zebrafish	1	2.20	0.56,3.84	0.009	-	-	R

	Planarians	2	5.14	1.33,8.94	0.008	36.1	0.211	F
Particle dose	≤ 50 mg/kg	13	2.13	1.27,2.98	**< 0.001**	59.6	0.003	R
	> 50 mg/kg	2	-0.75	-1.89,0.39	0.198	24.9	0.248	F
Particle duration	≤ 14 d	9	1.93	0.65,3.20	**0.003**	72.9	< 0.001	R
	> 14 d	6	1.42	-0.05,2.88	0.058	76.8	0.001	R
Assay method	mRNA (RT-PCR)	2	0.82	-1.72,3.35	0.527	85.7	0.008	R
	Protein (WB)	13	1.89	0.85,2.92	**< 0.001**	71.3	< 0.001	R
Tissue source	Heart	2	3.00	1.87,4.13	< 0.001	0.0	0.367	F
	Liver	2	-0.81	-2.31,0.68	0.287	28.7	0.236	F
	Lung	1	2.25	1.11,3.39	< 0.001	-	-	R
	Placenta	1	1.87	-0.19,3.92	0.076	-	-	R
	The whole body	2	5.14	1.33,8.94	0.008	36.1	0.211	F
	Abdominal aorta	2	2.26	0.67,3.85	0.005	0.0	0.725	F
	Testis	1	2.13	0.51,3.75	0.010	-	-	R
	Brain	1	2.20	0.56,3.84	0.009	-	-	R
	Jejunum	3	0.54	-1.66,2.74	0.629	74.7	0.019	R
**Expression of mitochondrial biogenesis-related gene NRF2 (total)**		185	-0.81	-1.39,-0.24	**0.006**	96.8	< 0.001	R
Country	Asian	135	0.68	0.07,1.29	**0.030**	96.8	< 0.001	R
	Non-Asian	50	-5.72	-7.13,-4.32	**< 0.001**	95.2	< 0.001	R
Particle type	SiNPs	16	-1.55	-3.11,0.02	0.053	98.3	< 0.001	R
	AgNPs	14	-3.24	-4.90,-1.57	**< 0.001**	91.5	< 0.001	R
	Al_2O_3NPs	1	9.99	7.12,12.86	< 0.001	-	-	R
	AuNPs	12	-0.68	-3.24,1.87	0.601	96.0	< 0.001	R
	CBNPs	3	24.11	0.19,48.03	**0.048**	77.1	0.013	R
	CNTs	2	-32.31	-101.11,3649	0.357	98.3	< 0.001	R

	CeO_2NPs	1	9.07	4.9413.20	< 0.001	-	-	R
	CoNPs	9	0.93	-0.041.90	0.060	74.5	< 0.001	R
	CuONPs	4	-15.00	-30.19,0.19	0.053	97.0	< 0.001	R
	FeNPs	6	-1.31	-3.56,0.94	0.254	96.9	< 0.001	R
	Graphene	6	-0.03	-0.70,0.65	0.937	0.0	0.721	F
	MnO_2NPs	2	-0.34	-4.01,3.33	0.857	84.0	0.012	R
	NiNPs	5	-10.07	-14.46,-5.69	**< 0.001**	79.7	0.001	R
	PSNPs	34	-2.96	-4.54,-1.37	**< 0.001**	98.0	< 0.001	R
	QDs	14	3.85	1.76,5.93	**< 0.001**	94.5	< 0.001	R
	SeNPs	1	11.88	7.90,15.86	< 0.001	-	-	R
	TiO_2NPs	46	1.28	-0.38,2.95	0.131	94.7	< .001	R
	ZnNPs + AgNPs	1	-7.21	-10.57,-3.85	< 0.001	-	-	R
	ZnNPs	7	-0.20	-1.70,1.31	0.796	92.6	< 0.001	R
	Fullerenol	1	0.99	-0.50,2.49	0.193	-	-	R
Animal type	Murine (mouse)	62	1.87	0.58,3.16	**0.004**	92.6	< 0.001	R
	Murine (rats)	60	-6.47	-7.80,-5.14	**< 0.001**	94.8	< 0.001	R
	Murine (total)	122	-2.22	-3.15,-1.30	**< 0.001**	93.9	< 0.001	R
	Fish-shellfish (Nile tilapia)	1	-7.20	-8.19,-6.21	< 0.001	-	-	R
	Fish-shellfish (rainbow trout)	2	-4.65	-5.47,-3.83	< 0.001	64.2	0.094	R
	Fish-shellfish (yellow catfish)	2	-0.34	-4.01,3.33	0.857	84.0	0.012	R
	Fish-shellfish (zebrafish)	38	3.50	2.66,4.35	**< 0.001**	98.1	< 0.001	R
	Fish-shellfish (turbots)	4	0.46	-1.85,2.78	0.697	78.3	0.003	R
	Fish-shellfish (Common carp)	7	0.49	-1.35,2.32	0.602	93.8	< 0.001	R
	Fish-shellfish (pearl spot)	3	-38.83	-60.35,-17.33	**< 0.001**	97.1	< 0.001	R
	Fish-shellfish (mussel)	4	-2.62	-8.37,3.12	0.371	86.5	< 0.001	R

	Fish-shellfish (total)	61	1.09	0.26,1.91	**0.010**	98.3	< 0.001	R
	Chicks	2	5.71	-1.57,12.99	0.124	91.6	0.001	R
Particle dose	≤ 50 mg/kg	161	-0.06	-0.53,0.65	0.835	96.9	< 0.001	R
	> 50 mg/kg	24	-7.77	-10.07,-5.46	**< 0.001**	94.0	< 0.001	R
Particle duration	≤ 14 d	71	0.10	-0.73,0.93	0.815	98.2	< 0.001	R
	> 14 d	114	-1.53	-2.40,-0.65	**0.001**	94.0	< 0.001	R
Assay method	mRNA (RT-PCR)	105	-0.22	-0.89,0.46	0.526	97.1	< 0.001	R
	Protein (WB, IF, IHC, ELISA)	80	-1.46	-2.47,-0.45	**0.005**	94.8	< 0.001	R
Tissue source	Spleen	7	5.83	0.50,11.17	**0.032**	89.3	< 0.001	R
	Aorta	6	1.07	-1.93,4.07	0.484	87.0	< 0.001	R
	Brain	30	-2.66	-4.29,-1.03	**0.001**	95.9	< 0.001	R
	Bursa of Fabricius	2	5.71	-1.57,12.99	0.124	91.6	0.001	R
	Cochlea	2	9.72	4.43,15.01	< 0.001	6.5	0.301	F
	Heart	2	-2.55	-5.73,0.64	0.117	87.9	0.004	R
	Intestine	7	0.69	-1.47,2.86	0.530	90.6	< 0.001	R
	Kidney	15	-7.87	-10.99,-4.76	**< 0.001**	95.3	< 0.001	R
	Liver	44	-5.69	-7.13,-4.24	**< 0.001**	96.7	< 0.001	R
	Lung	25	4.50	1.73,7.28	**0.001**	91.8	< 0.001	R
	Muscle	2	-0.53	-1.70,0.64	0.376	0.0	0.580	F
	Ovary	2	-8.37	-24.16,7.43	0.299	84.6	0.011	R
	Pancreas	4	-1.51	-7.80,4.78	0.638	97.2	< 0.001	R
	Eye	2	-3.32	-5.43,-1.21	0.002	4.8	0.305	F
	Testis	7	-5.02	-8.99,-1.05	**0.013**	91.7	< 0.001	R
	The total body	28	4.62	3.62,5.62	**< 0.001**	98.6	< 0.001	R

PGC-1α, peroxisome proliferator-activated receptor-γ coactivator 1alpha; NRF2, nuclear respiratory factor-2; DRP1, dynamin-related protein 1; FIS1, fission protein 1; MFN2, mitochondrial fusion protein 2; NPs, nanoparticles; PSNPs, polystyrene NPs; SiNPs, silica NPs; ZnONPs, zinc oxide NPs; AgNPs, silver NPs; QDs, quantum dots; TiO_2NPs, titanium dioxide NPs; FeNPs,

iron NPs; SeNPs, selenium NPs; Y_2O_3NPs, yttrium oxide NPs; CBNPs, carbon black NPs; CuONPs, copper oxide NPs; AuNPs, gold NPs; CeO_2NPs, ceria NPs; NiNPs, nickel NPs; CoNPs, cobalt NPs; CNTs, carbon nanotubes; MnO_2NPs, manganese dioxide NPs; Al_2O_3NPs, aluminum oxide NPs; HANPs, hydroxyapatite NPs; RT-PCR, reverse transcription polymerase chain reaction; WB, western blotting; IHC, immunohistochemistry; ICC, immunocytochemistry; IF, immunofluorescence; ELISA, enzyme-linked immunosorbent assay; SMD, standardized mean difference; CI, confidence interval; F, fixed-effects; R, random-effects; P_{H}-value, significance for heterogeneity; P_{ES}-value, significance for effect size. Bold indicates the indicators with significant results after analysis of more than two datasets.
