# Supplementary material for: Toxicity Evaluation of Nano-Sized Particles by Analysis of mtDNA Content and Expression Levels of Genes Required for mtDNA Maintenance: A Meta-Analysis of Pre-Clinical Studies
Source: Antioxidants (Basel). 2026 Jul 4;15(7):848. doi: 10.3390/antiox15070848 (PMC13405982; doi:10.3390/antiox15070848)
Supplement: Supplementary file 1 [file antioxidants-15-00848-s001.zip › Table S7.pdf]

Table S7 Meta-regression analysis for in vitro studies

Variable		Coef.	Std. Err.	Z	*P*	95%CI
**mtDNA content**						
Country		-1.333	1.714	-0.78	0.437	-4.692,2.023
Particle type		-0.118	0.221	-0.53	0.594	-0.551,0.316
Cell type	Species	-0.958	1.549	-0.62	0.536	-3.994,2.078
	Tissue source	0.007	0.103	0.07	0.943	-0.194,0.209
Particle dose		0.349	0.894	0.39	0.696	-1.402,2101
Particle duration		0.618	1.963	0.31	0.753	-3.229,4.464
Assay method of mtDNA-encoded genes		-0.018	0.077	-0.24	0.810	-0.169,0.120
**Expression of mitochondrial biogenesis-related gene PGC-1α**						
Country		-0.215	1.194	-0.18	0.857	-2.556,2.127
Particle type		0.109	0.277	0.39	0.694	-0.434,0.652
Cell type	Species	0.307	0.860	0.36	0.721	-1.379,1.992
	Tissue source	0.041	0.113	0.36	0.718	-0.181,0.263
Particle dose		0.560	1.886	0.30	0.767	-3.136,4.256
Particle duration		0.357	1.694	0.21	0.833	-2.964,3.678
Assay method		-0.150	0.712	-0.21	0.833	-1.546,1.245
**Expression of mtDNA maintenance gene MFN1**						
Country		-0.316	0.389	-0.81	0.416	-1.079,0.446
Particle type		0.010	0.070	0.14	0.890	-0.127,0.146
Cell type	Species	-0.037	0.310	-0.12	0.906	-0.645,0.572
	Tissue source	-0.014	0.049	-0.29	0.775	-0.111,0.083
Particle dose		0.007	0.254	0.030	0.980	-0.492,0.505
Particle duration		0.076	0.741	0.10	0.919	-1.376,1.527
Assay method		-0.098	0.399	-0.24	0.807	-0.879,0.684
**Expression of mtDNA maintenance gene MFN2**						
Country		-0.273	0.153	-1.79	0.074	-0.572,0.026
Particle type		-0.005	0.049	-0.11	0.915	-0.101,0.090
Cell type	Species	0.021	0.169	0.12	0.903	-0.311,0.352
	Tissue source	-0.002	0.028	-0.08	0.938	-0.057,0.053
Particle dose		0.020	0.253	0.08	0.936	-0.475,0.515
Particle duration		0.011	0.708	0.02	0.988	-1.378,1.399
Assay method		-0.015	0.210	-0.07	0.943	-0.428,0.397
**Expression of mtDNA maintenance gene OPA1**						
Country		-0.205	0.140	-1.47	0.142	-0.479,0.069
Particle type		0.009	0.051	0.17	0.865	-0.091,0.109
Cell type	Species	0.124	0.305	0.41	0.685	-0.474,0.722
	Tissue source	0.018	0.035	0.51	0.607	-0.051,0.087
Particle dose		0.084	0.270	0.31	0.756	-0.446,0.614
Particle duration		-0.356	0.918	-0.39	0.698	-2.155,1.443
Assay method		-0.019	0.551	-0.03	0.973	-1.098,1.060
**Expression of mtDNA maintenance gene DRP1**						

Country		-0.281	0.137	-2.060	**0.039**	-0.549,-0.014
Particle type		0.018	0.050	0.360	0.716	-0.080,0.117
Cell type	Species	0.149	0.448	0.330	0.740	-0.729,1.026
	Tissue source	0.006	0.040	0.150	0.883	-0.073,0.084
Particle dose		0.062	0.185	0.340	0.738	-0.300,0.424
Particle duration		-0.696	0.896	-0.780	0.437	-2.451,1060
Assay method		-0.175	0.479	-0.360	0.716	-1.114,0.765
**Expression of mtDNA maintenance gene p-DRP1**						
Particle type		0.053	0.348	0.15	0.879	-0.628,0.734
Cell type	Species	-0.166	1.472	-0.11	0.910	-3.052,2.720
	Tissue source	-0.130	0.222	-0.59	0.556	-0.565,0.304
Particle dose		0.279	1.946	0.14	0.886	-3.535,4.092
Particle duration		-0.563	2.547	-0.22	0.825	-5.555,4.428
**Expression of mtDNA maintenance gene FIS1**						
Country		-0.312	0.351	-0.89	0.374	-1.001,0.376
Particle type		-0.005	0.152	-0.04	0.972	-0.304,0.293
Cell type	Species	0.280	1.224	0.23	0.819	-2.119,2.680
	Tissue source	-0.001	0.073	-0.01	0.992	-0.143,0.141
Particle dose		0.071	0.483	0.15	0.883	-0.876,1.019
Particle duration		0.225	1.676	0.13	0.893	-3.061,3.510
Assay method		-0.156	0.747	-0.21	0.834	-1.620,1.307
**Expression of mitochondrial biogenesis-related gene NRF2 (total)**						
Country		-0.267	0.123	-2.160	**0.030**	-0.509,-0.025
Particle type		-0.004	0.020	-0.190	0.849	-0.042,0.035
Cell type	Species	-0.111	0.110	-1.010	0.314	-0.326,0.104
	Tissue source	-0.027	0.011	-2.530	**0.012**	-0.048,-0.06
Particle dose		-0.008	0.121	-0.070	0.944	-0.246,0.229
Particle duration		0.054	0.409	0.130	0.895	-0.747,0.856
Assay method		-0.147	0.096	-1.540	0.125	-0.334,0.041
**Expression of mitochondrial biogenesis-related gene NRF2 (nuclear)**						
Country		0.023	0.448	0.05	0.959	-0.855,0.901
Particle type		0.010	0.071	0.15	0.884	-0.128,0.149
Cell type	Species	0.136	0.666	0.20	0.838	-1.169,1.441
	Tissue source	0.041	0.112	0.37	0.713	-0.178,0.261
Particle dose		-0.020	0.540	-0.04	0.971	-1.078,1.039
Particle duration		2.744	5.501	0.50	0.618	-8.039,13.526
Assay method		0.141	0.423	0.33	0.740	-0.689,0.970

PGC-1α, peroxisome proliferator-activated receptor-γ coactivator 1alpha; NRF2, nuclear respiratory factor-2; DRP1, dynamin-related protein 1; FIS1, fission protein 1; MFN1, mitochondrial fusion protein 1; MFN2, mitochondrial fusion protein 2; OPA1, optic atrophy protein 1; Std, standard; Err, error; CI, confidence interval. Bold indicates the indicators with significant results.
