# Supplementary material for: Toxicity Evaluation of Nano-Sized Particles by Analysis of mtDNA Content and Expression Levels of Genes Required for mtDNA Maintenance: A Meta-Analysis of Pre-Clinical Studies
Source: Antioxidants (Basel). 2026 Jul 4;15(7):848. doi: 10.3390/antiox15070848 (PMC13405982; doi:10.3390/antiox15070848)
Supplement: Supplementary file 1 [file antioxidants-15-00848-s001.zip › Table S8.pdf]

Table S8 Meta-regression analysis for in vivo studies

Variable	Coef.	Std. Err.	Z	*P*	95%CI
**Expression of mitochondrial biogenesis-related gene PGC-1α**					
Country	-0.842	2.508	-0.34	0.737	-5.758,4.074
Particle type	-0.034	0.302	-0.11	0.910	-0.627,0.558
Animal type	-0.997	5.248	-0.19	0.849	-11.283,9.9
Particle dose	-0.445	3.170	-0.14	0.888	-6.658,5.767
Particle duration	0.156	4.483	0.03	0.972	-8.630,8.942
Assay method	-0.307	5.865	-0.05	0.958	-11.802,11.187
Tissue source	-0.015	0.388	-0.04	0.968	-0.775,0.745
**Expression of mtDNA maintenance gene MFN2**					
Country	-0.751	5.032	-0.15	0.881	-10.613,9.111
Particle type	-0.068	0.189	-0.36	0.721	-0.438,0.303
Animal type	0.885	3.313	0.27	0.789	-5.608,7.378
Particle dose	0.067	1.248	0.05	0.957	-2.379,2.513
Particle duration	-0.018	0.537	-0.03	0.973	-1.072,1.035
Assay method	-0.560	4.828	-0.12	0.908	-10.023,8.902
Tissue source	0.042	0.223	0.19	0.850	-0.395,0.480
**Expression of mtDNA maintenance gene DRP1**					
Country	-0.297	1.325	-0.22	0.823	-2.893,2.299
Particle type	-0.043	0.199	-0.22	0.828	-0.433,0.346
Animal type	0.594	1.752	0.34	0.734	-2.839,4.027
Particle dose	0.013	0.779	0.02	0.987	-1.514,1.59
Particle duration	0.086	1.066	0.08	0.936	-2.004,2.176
Assay method	0.165	1.392	0.12	0.906	-2.563,2.893
Tissue source	0.019	0.382	0.05	0.96	-0.729,0.767
**Expression of mtDNA maintenance gene FIS1**					
Particle type	0.168	1.312	0.13	0.898	-2.404,2.740
Animal type	0.539	1.527	0.35	0.724	-2.454,3.533
Particle dose	0.103	1.561	0.07	0.947	-2.956,3.162
Particle duration	-0.248	1.947	-0.13	0.899	-4.064,3.567
Assay method	0.637	1.737	0.37	0.714	-2.767,4.042
Tissue source	-0.077	0.810	-0.09	0.925	-1.663,1.510
**Expression of mitochondrial biogenesis-related gene NRF2 (total)**					
Country	0.431	0.334	1.29	0.197	-0.223,1.084
Particle type	0.008	0.029	0.26	0.795	-0.049,0.064
Animal type	0.191	0.166	1.15	0.251	-0.135,0.517
Particle dose	0.244	0.522	0.47	0.641	-0.780,1.267
Particle duration	0.444	0.281	1.58	0.115	-0.107,0.995
Assay method	0.119	0.395	0.30	0.763	-0.655,0.893
Tissue source	0.009	0.046	0.20	0.841	-0.080,0.098

PGC-1α, peroxisome proliferator-activated receptor-γ coactivator 1alpha; NRF2, nuclear respiratory factor-2; DRP1, dynamin-related protein 1; FIS1, fission protein 1; MFN2, mitochondrial fusion protein 2; Std, standard;

Err, error; CI, confidence interval. Bold indicates the indicators with significant results.
